# In sync through laughter? An fNIRS hyperscanning study on neural synchrony and social connection

**DOI:** 10.3389/fnins.2025.1697100

**Published:** 2026-01-20

**Authors:** Verena T. Schäfer, Stefanie Hoehl, Carolina Pletti

**Affiliations:** 1 Faculty of Psychology, University of Vienna, Vienna, Austria; 2 Vienna Doctoral School Cognition, Behavior and Neuroscience, University of Vienna, Vienna, Austria

**Keywords:** Bayesian analyses, bonding, hyperscanning, interpersonal synchrony, laughter, liking, prosociality

## Abstract

Laughter is a widespread social behavior that has been associated with increases in social connection. However, the mechanisms behind this link are not yet well understood. We hypothesized that laughter supports positive social outcomes by enhancing neural synchrony during social interactions. Neural synchrony is a process of mutual alignment of brain areas, which has been shown to positively affect social interactions. In a 2 × 2 design, participant pairs watched either funny or neutral videos (Laughter Manipulation: yes/no), either together or separately (Social Context: yes/no). Afterwards, they engaged in a 10-minute free interaction. Laughter behavior was annotated during both phases. Neural synchrony was measured using fNIRS hyperscanning for both phases and quantified across frontal and temporal regions using Wavelet Transform Coherence. As social outcomes, we measured Liking, Prosociality, and Bonding after the free conversation. We tested our hypotheses with Bayesian models that assessed the effects of Laughter and Social context on social outcomes, with synchrony modeled as a mediator. Parameter estimates for the effects of Laughter and Social Context on interpersonal neural synchrony were close to zero, with Bayes Factors indicating evidence for the null hypothesis. Similarly, the effects of Laughter and Social Context on Liking, Prosociality, and Bonding showed no effects. However, model comparisons provided evidence for annotated Laughter Behavior as a predictor of Liking, Prosociality, and Bonding. Mediation analyses revealed no overall effect, but some findings stood out. We observed a negative association between right IFG and right TPJ synchrony during the manipulation phase and later Liking, and a positive association between right and left IFG synchrony and subsequent Prosociality. Additionally, synchrony during the free interaction phase between the left IFG and right TPJ predicted Liking and synchrony between the left and right TPJ predicted Bonding. In total, our findings show no direct link between Laughter and neural synchrony. However, Laughter Behavior was associated with social outcomes. Additionally, neural synchrony was also linked to social outcomes, with distinct positive and negative associations depending on the brain regions involved. These results highlight the complexity of the relationship between laughter, neural synchrony, and social connection, suggesting the need for further research.

## 1 Introduction

Think back to the last time you shared a laugh with someone—one of those moments when you couldn’t help but feel closer to them. What is it about laughter that creates this connection, aligning not just our emotions but perhaps even our minds?

Laughter is a nonverbal and multimodal phenomenon that combines vocal expressions, body movements, and facial gestures (Vettin and Todt, 2004). It serves as a communicative signal (Sauter et al., 2010a) and is recognized across cultures as a positive emotional expression (Sauter et al., 2010b). Laughter is inherently social; people are 30 times more likely to laugh in the presence of others than when alone (Provine, 2000; Provine and Fischer, 1989). It is rhythmic and contagious, often spreading quickly among group members (Provine, 1992; Scott et al., 2022). Laughter seems to play a crucial role in maintaining social bonds: Dunbar (2012, 2022) hypothesizes that laughter serves as the human equivalent of grooming in primates, providing a foundation for its bonding function. Grooming activates the endorphin release system, strengthening social bonds within groups (Dunbar, 2012, 2022). However, as human groups expanded in size during evolution, physical grooming became less practical. Laughter, according to Dunbar (2012, 2022), evolved to fulfill this bonding function, enabling individuals to maintain social cohesion and strengthen affiliative ties without direct physical contact. In support of this theory, research has shown that laughter triggers the endogenous opioid release system, which is associated with feelings of pleasure and connection (Manninen et al., 2017). This physiological mechanism reinforces the role of laughter in fostering social bonds (Dunbar, 2022; Machin and Dunbar, 2011). Furthermore, research by Bryant et al. (2016) suggests that laughter may convey meaningful social information. For example, the authors found that listeners across a range of cultures were able to infer the relationship between individuals based solely on the sound of their shared laughter, reliably distinguishing between friends and strangers. Furthermore, listeners tended to assume that individuals who laughed together as friends liked each other more than those perceived as strangers. These findings suggest that laughter could be associated with liking, pointing to its potential role in shaping social affiliations. Together, these findings support the view that laughter is not only a signal of emotional expression but also a powerful mechanism for fostering social connectedness and facilitating positive interpersonal outcomes.

While laughter has been proposed to contribute to social connections (Dunbar, 2022; Scott et al., 2022), the mechanisms underpinning these social effects of laughter are not yet well understood. Laughter has been described as a social coupling mechanism that aligns emotional and behavioral states across individuals (Gervais and Wilson, 2005; Provine, 1992). Building on this perspective, we argue that understanding the mechanisms through which laughter influences social connection requires a closer examination of underlying neural processes. As Gervais and Wilson (2005) hypothesized, laughter may be linked to mirror systems, coordination, and synchrony. Given laughter’s inherently rhythmic structure and strong social salience, we propose that interpersonal neural synchrony may be one of the processes through which laughter exerts its positive effects on social interaction. Interpersonal neural synchrony—the rhythmic and dynamic alignment of brain activity between individuals—is a key factor in shared experiences and coordinated interactions and contributes to the emergence of positive social connection (De Felice et al., 2024; Gordon et al., 2024; Ohayon and Gordon, 2025; Roche et al., 2024). Synchrony in this context refers to the extent to which two or more signals are temporally related (De Felice et al., 2024). This includes signals that are aligned in time, either perfectly simultaneous or with slight temporal shifts, reflecting a shared and coordinated process (Nguyen et al., 2021). A common and effective technique for studying neural synchrony is fNIRS hyperscanning, which simultaneously measures brain activity in interacting individuals (De Felice et al., 2024; Nguyen et al., 2021), capturing inter-brain processes in real time (De Felice et al., 2024; Schirmer et al., 2021). When applied with mobile brain recording devices, it allows participants to engage naturally, such as sitting face-to-face, providing an ecologically valid representation of social interactions (De Felice et al., 2024; Roche et al., 2024).

Previous research has linked interpersonal (neural) synchrony to liking between interaction partners (Ravreby et al., 2022), prosocial tendencies (Mogan et al., 2017), and social bonds (Schirmer et al., 2021). *Liking* refers to the extent to which an interaction partner is perceived as socially appealing. Hove and Risen (2009) demonstrated that participants who engaged in synchronous movements with an experimenter rated them as more likable than those in asynchronous or Control conditions. Another study also showed that participants liked a pre-recorded partner more when moving in synchrony than when moving out of synchrony (Lang et al., 2017). Similarly, Ravreby et al. (2022) found that higher movement synchrony in a Mirror Game was associated with increased liking. Complementing these behavioral findings, Lei et al. (2025) showed that neural synchrony in the right temporoparietal junction (TPJ) during cooperative interactions predicted greater outgroup liking, pointing to a potential link between neural synchrony and social evaluation.

*Prosocial* behavior refers to voluntary actions that benefit others, such as helping or sharing. Hu et al. (2017) showed that synchrony increased participants’ willingness to help their interaction partner in a hypothetical helping scenario, and that this effect was mediated by interbrain synchrony in the left middle frontal cortex, as measured with fNIRS hyperscanning. The authors conclude that interpersonal synchrony facilitates prosocial behavior. Additionally, Kokal et al. (2011), using fMRI, found that participants who drummed in synchrony with a partner showed increased activation in the bilateral caudate, a reward-related region in the striatum. This activity predicted prosocial behavior, measured by how many pencils participants picked up in a helping task. As fMRI allows access to deep brain regions not measurable with fNIRS, this finding provides important theoretical evidence linking synchrony, reward processing, and prosocial behavior. Supporting this, a meta-analysis by Mogan et al. (2017) reported a medium-sized overall effect of synchrony on prosocial behavior across a wide range of experimental studies.

Moreover, synchrony has been proposed to support the formation of *social bonds* (Feldman, 2017; Hoehl et al., 2021). While direct measures of bonding are rare, proxy constructs such as rapport have been used to assess perceived connectedness. For instance, observers rated dyads who moved in synchrony as experiencing greater rapport, a construct closely related to bonding, than those who did not (Miles et al., 2009). Hoehl et al. (2021) further argue that synchrony and bonding mutually reinforce each other, describing synchrony as a mechanism that promotes affiliative engagement and social cohesion. This is supported by recent experimental work showing that synchrony during shared experiences predicts participants self-reported feelings of social connection (Cheong et al., 2023).

In summary, both laughter and interpersonal synchrony have independently been linked to positive social and emotional outcomes, but their interaction has rarely been studied. However, laughter’s rhythmic and multimodal characteristics (Gervais and Wilson, 2005; Provine, 1992; Scott et al., 2022; Vettin and Todt, 2004) have been theorized to be a compelling facilitator of interpersonal synchrony. When people laugh together, their behaviors and emotional states align, reinforcing feelings of affiliation and belonging. Gordon et al. (2024) emphasize that laughing together is in itself “*a moment of interpersonal synchrony*,” highlighting its role in aligning emotional and behavioral states. A study by Ludusan and Wagner (2022) found increased temporal synchrony between interacting partners in their production of laughter during a conversational setting. Additionally, Cheong et al. (2023) investigated how the temporal and spatial alignment of positive facial expressions enhances bonding and affiliation within dyads. Since laughter inherently involves similar positive expressions and is a very social cue, it likely contributes to bonding in a comparable way by fostering synchronized emotional experiences. Studies have shown that synchronized activities, which involve coordinated bursts of sound and movement, promote bonding by creating a shared rhythm (Hoehl et al., 2021), which enhances cooperative behavior and prosociality (Dunbar, 2012; Gvirts and Perlmutter, 2020; Tarr et al., 2015). While laughter shares similar rhythmic properties, its role in interpersonal neural synchrony remains to be empirically tested.

To explore this question further, it is helpful to consider the neural mechanisms that underlie both laughter and interpersonal neural synchrony. Specifically, brain regions in the frontal and temporal areas play a crucial role in the processing of interpersonal synchrony, rhythmical stimuli, and social information. Frontal regions, particularly the inferior frontal gyrus (IFG), are critical for neural synchrony (Kanterman and Shamay-Tsoory, 2025; Ravreby et al., 2022; Shamay-Tsoory et al., 2019). In the social alignment model by Shamay-Tsoory et al. (2019), the IFG is part of a system that helps individuals adjust their behavior, emotions, or thoughts in response to others. This process involves monitoring the social gap between self and others and coordinating responses to reduce it, which is thought to support social closeness. The authors argue that the IFG is consistently activated during synchrony-related tasks and therefore propose that its role in regulating social closeness may be closely linked to its involvement in synchrony, as part of a shared neural mechanism for social alignment (Shamay-Tsoory et al., 2019). The IFG’s activation during laughter further highlights its role in processing and responding to this social cue (Nummenmaa et al., 2023). Furthermore, a study by Li et al. (2022) investigated whether watching emotionally positive videos together, as opposed to neutral videos, increases interpersonal neural synchrony in the IFG, suggesting that shared exposure to emotionally engaging positively valenced content may enhance neural synchrony in this brain region.

Temporal brain regions also contribute significantly to neural synchrony, social processing, and laughter. The superior temporal cortex is implicated in the processing of rhythmic information, which could combine rhythmic and social cues (Schirmer et al., 2016). The superior temporal gyrus (STG) is particularly engaged when individuals listen to genuine laughter, as opposed to voluntary laughter, emphasizing its auditory and social dimensions (McGettigan et al., 2015). The posterior STG exhibits patterns of neural synchrony during communicative interactions (Dikker et al., 2014). These findings highlight the STG’s role in processing the auditory features of laughter and supporting neural synchrony, making it a key region for studying their interplay. Furthermore, the TPJ has also been proposed to be involved in interpersonal neural synchrony (Shamay-Tsoory et al., 2019). Corroborating this notion, Czeszumski et al. (2022) show in their meta-analysis that synchronized TPJ activity can be consistently found during social interactions, which involve cooperative tasks (e.g., joint problem solving). Gvirts and Perlmutter (2020) suggest that the TPJ is involved in interpersonal brain synchrony through its role in coordinating mutual social attention between interaction partners. The TPJ also has been proposed to support the prediction of outcomes in social contexts (Kanterman and Shamay-Tsoory, 2025), suggesting its broader involvement in affiliative and synchrony-related processes. Taken together, these findings suggest that frontal areas such as the IFG, and temporal areas such as STG and TPJ play a central role in linking laughter, synchrony, and positive social outcomes.

The current study builds on this theoretical foundation by investigating how laughter and interpersonal synchrony contribute to positive social outcomes in human interaction, including liking, prosociality and bonding. However, laughter research is still in its early stages, with many open questions about its neural underpinnings and its function in promoting social behaviors (Provine, 2016; Zauli et al., 2022). While a growing body of hyperscanning research has shown that rhythmic social behaviors such as speech or touch can foster interpersonal synchrony and promote positive social interactions (De Felice et al., 2024; Markova et al., 2019), laughter, despite being inherently rhythmic, has not yet been studied in this context. This represents a critical gap in the current literature. We hypothesize that laughter, which is a positive social rhythmic signal, affects synchrony, which uses rhythmical cues and supports liking, prosociality and bonding between people (Dunbar, 2012; Gervais and Wilson, 2005; Gordon et al., 2024; Mogan et al., 2017; Provine, 1992). To investigate the proposed connection between laughter, synchrony and the social outcomes, it is essential first to establish how laughter fosters synchrony: for instance, is it sufficient for people to hear laughter in order to synchronize their brain activity to it? Or does laughter need to be embedded in a social interaction, in order to facilitate neural synchrony between interacting people? This leads to our first research question: Does laughter increase interpersonal neural synchrony? In order to better understand the mechanisms through which laughter may facilitate synchrony, we divided this question into two complementary hypotheses: according to the first, due to the rhythmic properties of laughter, it would be enough for people to hear laughter at the same time, even without an interactive context, for them to subsequently show increased neural synchrony. According to the second hypothesis, due to the social characteristics of laughter, only shared laughter experiences during a social interaction should increase neural synchrony between partners. In this study, shared laughter experiences are defined as situations where both participants engage in a funny shared social experience that involves laughter.

To test these hypotheses, we use a 2 × 2 factorial design, manipulating both laughter exposure and occurrence (through funny or neutral tasks) and social context (having participants perform such tasks either together or separately) while also examining interpersonal neural synchrony and participants’ actual laughter behavior during this manipulation phase. Immediately after this manipulation phase, we test the persistent effects of laughter and social context by measuring interpersonal neural synchrony and laughter behavior while participants have a free conversation. This factorial approach allows for more specific insights into the mechanisms through which laughter may influence interpersonal neural synchrony, which we formalize in the following hypotheses:

*H1a: Laughter increases interpersonal neural synchrony in adult dyads, irrespective of whether it’s embedded in a social interaction or not.*

*H1b: Laughter increases interpersonal neural synchrony in adult dyads only when embedded in a social context.*

We next ask whether laughter exposure and occurrence also affect social outcomes such as liking, prosociality and bonding. Existing studies suggest that laughter enhances social connections (Dunbar, 2012, 2022). However, the underlying mechanisms and their specific role in fostering bonding remain unclear (Scott et al., 2022). This leads to our second research question: Does laughter as a shared experience promote liking, prosociality and bonding between interaction partners? To address this question, we formulated the following hypothesis:

*H2a: Laughter, regardless of an interaction context, leads to more liking, prosociality and bonding.*

*H2b: Laughter leads to more liking, prosociality and bonding only when embedded in a social context.*

The third research question investigates whether neural synchrony mediates the relationship between laughter and positive social outcomes such as liking, prosocial behavior and bonding. Building on existing research suggesting that synchrony strengthens social outcomes such as prosocial behavior, social evaluation, and helping tendencies (Hu et al., 2017; Kokal et al., 2011; Lei et al., 2025), this study extends this line of research by focusing on laughter as a socially engaging and rhythmically structured behavior. By proposing a mediation model, this hypothesis aims to clarify how laughter, synchrony, and positive social outcomes are interrelated. This integrated perspective is reflected in the following hypothesis.

*H3: The link between laughter and prosociality, liking, and bonding is mediated through neural synchrony.*

## 2 Materials and methods

### 2.1 Participants

Participants were recruited through two student recruitment systems at the University of Vienna. Individuals with neurological or physiological conditions were excluded from participation.

The required sample size was determined using a sequential hypothesis testing Bayesian approach (Schönbrodt et al., 2017). This approach follows Bayesian sequential testing logic, where data collection is guided by predefined evidence thresholds rather than fixed sample sizes, allowing for flexible and resource-sensitive study designs (Visser et al., 2023). Testing began with 20 dyads per condition and continued until a Bayes factor of BF > 5 or BF < 0.20 was reached or the end of resources (money, time) was reached. Data collection was terminated when available resources (funding and time) were exhausted, before conclusive Bayes factors could be reached for all comparisons. We needed to exclude one testing because a participant had already participated in the study, one dyad did not talk during the free conversation task, and two testings because of experimenter error (the wrong manipulation videos were shown). The final sample consists of 196 participants, totaling 98 dyads, including 126 females (63 dyads) and 78 males (39 dyads). We measured same-gender dyads because prior research suggests that males and females differ in their laughter behavior (Dunbar et al., 2021; Provine, 1993). Participants’ ages ranged from 18 to 25 years (*M* = 21.5, *SD* = 2.0). Most participants were right-handed (*n* = 153), while a smaller number identified as left-handed (*n* = 17). All participants provided written informed consent before the study. Upon completing the experiment, participants were debriefed and compensated with either €15 or, for psychology students, eight course credits. The study procedure was preregistered, and ethical approval for the study was obtained in advance from the Ethics Committee of the University of Vienna.

### 2.2 Measurements

#### 2.2.1 Laughter

Research on laughter within experimental designs is still relatively novel, and we aimed to address this research gap by employing a multifaceted approach to capture laughter through both experimental manipulation and direct behavioral annotations. First, we implemented a laughter manipulation as part of our 2 × 2 experimental design [Laughter (laughter vs. no laughter) × Social context (interaction vs. no interaction) (see section 2.3.3)]. Additionally, we examined participants’ actual laughter behavior. The duration of laughter was annotated by five coders with the software ELAN (Sloetjes and Wittenburg, 2008). Coders were trained on an average of 4.6 pilot testing sessions (ranging from 1 to 7) and, for the assessment of interrater reliability, additionally coded 5–10% of the actual testing sessions, which overlapped with at least one other coder. The average interrater agreement of 0.74 was calculated with the Staccato algorithm (Lücking et al., 2011), which is implemented in ELAN. The annotation scheme was adapted from Mazzocconi et al. (2020). For the laughter annotation, the onset and offset of laughter were coded for each participant. For each dyad, total laughter duration was calculated as the sum of all time periods during which at least one participant was laughing. Based on these annotations, the total duration of laughter was calculated for each experimental phase and dyad. Each experimental condition included 24 dyads. For the mean and standard deviation of laughter duration (see Table 1). We defined each experimental phase as the task interval plus a brief pre-onset window; laughter occurring between two tasks was assigned to the subsequent phase to capture proximal influence on the phase (pre-onset window: manipulation phase: *M* = 4.7 min; free interaction phase: *M* = 1.0 min). Relative laughter duration was then computed as the proportion of laughter within this phase-specific window, including the pre-onset window. Specifically, the total laughter duration (in seconds) was divided by the exact length of the respective phase window to account for differences in window lengths across phases. This relative laughter duration served as the laughter behavior variable in the analyses for two distinct time points.

**TABLE 1:** Descriptive statistics (means and standard deviations per dyad) of the laughter duration per experimental phase in seconds during the manipulation phase and the subsequent free interaction phase, presented separately for the four experimental groups defined by Laughter (laughter vs. no laughter) and Social context (interaction vs. no interaction).

Conditions	Laughter behavior during manipulation phase (in seconds)	Laughter behavior during free interaction phase (in seconds)
Laughter; social context	*M* = 135.00, *SD* = 82.20	*M* = 51.20, *SD* = 36.60
Laughter; no social context	*M* = 51.20, *SD* = 60.20	*M* = 47.60, *SD* = 26.40
No laughter; social context	*M* = 36.10, *SD* = 26.20	*M* = 50.20, *SD* = 36.20
No laughter; no social context	*M* = 0.52, *SD* = 1.28	*M* = 50.40, *SD* = 27.70

#### 2.2.2 fNIRS hyperscanning

Neural data were assessed using fNIRS-hyperscanning with NIRSport 1 devices (NIRx Medizintechnik GmbH, Germany) and a sampling rate of 7.81 Hz. fNIRS is a non-invasive neuroimaging method that uses near-infrared light to measure changes in the concentration of oxygenated and deoxygenated hemoglobin, providing insights into neural activity (Lloyd-Fox et al., 2010). It is widely used in social cognition research due to its flexibility and tolerance for motion, allowing measurements in naturalistic settings (Alonso et al., 2024; Nguyen et al., 2021; Yücel et al., 2017).

The fNIRS optodes were positioned over selected regions of interest (ROIs): the bilateral IFG (Gvirts and Perlmutter, 2020; Nummenmaa et al., 2023; Shamay-Tsoory et al., 2019) and bilateral TPJ (Gvirts and Perlmutter, 2020; Shamay-Tsoory et al., 2019). For each participant, 16 channels were used to estimate brain activity over these areas bilaterally: the TPJ (4 channels per side, centered on positions CP5 and CP6 of the 10–20 system) and the IFG (4 channels per side, centered on positions F7 and F8). Due to the spatial resolution of fNIRS, which is not as high as fMRI (Lloyd-Fox et al., 2010), our optode placement over the TPJ also captures activity from the posterior part of the superior temporal gyrus (STG). For simplicity, we will refer to this region as the TPJ throughout the paper.

fNIRS data were preprocessed in MATLAB (Version 2022a) using functions from the Homer2 software package (Huppert et al., 2009). Preprocessing steps included conversion of raw wavelength data to optical density, motion correction, and filtering to remove physiological noise and artifacts (Lloyd-Fox et al., 2010). Specifically, motion artifacts were addressed using spline interpolation and wavelet filtering, based on the assumption that frequent jaw movements would be a primary source of noise in the signal (Novi et al., 2020). The optical density data were then transformed into concentration changes in oxygenated (HbO) and deoxygenated hemoglobin (HbR) (Lloyd-Fox et al., 2010). For further analysis, we used HbO data because HbO signals respond more clearly to variations in cerebral blood flow (Hoshi, 2016) and offer a better contrast-to-noise ratio than HbR (Strangman et al., 2002), making HbO data the most reliable fNIRS measure (for results on HbR data, see Supplementary material). After the preprocessing, we needed to exclude two (manipulation phase) and seven (free interaction phase) dyads because of bad data quality, resulting in a sample size of 96 dyads (synchrony during manipulation) and 91 dyads (synchrony during free interaction) for the analysis with neural data.

#### 2.2.3 Interpersonal neural synchrony

The analysis of neural synchrony was guided by the methodology outlined in Nguyen et al. (2021). Interpersonal synchrony was analyzed during two phases of the experiment: first, during the video-watching phase of the manipulation in the experiment, and second, during the free conversation. Neural synchrony was quantified using Wavelet Transform Coherence (WTC), a widely used method for examining coherence between two time-series across both frequency and time domains (Cui et al., 2012; Grinsted et al., 2004; Nguyen et al., 2021). WTC provides a powerful tool for exploring the dynamic alignment of neural activity during complex social interactions (Cui et al., 2012; Nguyen et al., 2021). We set the WTC period range adaptively per segment: from 8 s up to one quarter of the segment duration, ensuring multiple cycles of the slowest fluctuations. Cone-of-influence edges were excluded before averaging (Nguyen et al., 2021). For example, in a 5-min segment (300 s), the longest allowed period is 300 divided by 4, i.e., 75 s; periods shorter than 8 s are excluded, so the analyzed range is 8–75 s (approximately 0.0133–0.125 Hz). Based on four predefined regions of interest (ROIs), left and right inferior frontal gyrus (IFGl, IFGr) and left and right temporoparietal junction (TPJl, TPJr); we computed WTC for all possible pairwise combinations across hemispheres and regions for the two phases of synchrony measurements. This resulted in 10 ROI pairs: IFGl_IFGl, IFGr_IFGr, TPJl_TPJl, TPJr_TPJr, IFGl_IFGr, IFGl_TPJl, IFGl_TPJr, IFGr_TPJl, IFGr_TPJr, and TPJl_TPJr. WTC values were then averaged across all available frequencies (period range 8–75 s) and time points, resulting in one coherence value per ROI pair per dyad for each experimental phase.

#### 2.2.4 Positive outcome variables

##### 2.2.4.1 Liking questions

To assess liking between the participants, we used three liking questions adapted from Lakin and Chartrand (2003). Participants first completed the questions at the very beginning of the session, before any interaction with their partner (Liking 1), and again after the free conversation part (Liking 2). In both instances, participants rated (1) how comfortable they felt with the other participant, (2) how likable they perceived the other participant to be, and (3) whether they would like to spend more time with the other participant. All responses were recorded on a 9-point Likert scale ranging from 0 (“does not apply at all”) to 8 (“fully applies”).

##### 2.2.4.2 Inclusion of Other in the Self Scale

Bonding was measured between participants using the Inclusion of Other in the Self Scale (IOS) (Aron et al., 1992). This measure presents seven diagrams, each with two circles labeled “Self” and “Other” with increasing degrees of overlap. Participants were asked to select the diagram that best described how close they felt to the other participant. The diagrams were numbered from 1 (no overlap) to 7 (almost complete overlap), and responses were coded accordingly as a 7-point Likert scale, with higher values indicating greater perceived closeness.

##### 2.2.4.3 Prosociality questions

Prosocial intentions were measured using four custom items developed for this study. Participants indicated (1) how much money they would spend on a birthday present for the other participant (0–30 €), (2) how much money they would typically spend on a birthday present for their friends (0–30 €), (3) how likely they would be to help the other participant move, and (4) how likely they are to help someone move in general. The latter two questions were rated on a 4-point Likert scale ranging from 1 (“very unlikely”) to 4 (“very likely”). The two items concerning friends and general helping behavior served as baseline measures of general prosocial tendencies, allowing for comparison with responses directed toward the other participant.

### 2.3 Experimental procedure

#### 2.3.1 Procedure

**FIGURE 1:**
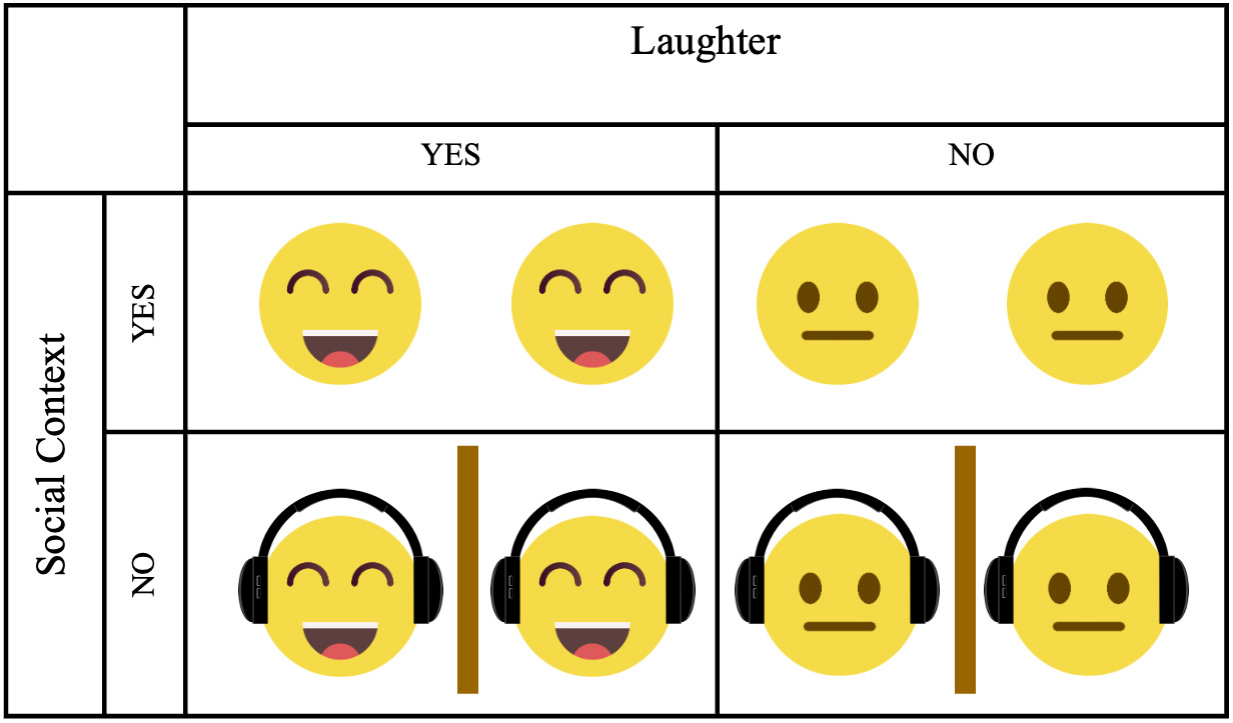
Illustration of the 2 × 2 experimental design with the factors *Laughter* (yes vs. no) and *Social context* (yes vs. no), resulting in four conditions. In the upper row, participants interacted directly and either watched funny videos together (Laughter + Social) or neutral videos together (No Laughter + Social). In the lower row, participants did not interact, they were visually and acoustically separated using a physical barrier and headphones, and were exposed to either funny (Laughter + No Social) or neutral (No Laughter + No Social) videos. Emojis and icons were adapted from Canva.

We structured the experimental procedure into three consecutive phases: a manipulation phase, a free conversation phase, and an outcome phase. Participants took part in the study in same-gender dyads. We randomly assigned each dyad to one of four conditions in a 2 (Laughter: yes vs. no) × 2 (Social context: yes vs. no) between-subjects design (Figure 1). While the manipulation phase varied by condition, the free interaction and outcome phases followed the same procedure for all participants.

#### 2.3.2 Initial setup

When participants arrived at the lab, we seated them face-to-face at a 1 m × 1 m table. After a brief welcome, we applied fNIRS caps to both participants. To prevent visual contact, we then placed a visual barrier (“separator”), a wooden panel positioned at the center of the table, between them. After setting up the separator, we asked participants to complete the initial liking questions (Liking 1).

#### 2.3.3 Manipulation phase

The manipulation phase included two components: a modified Mad Libs game and the viewing of animal video clips. All participants completed both tasks, but the degree of social interaction and the humorous content varied depending on the condition. In the Social conditions, we removed the separator after the initial questionnaire and allowed participants to see and hear each other during the manipulation phase. In contrast, in the Non-Social conditions, we kept the separator in place and had participants wear headphones throughout the manipulation phase to prevent both visual and auditory contact.

We instructed all participants to complete two rounds of a modified Mad Libs game. In each round, participants received a story with missing words along with a list of word options. In the Social conditions, they played the game collaboratively: one participant selected a word aloud, the other inserted it into the story, and then read the completed story aloud. In the Non-Social conditions, participants completed the task individually and silently. They selected words from the list on their own before reading the text, and subsequently filled in the blanks with those words and read the story to themselves.

We varied the content of the word lists depending on the laughter manipulation (Table 2). In the Laughter conditions, the materials included funny and absurd options intended to elicit laughter, and a soundtrack of recorded natural laughter to assure that both participants heard a minimum amount of laughter at the same time even in the Non-Social Laughter condition. In the Control conditions, we used neutral content with neutral sounds, for example, from wind and water, as well as animals moving through grass.

**TABLE 2:** Example text from the stimuli material for the mad lips game.

Condition	Example text	Example words
Funny context	Getting ready for your new baby can make you feel (1)_________ (emotion) and (2)_________ (emotion). Here are some (3)________ (adjective) tips to help you prepare.	(1) Embarrassed, drunk, sleepy … (2) Revengeful, murderous … (3) Unhelpful, pervert …
Neutral context	One (6)__________________ (adjective) option is (7) ____________ (number) second videos that can be watched on a smartphone or tablet without much effort.	(6) Practical, great, easy … (7) 10, 20, 30 …

Participants received either humorous or neutral texts depending on their assignment to the Laughter or Non-Laughter condition. The same texts were used across the Social and Non-Social context conditions. In the Social context condition, participants exchanged missing words with a partner and read the completed stories aloud. In the Non-Social context condition, participants filled in the missing words by themselves and read the stories silently.

After the game, we presented two five-minute video clips featuring animal footage on a screen placed approximately 170 cm in front of each participant. In the Laughter conditions, the videos contained humorous animal content and recorded natural laughter (e.g., animals slipping, reacting unexpectedly, or interacting playfully), compiled from publicly available online sources. In the Control conditions, the clips were neutral and contained ambient sounds. In the Non-Social context conditions, participants watched the videos while remaining separated and wearing headphones. In the Social conditions, they watched the videos together without any physical or auditory barriers. Furthermore, to increase the interactive aspect of the Social conditions, participants had to rate the video quality together after each video, whereas in the Non-Social conditions they had to rate it on their own. Additionally, during the video watching we recorded the brain activity of the two participants with fNIRS.

#### 2.3.4 Free conversation phase

After the video presentation, we removed the headphones and the separator in all conditions. At this point, the experimenter informed participants that the next task needed preparation and left the room. We left the participants alone in the room for 10 min without further instruction to allow spontaneous and unstructured conversation. During this phase, we continuously recorded neural synchrony via fNIRS.

#### 2.3.5 Outcome phase

**FIGURE 2:**
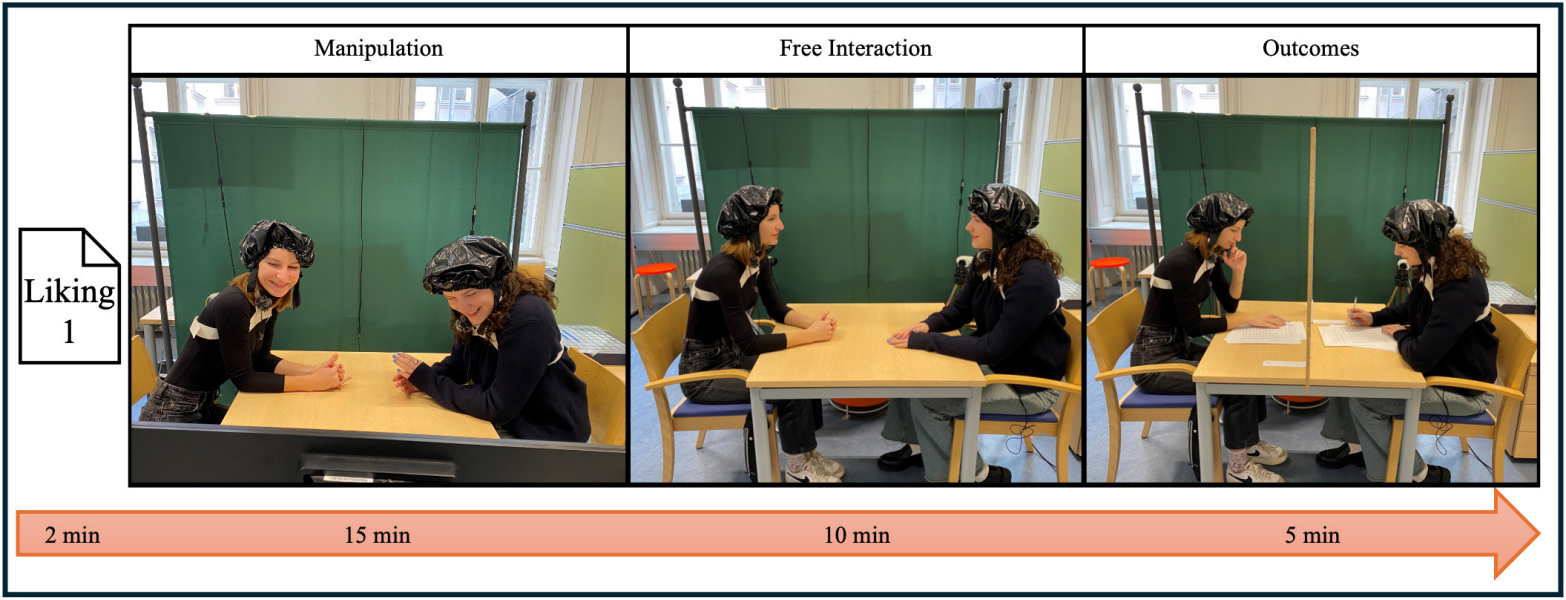
Experimental setup during the Laughter Social condition. Two participants are seated face-to-face at a table, each wearing a fNIRS cap. Liking 1 (pre-test): questionnaire assessment. Manipulation: during which participants watched funny animal videos while facing a screen. Free Interaction: involving a 10-min free conversation between the dyad. Outcomes: where participants were separated by a wooden panel and independently completed questionnaires assessing Liking 2, Prosociality, and Bonding. The pink arrow represents the progression of time across these experimental phases.

After the free conversation phase, we returned to the room and placed the separator back between participants. We then asked them to complete the second liking questions (Liking 2), the IOS, and the prosociality questions. Following the social questions, participants completed an additional task that is not part of the present analyses and will therefore not be discussed further. Finally, we debriefed participants and provided their compensations. The entire session lasted approximately 90 min. An example timeline of the Laughter Social condition is presented in Figure 2.

### 2.4 Statistical analysis

#### 2.4.1 Manipulation check

To check if the participants laughed more in the Laughter conditions compared to the Non-Laughter conditions, we conducted a two-factor Bayesian ANOVA. As the dependent variable served the amount of laughter from the annotated laughter behavior. In addition, we conducted another Bayesian ANOVA to check if the laughter behavior was different during the free conversation phase.

Neural data preprocessing and the computation of WTC values were carried out in MATLAB (Version R2022a), using custom scripts and signal processing toolboxes. All further statistical analyses were performed in RStudio (Version 2024.04.2). All statistical analyses were conducted using Bayesian methods. The general modeling workflow for ANOVAs and hierarchical models followed the structure proposed by Nicenboim et al. (2025) using the brms package (Bürkner, 2017). Bayesian mediation analyses were conducted using the implementation provided by the bayestestR package (Makowski et al., 2019).

#### 2.4.2 Analysis of neural synchrony

We analyzed neural synchrony during the manipulation phase by dividing it into two time intervals, corresponding to the two 5-min video segments. Additionally, we analyzed neural synchrony during the free conversation phase, splitting the 10 min segments into two 5 min segments in order to assess changes in synchrony during the conversation. First of all, to determine the presence of neural synchrony between experimental dyads, surrogate data were generated following a random permutation procedure adapted from Nguyen et al. (2021): first, we calculated WTC values between each participant and 100 other randomly selected participants. Then, we averaged all 100 WTC matrices for each participant, thus creating a full sample of pseudo-dyads. This procedure was conducted separately for the two phases of synchrony measurement. Bayesian ANOVAs were then conducted for each ROI pair to compare synchrony between real and surrogate dyads.

#### 2.4.3 Hypotheses 1: Laughter enhances neural synchrony

**FIGURE 3:**
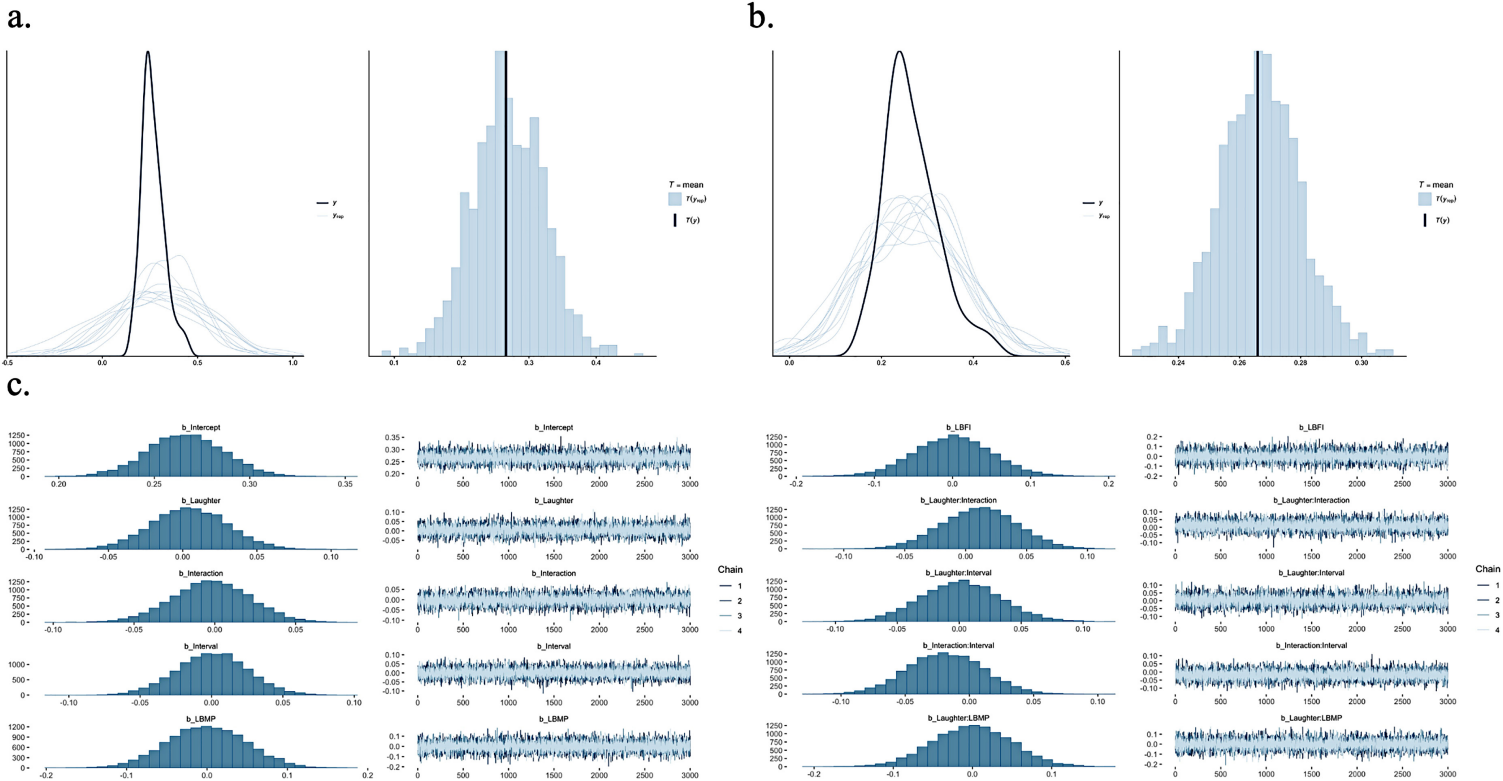
Bayesian workflow example for the ROI pair IFGr_IFGr during the free interaction phase. **(a)** Prior predictive checks before model fitting. The density overlay compares prior predictive draws (light lines) with the observed data distribution (bold line), followed by a comparison of the mean statistic. **(b)** The posterior predictive checks comparing the model’s predictions to the observed data, both in terms of the full distribution (density overlay) and the mean as a summary statistic. **(c)** Diagnostics and model evaluation after fitting the final model. The left column displays the posterior distributions of all model parameters, while the right column shows their respective trace plots across four chains, indicating convergence. Laughter Behavior during the Manipulation phase (LBMP) and Laughter Behavior during the Free Interaction phase (LBFI). Only example plots for individual parameters are shown here (for all plots of the remaining parameters, see Supplementary material).

To test the hypothesis that Laughter enhances Neural synchrony between individuals, we conducted Bayesian analyses separately for each ROI-pair with a mixed design. These analyses were performed separately for each of the two phases of synchrony measurement. Sample sizes for the different ROI pairs for synchrony during manipulation range between 68 (TPJl_TPJl) and 96 (IFGr_IFGl), and for synchrony during free interaction range between 60 (TPJr_TPJr) and 91dyads (IFGl_TPJr) (for detailed sample size description see Supplementary material). Neural synchrony served as the dependent variable in the subsequent models, operationalized as WTC values. The fixed effects included the between-subject factors Laughter Manipulation (Laughter vs. Control) and Social Context (Social vs. Non-Social), as well as the within-subject factor Interval (first vs. second half of the conversation). Additionally, for synchrony during the manipulation phase, the fixed effects included the continuous variable Laughter Behavior during the Manipulation phase (LBMP), which was derived from the laughter annotations. For synchrony during the free interaction phase, the fixed effects included both LBMP and Laughter Behavior during the Free Interaction phase (LBFI), as the latter was measured after the synchrony assessment during the manipulation phase. All possible interactions among the fixed effects were modeled: SynchronyROI∼Laughter Manipulation*Social*Interval*LBMP*LBFI (only for synchrony analyses during the free interaction phase). We started the Bayesian analyses by conducting prior analyses. The prior for the intercept was informed by the mean synchrony values observed in pilot data, while a weakly informative standard deviation was chosen to reflect general uncertainty. For the regression coefficients (beta) and the residual standard deviation (sigma), we opted for noninformative priors. Prior predictive checks were carried out using the brms package (Bürkner, 2017), following recommendations from Nicenboim et al. (2025), to ensure that the priors led to plausible predictions. All models were specified using the Gaussian family. Following the prior checks, we proceeded with model fitting for parameter estimation. Posterior distributions were estimated via Markov Chain Monte Carlo (MCMC) sampling as implemented in brms. Model convergence was assessed using R-hat statistics, trace plots, and posterior predictive checks (for an example workflow, see Figure 3). Once convergence was confirmed, we examined the posterior distributions of the model parameters to evaluate support for our hypotheses.

In addition to parameter estimation, we performed Bayesian model comparisons using Bayes factors (Wagenmakers et al., 2018; Nicenboim et al., 2025) for each ROI pair. For Hypothesis 1a (synchrony is increased by Laughter irrespective of social context), which focuses on the main effect of Laughter, the full model was compared to different null models. For synchrony during the manipulation phase, the full model was compared to two null models: (1) a model omitting the Laughter Manipulation factor entirely, (2) a model omitting the Laughter Behavior during the Manipulation phase. For synchrony during the free interaction phase, an additional comparison was included: the full model was compared to a third null model without the Laughter Behavior during the Free Interaction phase. For Hypothesis 1b (Laughter in a social context increases synchrony), which focuses on the interaction effect between Laughter (Manipulation, LBMP, and LBFI (only for synchrony analysis during the free interaction phase) and Social, the full model was compared to a reduced model excluding the key interaction term (synchrony during manipulation ∼ Laughter Manipulation + Social + Laughter Manipulation: Interval + Social: Interval + LBMP + LBMP: Interval and synchrony during free interaction ∼ Laughter Manipulation + Social + Laughter Manipulation:Interval + Social:Interval + LBMP + LBMP: Interval + LBFI +LBFI: Interval).

#### 2.4.4 Hypothesis 2: Laughter enhances social outcomes

To test whether Laughter alone or in a social context enhances social outcomes, we analyzed three outcome variables: Liking, Bonding, and Prosocial Behavior. Each participant completed the measures individually, resulting in two data points per dyad. To account for this non-independence of observations within dyads, a hierarchical term (random intercept for dyad) was included in all models. All outcome variables were analyzed using Bayesian hierarchical models with the between-subject factors Laughter Manipulation (Laughter vs. Control) and Social Context (Social vs. Non-Social), and the two timepoints of the Laughter Behavior variables (LBMP and LBFI).

For the *Liking* outcome, participants responded to three items both before and after the manipulation and the conversation phase. To examine whether the three items assessing Liking could be combined into composite scores, we calculated Cronbach’s alpha between all item pairs for both time points (L1 and L2). For Liking 1, the internal consistency was good, with α = 0.80, 95% CI (0.75, 0.85). For Liking 2, internal consistency was similarly high, with α = 0.82, 95% CI (0.77, 0.86). These results indicated good internal consistency at each time point, so the items were averaged into one composite score before (Liking 1) and one after the interaction (Liking 2). A similar approach of averaging Liking scores at the dyadic level was used in previous work (Ravreby et al., 2022). In the Bayesian model, Liking 2 served as the dependent variable, while Liking 1 was grand-mean centered (Nicenboim et al., 2025) and included as a covariate to account for baseline liking.

*Prosocial behavior* was derived from four questions. First, in the Birthday Money Task, we calculated the percentage of how much money participants would give to the other person, relative to how much they would spend on a friend. Second, in the Helping Task, we also calculated the percentage of helping willingness directed toward the other participant and general helping willingness. To assess whether the two items could be combined, we calculated Cronbach’s alpha [α = 0.45, 95% CI (0.28, 0.59)] which revealed no convincing evidence. Therefore, we computed separate analyses for the two Prosociality variables.

*Bonding* was measured using a single-item IOS scale and directly entered as the dependent variable. The sample size consists of 195 participants because we needed to exclude one participant due to an inconclusive answer in the questionnaire. The same Bayesian workflow was applied for all three outcome variables, as described for the Analyses of Hypothesis 1: prior specification, parameter estimation via MCMC sampling, and model comparison using Bayes factors for both hypotheses (H2a and H2b). For H2a (Laughter increases the outcome variable (Liking 2, Prosociality or Bonding) irrespective of a social context) we compared the full model (outcome variable ∼ Laughter Manipulation + Social + LBMP + LBFI + (1| Dyad)), to three null models: (1) a model omitting the Laughter Manipulation factor entirely, (2) a model omitting LBMP, and (3) a model omitting LBFI. For hypothesis 2b (laughing together increases the outcome variables), we compared the full model [outcome variable ∼ Laughter Manipulation * Social * LBMP * LBFI + (1| Dyad)] with the null model [outcome variable ∼ Laughter Manipulation + Social + LBMP + LBFI + (1| Dyad)].

#### 2.4.5 Hypothesis 3: neural synchrony mediates the link between Laughter and social outcomes

To test whether neural synchrony mediated the effects of Laughter and Social Context on social outcomes, we conducted separate Bayesian mediation analyses for each Laughter variable (Laughter Manipulation, Laughter Behavior during the Manipulation, and Laughter Behavior during the Free Interaction), each outcome variable (Liking, Prosociality, Bonding), and each of the ten ROI pairs for the two measurement timepoints of synchrony.

We prepared a dyad-level dataset combining neural synchrony values and aggregated social outcome measures. Synchrony values were averaged across both intervals of either the video watching or the interaction period for each dyad. Social outcomes (Liking 2, Prosociality, Bonding) were each summed across both dyad members. The resulting dataset contained one row per dyad, with mean neural synchrony values for each ROI pair and the corresponding aggregated outcome scores. This dataset served as the input for the mediation models. In each model, Laughter (Manipulation: Laughter vs. Control, LBMP (only for synchrony analysis during manipulation phase) or LBFI) and Social context (Social vs. Non-social) were entered as predictors, neural synchrony (WTC) for a specific ROI pair and timepoint of measurement served as the mediator, and the respective social outcome as the dependent variable. All mediation models were estimated using the Bayesian estimation framework implemented in brms and bayestestR (Makowski et al., 2019).

## 3 Results

Throughout, we report posterior means and 95% credible intervals (CI). The analyses of the proportion of time spent laughing (in seconds) per condition reveal that the effect of including the Laughter manipulation has a positive association with the actual amount of laughter [Laughter Manipulation: estimate = 34.96, 95% CI (10.4, 59.88); Laughter Manipulation *Social: estimate = 44.28, 95% CI (16.97, 71.09)] compared to including the effect of the Social context alone [estimate = 23.84, 95% CI (−0.33, 47.75)]. This shows that participants laughed more during the manipulation phase in the Laughter conditions than in the Control conditions. Therefore, in the following analysis, the comparisons between conditions will reflect the actual laughter behavior of the participants. Furthermore, we checked whether the amount of laughter during the free conversation part was different between the groups. The results show that there were no differences in the laughter behavior between the Laughter Manipulation effect [estimate = −0.73, 95% CI (−12.27, 10.81)]; the interaction between Laughter Manipulation and Social context [estimate = 2.71, 95% CI (−11.11, 16.53)] and the effect of Social context alone [estimate: 1.62, 95% CI (−9.75, 13.24)]. These results show that after the manipulation phase, the laughter behavior of the participants did not differ between the experimental groups during the free conversation phase.

**FIGURE 4:**
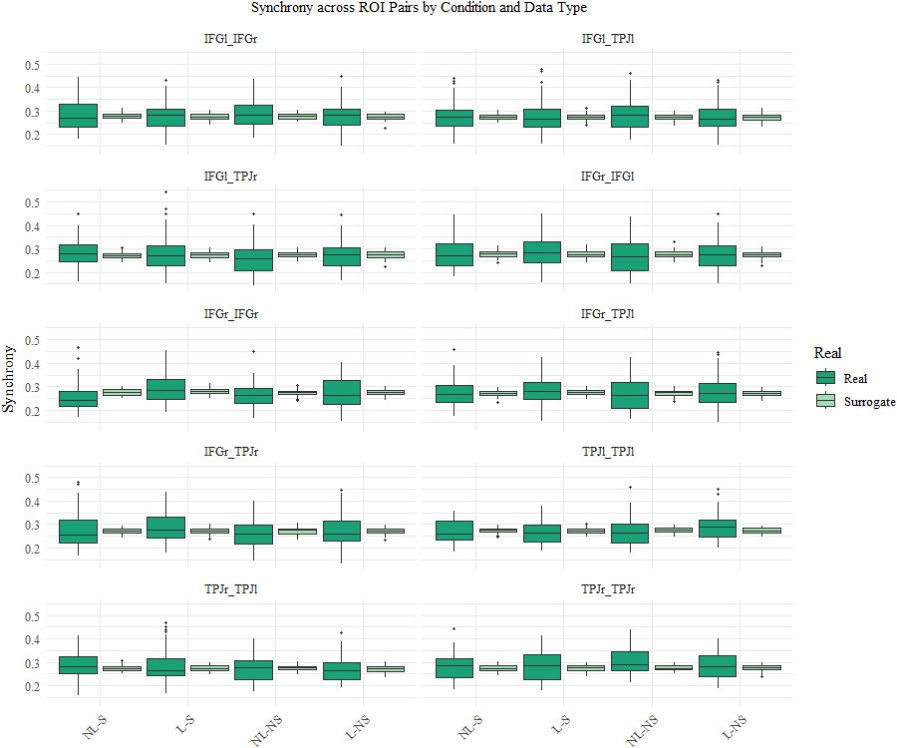
Boxplots of the surrogate data analyses for synchrony measured during the free interaction phase. In dark green, the boxplots for the real data and in light green boxplots for the surrogate data across the four conditions: No Laughter but Social (NL-S), Laughter and Social (L-S), No Laughter and No Social (NL-NS) and Laughter but No Social (L-NS).

The surrogate analysis revealed no difference between the real and the surrogate pairs (for all results, see Supplementary Tables 1, 2). Notably and surprisingly, this suggests that the overall level of neural synchrony we observed in the real dyads does not exceed the levels of synchronization that might be expected by chance or due to the measurement environment and procedures per se. Nevertheless, we observed greater variability among real participant pairs (see Figure 4), indicating that differences in synchrony across testing sessions may still emerge depending on the condition. Furthermore, we were still interested in potential condition effects in the real dyads. Therefore, we decided to include all ROI pairs in our analyses.

### 3.1 Results for hypothesis 1: no effect of Laughter on neural synchrony

Bayesian models were fitted separately for each ROI pair and experimental phase to test whether a shared laughter experience enhances neural synchrony. Throughout, we report posterior means and 95% credible intervals (CI). Across all ROI pairs, posterior estimates for the Interaction-model [Laughter Manipulation * Social * Interval * LBMP (* LBFI)] were close to zero, with all 95% CIs overlapping zero for both measurement time points. This suggests no convincing evidence for an interaction effect of Laughter Manipulation, Laughter Behavior, Social context, and Time on neural synchrony (for full estimates, see Supplementary Tables 3, 4). Overall, the parameter estimation does not provide clear evidence for an increase in neural synchrony as a function of Laughter, either alone or in interaction with Social context and Time. To complement the parameter estimation, Bayes factors were computed to evaluate whether the Laughter Manipulation in general or the Laughter Behavior has an effect on synchrony (H1a) or whether a shared laughter experience increases neural synchrony (H1b). For all ROI pairs, Bayes factors were below 1, indicating that the data were more likely under the simpler models without Laughter-related predictors. Bayes factors for H1a ranged from *BF10* = 0–0.96, and for H1b from *BF10* = 0.00–0.40, consistently favoring the null models (see Supplementary Tables 5, 6). Taken together, these results provide moderate to strong evidence for the null hypotheses, suggesting that neither the Laughter Manipulation nor Laughter Behavior influenced neural synchrony in the current study.

### 3.2 Results for hypotheses 2: model-comparison evidence for Laughter effects on Liking, Money Sharing and Bonding, exploratory support for initial liking and interaction

To examine whether participating in a shared laughter experience impacts social outcomes at a later time point, we analyzed three dependent variables: Liking, Prosociality, and Bonding. Each of these outcomes was modeled using a Bayesian hierarchical approach, including the predictors Laughter Manipulation, Laughter Behavior (during manipulation and during free interaction phase), Social context, and their interaction. Random intercepts for dyads were included to account for the nested structure of the data.

The following results are based on the model predicting Liking after the interaction phase, with Liking 1 (first impression Liking) included as a covariate. On average, participants reported moderately positive levels of Liking both before (*M* = 5.88, *SD* = 1.15) and after the interaction (*M* = 6.26, *SD* = 1.16). The posterior estimates for the Laughter Manipulation [estimate = −0.03, 95% CI (−0.33, 0.26)], Laughter Behavior [during manipulation: estimate = 0.00, 95% CI (−0.59, 0.6) and during free interaction: estimate = −0.00, 95% CI (−0.58, 0.59)] and Social context [estimate = −0.07, 95% CI (−0.36, 0.21)] were centered near zero, and their credible intervals included zero. The interactions between the variables were also all close to zero (Supplementary Table 7). In contrast, Liking 1 showed a strong positive association with Liking 2 (estimate = 0.70, 95% CI (0.60, 0.81)]. To follow up on this finding, we conducted an exploratory model comparison testing the added value of including Liking 1. This comparison yielded *BF10* > 100, indicating extreme evidence in favor of including this covariate. These results suggest that first impressions were the primary driver of participants’ post-interaction liking.

For model comparison with Bayes factors, the full model for hypothesis 2a (Laughter increases Liking 2 irrespective of a Social context), which included all laughter-related variables (Laughter Manipulation, Laughter Behavior during the Manipulation phase, and Laughter Behavior during the Free Interaction phase) was compared to the null model omitting the Laughter Manipulation variable. The Bayes factor for this comparison was BF10 = 0.51, indicating anecdotal evidence in favor of the null model. However, when comparing the full model to a model omitting only LBMP, the Bayes factor was BF10 > 100, providing decisive evidence that including LBMP improves the prediction of Liking 2. Similarly, the comparison of the full model to a model omitting LBFI also yielded a Bayes factor of BF10 > 100, indicating very strong evidence that including LBFI leads to a better model fit. These results suggest that the Laughter Behavior during both phases of the experiment is a critical predictor of Liking 2. The model comparison for the hypotheses H2b (laughing together increases Liking 2) revealed a Bayes factor of *BF10* = 0.76, which means we found anecdotal evidence in favor of the null model (Supplementary Table 8).

We next examined Prosociality, as assessed through participants’ decisions in the Helping (*M* = 0.86, SD = 0.22) and Money-giving questions (*M* = 0.61, *SD* = 0.25). The analysis of Money-giving resulted in small posterior estimates for Laughter Manipulation [estimate = −0.08, 95% CI (−0.19, 0.03)], Laughter Behavior [during manipulation: estimate = −0.00, 95% CI (−0.59, 0.58) and during free interaction: estimate = 0.00, 95% CI (−0.57, 0.58)] and Social context [estimate = 0.03, 95% CI (−0.08, 0.13)]. The interactions between all predictors were also small and included zero (Supplementary Table 7). The Bayes factor for the model comparison of hypothesis 2a was *BF10* = 0.34, when comparing the full model with the null model omitting the Laughter Manipulation variable, indicating moderate evidence in favor of the null model. However, when comparing the full model to a model without LBMP, the Bayes factor was *BF10* = 23.25, and for the comparison with a model lacking LBFI, the Bayes factor was *BF10* = 23.84, both providing strong evidence that including these Laughter Behavior variables improves the prediction of Money Sharing. For hypothesis 2b (laughing together increases money sharing), the comparison of the full model, which included interactions between Laughter (Manipulation, LBMP, and LBFI) and Social Context, with the null model yielded a Bayes factor of *BF10* = 0.32, indicating moderate evidence in favor of the null model (Supplementary Table 8). The parameter estimation for the helping questions also revealed small posterior estimates for Laughter Manipulation [estimate = −0.01, 95% CI (−0.11, 0.09)], Laughter Behavior [during manipulation: estimate = 0.00, 95% CI (−0.58, 0.59) and during free interaction: estimate = 0.00, 95% CI (−0.57, 0.59)], Social context [estimate = 0.07, 95% CI (−0.03, 0.16)] and the interactions between all variables again where all small and close to zero (Supplementary Table 7). For the model comparison of hypothesis 2a, the Bayes factor for the comparison of the full model to a model excluding Laughter Manipulation was *BF10* = 0.13, indicating moderate evidence in favor of the null model. When comparing the full model to a model excluding Laughter Behavior during the Manipulation phase, the Bayes factor was *BF10* = 0.01, providing strong evidence in favor of the null model. Similarly, the comparison of the full model to a model excluding Laughter Behavior during the Free Interaction phase yielded a Bayes factor of *BF10* = 0.61, indicating anecdotal evidence in favor of the null model. For hypothesis 2b, the model comparison resulted in a Bayes factor of *BF10* = 0.24, indicating moderate evidence in favor of the simpler model (Supplementary Table 8). In total, these results suggest that Prosocial Behavior, regardless of whether it was measured by Helping Behavior or Money Sharing, was not reliably influenced by Laughter manipulation or Social context. However, for Money Sharing, the Bayes factor comparisons indicated that the inclusion of the two Laughter Behavior variables improved model fit.

Finally, we examined Bonding, measured via IOS (*M* = 2.63, *SD* = 1.02). The estimates for Laughter manipulation [estimate = −0.05, 95% CI (−0.38, 0.29)], Laughter Behavior [during manipulation: estimate = −0.00, 95% CI (−0.59, 0.59) and during free interaction: estimate = −0.00, 95% CI (−0.59, 0.58)], for Social context [estimate = 0.28, 95% CI (−0.05, 0.60)] and the interactions between the Laughter variables and Social context were small, and their credible intervals included zero (Supplementary Table 7). The Bayes factor for the model comparison of hypotheses H2a versus a null model without the Laughter Manipulation variable was *BF10* = 0.79, indicating anecdotal evidence for the null hypothesis. However, comparisons of the full model to models excluding Laughter Behavior during the Manipulation phase and during the Free Interaction phase revealed extremely strong evidence for the inclusion of these variables, with both Bayes factors > 100. For hypothesis 2b, the model comparison revealed a Bayes factor of *BF10* = 1.35, suggesting that the data were equally likely under both models (Supplementary Table 8).

### 3.3 Results for hypothesis 3: no evidence for mediation, yet both synchrony in specific ROI pairs and Laughter Behavior predict Liking and Bonding

To explore whether neural synchrony mediated the relationship between a Shared Laughter Experience and social outcomes, we conducted Bayesian mediation analyses using the bayestestR package. Separate models were computed for each Laughter variable (Manipulation, Laughter Behavior during manipulation and during free interaction phase) and each ROI pair for each of the two experimental phases and each outcome variable [Liking 2, Bonding, Money giving (Prosociality), and Helping (Prosociality)]. The Laughter variables each served as the predictor (treatment), the respective synchrony measure as the mediator, and the social outcome as the dependent variable. Posterior distributions were estimated for the average causal mediation effect (indirect effect), the average direct effect, and the total effect.

We report the posterior estimates along with their 95% Equal-Tailed Interval (ETI), as recommended for Bayesian mediation (Makowski et al., 2019), we interpret indirect effects based on whether their ETI includes zero and, where applicable, supplement these results with Bayes factors (*BF10*) to quantify the relative evidence in favor of models with or without the mediator. Detailed results for all path estimates can be found in Supplementary Tables 9, 10. In the following sections we will report the results presented for each outcome separately, beginning with Liking.

**FIGURE 5:**
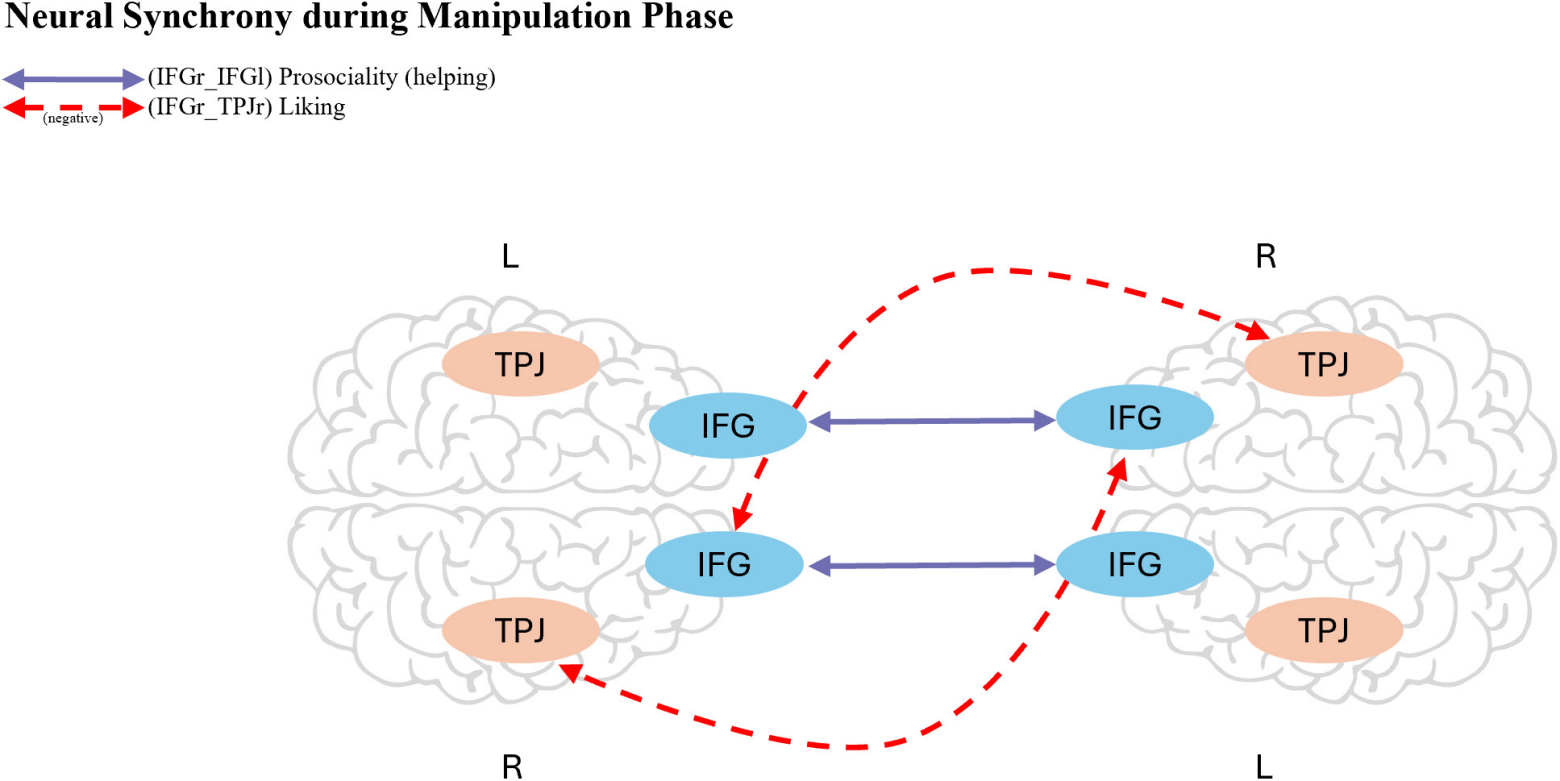
Schematic illustration of the two ROI pairs, for synchrony during the manipulation phase, that showed strong evidence for associations with social outcomes in the exploratory analyses for hypothesis 3. Synchrony between the left and right inferior frontal gyrus (IFG) was associated with higher Helping ratings (*BF*10 = 34.13, *BF*10 = 41.31), while synchrony between the right IFG and the right temporoparietal junction (TPJ) was negatively associated with Liking (*BF*10 > 100). Arrows indicate the corresponding ROI connections across hemispheres, and dashed lines indicate a negative association. The brain template was adapted from Canva.

**FIGURE 6:**
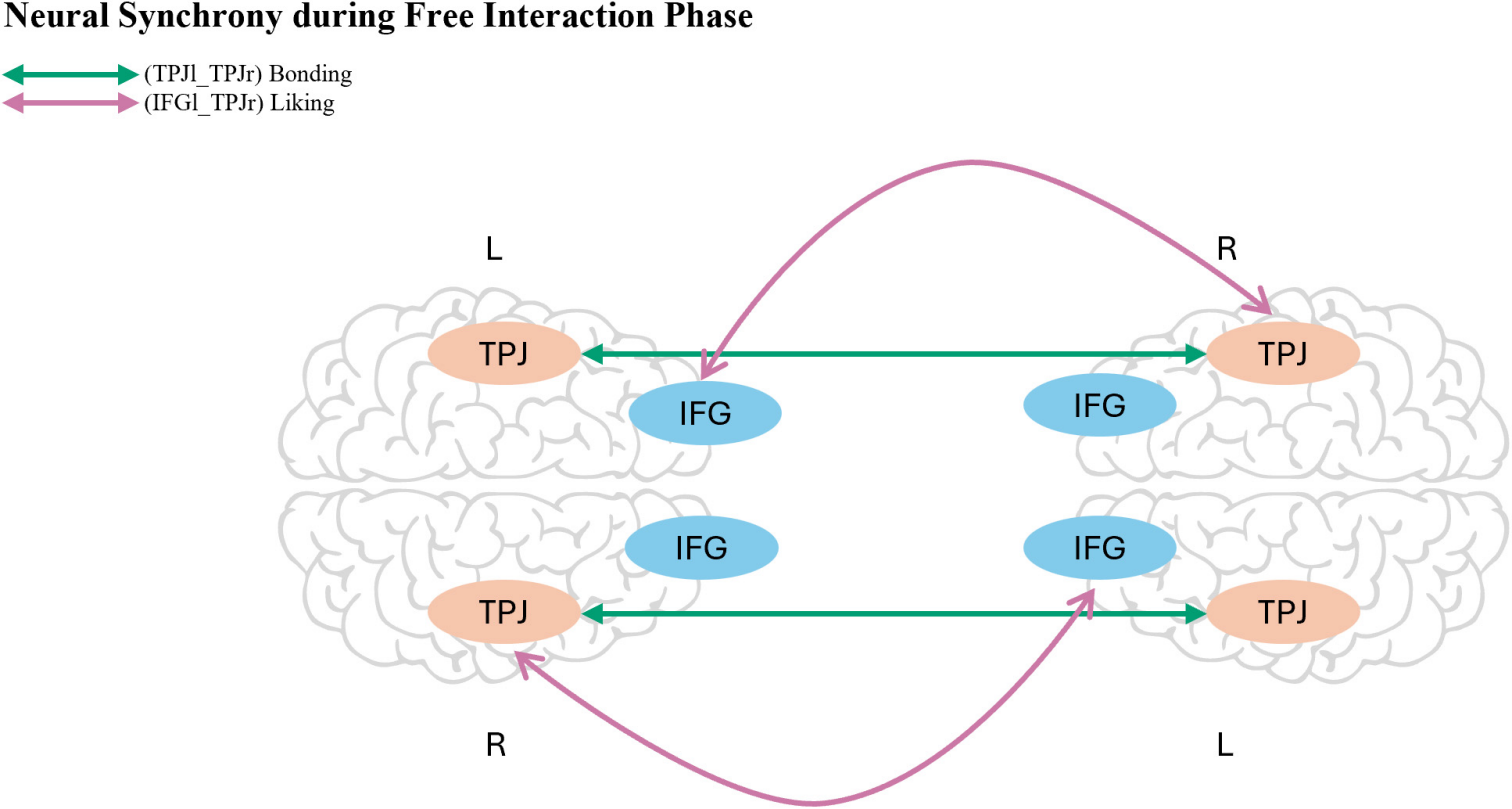
Schematic illustration of the two ROI pairs, for synchrony during the free interaction phase, that showed strong evidence for associations with social outcomes in the exploratory analyses for hypothesis 3. Synchrony between the left inferior frontal gyrus (IFG) and the right temporoparietal junction (TPJ) was associated with higher Liking ratings (*BF*10 > 100), while synchrony between the left and right TPJ was associated with increased Bonding (*BF*10 > 100). Arrows indicate the corresponding ROI connections across hemispheres. The brain template was adapted from Canva.

For Liking, we tested mediation models for the ten ROI. Across all models, the indirect effects [average causal mediation effect (ACME)] were close to zero, and their 95% ETI included zero, indicating no evidence that neural synchrony mediated the relationship between Laughter and Liking. The direct effects (ADE), total effects, and proportion mediated estimates also had ETIs centered around zero, suggesting an overall absence of meaningful effects across models. A full summary of all path estimates, including ACME, ADE, total effect, and proportion mediated, is provided in Supplementary Tables 9, 10. Although no mediation effects were supported, in the mediation with Laughter Manipulation as treatment, the posterior estimate for the path from neural synchrony (during free interaction phase) to Liking was notably positive in the model involving IFGl_TPJr [estimate = 11926, 95% ETI (2.33, 20.23)]. In contrast, we found a negative effect from synchrony (during manipulation phase) between IFGr_TPJr [estimate = −13.99, 95% ETI (−27.25, −0.72)] and Laughter Behavior during the Manipulation phase as the treatment. To follow up on these findings, we conducted two exploratory model comparisons between a model that included IFGl_TPJr and Laughter versus a model that included Laughter only and a model with IFGr_TPJr and the Laughter Behavior during the Manipulation phase versus a model with the Laughter Behavior alone. The resulting Bayes factors were both *BF10* > 100, indicating strong evidence in favor of including IFGr_TPJl (during manipulation phase) with a negative association (Figure 5) and IFGl_TPJr (during free interaction) as predictors of Liking (Figure 6). This suggests that synchrony in these ROI-pairs may predict participants’ liking ratings independently of its potential mediating role. In addition to the findings reported above, we observed a consistent pattern across all mediation models that included Laughter Behavior during the Free Interaction phase as a predictor, synchrony in both experimental phases, and Liking as the outcome. Specifically, we found strong evidence for a direct effect of LBFI on Liking, with posterior estimates ranging between 10,105.61 [95% ETI (2231.29, 17850.61)] and 17,043.38 [95% ETI (7,017.90, 26,889.38)]. Furthermore, the total effect of the mediation models also showed strong evidence for an association between LBFI and Liking, with estimates ranging between 10,376.24 [95% ETI (2671.78, 18025.85)] and 16,660.26 [95% ETI (6,640.19, 26,737.53)]. These findings highlight a relationship between LBFI and Liking, regardless of the mediating role of neural synchrony.

For Prosociality, mediation models were tested for the three Laughter variables, all ROI pairs and for the two prosocial measurements (Giving Money and Helping Move). Across most of the models, the estimated indirect effects, direct effects, total effects, and proportion mediated were small, and their 95% ETIs nearly all included zero. This consistent pattern of posterior distributions centered around zero indicates that the data provide no evidence for a mediating role of neural synchrony in the relationship between Laughter and Prosociality. The observed effects are more likely under the null hypothesis than under models including a mediation pathway. However, in two mediation analyses, we found an direct effect of synchrony (during manipulation phase) for the ROI-pair IFGr_IFGl on Helping (for the mediation with Laugther Manipulation as treatment the effect from synchrony on Helping was 1.84 [95% ETI (0.12, 3.65)] and for the mediation with LBMP as treatment the effect from synchrony on Helping was 1.91 [95% ETI (0.13, 3.63)]. Full results for all path estimates are provided in Supplementary Tables 9, 10. To further investigate these findings, we conducted two exploratory model comparisons to assess the contribution of IFGr_IFGl as a predictor of Helping. In the first comparison, we compared a model including both IFGr_IFGl and Laughter Manipulation to a model with Laughter Manipulation alone, resulting in a Bayes factor of *BF10* = 34.13. In the second comparison, we compared a model including IFGr_IFGl and Laughter Behavior during the manipulation phase to a model with Laughter Behavior alone, yielding a Bayes factor of *BF10* = 41.31. Both comparisons provide strong evidence for the inclusion of IFGr_IFGl (during the manipulation phase) as a predictor of Helping (Figure 5).

To examine whether neural synchrony mediated the effect of Laughter on Bonding, we ran separate mediation models for the ROI pairs. None of the models yielded substantial evidence for an indirect effect: across most ROI pairs, the posterior estimates for ACME, ADE, total effect, and proportion mediated were small and their 95% ETIs included zero. This pattern suggests that, in the context of our data, synchrony did not mediate the link between Laughter and Bonding. Full model estimates are reported in Supplementary Tables 9, 10. However, for the ROI pair TPJr_TPJl, the path from neural synchrony (during free interaction phase) to IOS itself was notably positive [estimate = 10.71, 95% ETI (0.56, 20.75)]. To follow up on this finding, we conducted an exploratory Bayes analysis comparing a model including both TPJr_TPJl and Laughter manipulation against a model with Laughter manipulation only. The resulting Bayes factor was *BF*10 = > 100, providing extreme evidence for the inclusion of TPJr_TPJl. These results point to a potential direct association between neural synchrony in this ROI-pair and Bonding, independent of any mediating function (Figure 6). Furthermore, there was consistent evidence for a direct effect of LBFI on Bonding across thirteen models, including synchrony during manipulation phase (IFGl_IFGl, IFGl_TPJl, IFGl_TPJr, IFGr_IFGl, IFGr_IFGr, IFGr_TPJl, IFGr_TPJr, TPJr_TPJl) and synchrony during free interaction (IFGl_IFGl, IFGl_TPJl, IFGl_TPJr, IFGr_IFGl, TPJr_TPJr). The posterior estimates for the direct path ranged from 6665.35 [95% ETI (33.82, 13494.67)] to 9419.49 [95% ETI (524.72, 18085.24)]. Similarly, the total effect of LBFI on Bonding was robust, with estimates ranging from 6746.77 [95% ETI (71.24, 13578.20)] to 9285.13 [95% ETI (287.52, 18,128.71)]. These findings suggest that Laughter Behavior during the Free Interaction phase is associated with Bonding, independent of any mediating role of neural synchrony.

## 4 Discussion

The purpose of this study was to gain a better understanding of the relationship between Laughter and neural synchrony, and their impact on shared Liking, Prosocial Behavior, and Bonding. Contrary to our first hypothesis, we found no evidence that Laughter increased subsequent neural synchrony, neither alone nor in a social context. For our second hypothesis, we also did not find evidence from the parameter estimation analyses that Laughter significantly increased Liking, Prosociality, or Bonding. However, Liking after the manipulation was strongly predicted by pre-test levels. Importantly, model comparisons revealed strong evidence for the role of Laughter Behavior during both the manipulation phase and the free interaction phase, which was strongly associated with Prosociality, operationalized through increased Money Sharing, Bonding, and Liking. These findings suggest that Laughter Behavior, rather than the experimental manipulation alone, may play a key role in fostering positive social outcomes. Finally, regarding our third hypothesis, we did not find support for the proposed mediating role of synchrony in explaining the link between Laughter and positive social outcomes. However, we did observe direct paths from Laughter Behavior during the free interaction phase to both Liking and Bonding, further highlighting the importance of laughter in shaping social connections. Exploratory analyses also revealed four notable associations: increased synchrony between left IFG and right TPJ was linked to higher Liking, whereas synchrony between right IFG and right TPJ showed a negative association with Liking, increased synchrony between right and left IFG was linked to Prosociality (Helping), and synchrony between right TPJ and left TPJ predicted stronger Bonding. In the following sections, we will discuss these findings, considering existing theories and models of interpersonal synchrony.

### 4.1 Hypothesis 1: no evidence for enhanced neural synchrony through Laughter

The present study aimed to investigate the relationship between laughter and neural synchrony during both a structured laughter manipulation phase and a free interaction phase. The results did not provide evidence to suggest that neural synchrony during the manipulation phase was associated with laughter behavior or the experimental laughter manipulation. Participants who engaged in a laughter-inducing game and watched funny animal videos with recorded laughter did not show higher levels of neural synchrony compared to those in conditions with no induced laughter. This is notable, as it challenges the assumption that laughter, as a socially important and rhythmic signal, inherently facilitates neural alignment between individuals. Although laughter is described as a “social glue” (McGettigan et al., 2015), our results suggest that its effects on neural synchrony, particularly during structured activities, may be more complex than previously assumed. One possible explanation of this lack of evidence might relate to the relationship between the people laughing. Since participants in our study were strangers, the laughter they shared might have differed in quality or relevance compared to laughter shared between people who are emotionally closer, such as friends or romantic partners. Previous research has shown that people can tell whether others are friends or strangers just by hearing them laugh together (Bryant et al., 2016). It could also be that the kind of laughter elicited in interactions between strangers differs in its social or emotional quality. For example, polite or socially expected laughter might not have the same impact as spontaneous laughter driven by genuine amusement. Supporting this idea, McGettigan et al. (2015) found that the brain responds differently to authentic and posed laughter, suggesting that listeners automatically evaluate the emotional genuineness of laughter. This raises the possibility that, in our study, some instances of laughter may have been perceived or produced in ways that were less emotionally engaging. Given that participants did not know each other before the experiment, much of the laughter may have reflected politeness or social convention rather than shared emotional resonance. Such subtle differences in the quality of laughter within stranger dyads could potentially contribute to the absence of observable effects on neural synchrony. Future research could explore this further by more systematically characterizing laughter within the interaction itself. For example, recent work has proposed detailed taxonomies to distinguish different types of laughter in terms of their acoustic, social, and pragmatic features (Mazzocconi et al., 2020). Applying such fine-grained coding schemes may help identify which kinds of laughter are most likely to foster alignment between individuals. Taken together, the findings raise important questions about the conditions under which laughter might contribute to interpersonal synchrony and call for further studies to explore its role in different relational and contextual settings.

### 4.2 Hypothesis 2: tentative links between Laughter Behavior and social outcomes

We next consider the results regarding the second hypothesis, which proposed that laughter would enhance positive social outcomes. Although the parameter estimation approach again offered no evidence, Bayes factor comparisons did reveal informative contrasts. We found strong evidence for links between laughter behavior and positive social outcomes, including increased liking, prosocial behavior (money sharing), and bonding. These findings suggest that while the structured laughter manipulation itself may not have been sufficient to enhance social connectedness, the amount of laughter behavior exhibited by participants played a key role in fostering affiliative outcomes.

As already discussed in the context of H1, one plausible explanation for the null results of the parameter estimations is that laughter between strangers may differ in quality or meaning compared to laughter shared among familiar individuals. If the laughter produced in our study was more polite than genuinely shared, it may not have carried the affiliative or bonding potential often attributed to spontaneous, emotionally grounded laughter (Dunbar, 2012). This interpretation aligns with previous work suggesting that the social impact of laughter depends on both its acoustic authenticity and the relational context in which it occurs (Bryant et al., 2016; McGettigan et al., 2015). Additional evidence indicates that, especially in interactions between strangers, laughter may serve less as a reflection of genuine shared emotion and more as a navigating tool in unfamiliar social dynamics (Wood et al., 2022).

Another finding of our study was that participants’ initial liking ratings (Liking 1) predicted how much they liked each other after the interaction (Liking 2), with extreme evidence in favor of including this covariate in the model. This result highlights the powerful role of first impressions in shaping subsequent social evaluations. Previous research has shown that people form first impressions extremely quickly, often within just a fraction of a second. Willis and Todorov (2006) found that participants formed stable judgments about traits such as likeability after only 100 milliseconds of exposure to a face. These rapid judgments were highly correlated with ratings made under no time constraints, and longer viewing times did not significantly change the evaluations. Based on these findings, the authors concluded that first impressions are formed almost instantly and tend to remain consistent over time. This aligns with our results, in which initial liking strongly predicted later liking, suggesting that early affective impressions may serve as an anchor for subsequent evaluations. Taken together, our findings indicate that in interactions between strangers, initial impressions may be important for shaping liking as a social outcome than shared laughter experience alone. Future research could examine how initial liking and other first-impression evaluations influence laughter behavior, complementing our focus on the effects of laughter on social outcomes.

However, our results from the Bayesian model comparisons revealed strong evidence that the laughter behavior, both during the manipulation phase and the free interaction phase, was consistently associated with all three outcome variables: prosociality (money sharing), bonding, and liking. These findings support Hypothesis 2, indicating that laughter is indeed linked to bonding, liking, and at least one aspect of prosociality. However, it is important to note that no evidence was found for an association between laughter and the helping measure of prosociality. Future research could explore this further by examining different dimensions of prosocial behavior and their relationship with laughter. Previous work, such as that by Dunbar et al. (2021), has also reported complex and nuanced findings in this area, suggesting that the connection between laughter and prosociality may depend on specific contexts or types of prosocial behavior. Overall, while our findings align with prior research indicating that laughter can foster social connectedness, they also highlight the need for further investigation. Future studies with larger sample sizes may help to clarify these relationships and potentially detect effects in parameter estimation analyses that were not observed in the current study.

### 4.3 Hypothesis 3: absence of mediation but exploratory ROI-specific neural synchrony and Laughter Behavior associations with Liking and Bonding

To test our third hypothesis, we explored whether neural synchrony mediated the relationship between laughter and the three social outcome variables: liking, prosociality, and bonding. Although the overall pattern showed no evidence for mediation effects, indirect effects were consistently small and their ETIs included zero; some findings stood out.

Specifically, the findings revealed consistent evidence for direct associations between laughter behavior during the free interaction phase and the social outcome variables of liking and bonding. In all mediation models examining liking, we found a direct link between laughter behavior during the free interaction phase and liking, which was not mediated by neural synchrony during this phase. Similarly, for bonding, mediation models revealed a direct association between laughter behavior during the free interaction phase and bonding, again without evidence for mediation by synchrony. Interestingly, this pattern was not observed for laughter behavior during the manipulation phase, suggesting that the context and timing of laughter may play a critical role in its effects on social outcomes. Nevertheless, these findings, in combination with the results from Hypothesis 2, further underscore the importance of laughter behavior in fostering social connectedness. The consistent associations between laughter behavior during free interaction and both liking and bonding provide compelling evidence that especially unprompted and spontaneous laughter can have an immediate and direct impact on these social outcomes. This highlights the need for further research to better understand the mechanisms through which laughter influences social bonding and liking over time, particularly in naturalistic social interactions.

Moreover, the outcome measures of liking and bonding were also linked to interpersonal neural synchrony. First, in models predicting liking, synchrony (during free interaction) in the IFGl_TPJr ROI pair showed a clear positive association with the outcome. Exploratory follow up analyses showed that interpersonal synchrony between the left IFG and the right TPJ predicted how much participants liked each other. Interestingly, a contrasting finding emerged regarding synchrony during the manipulation phase: synchrony between the right IFG and the right TPJ showed a negative effect on liking, which was further confirmed in a follow-up analysis, providing strong evidence for this effect. These two findings; one showing a positive effect of synchrony during free interaction in one ROI pair and the other showing a negative effect of synchrony during manipulation in a different ROI pair, may point to distinct functional roles of these ROI pairs in predicting liking. Moreover, this contrast highlights the complexity of the relationship between interpersonal synchrony and liking, suggesting that the timing and context of synchrony, as well as the specific brain regions involved, play critical roles in shaping social outcomes (further discussion and interpretation of these results are provided in section 4.4.)

The second notable finding emerged in the model predicting bonding, where a follow-up analysis showed that interpersonal synchrony (during free interaction) between the right and the left TPJ was positively related to participants’ sense of closeness to each other, a core component of bonding. In addition to liking and bonding, we also found an interesting result related to prosociality. Specifically, synchrony between the right IFG and the left IFG was positively associated with helping behavior. What is particularly interesting across these findings is that different ROI pairs are consistently associated with specific outcome variables rather than a single ROI pair being linked to all outcomes. This suggests that synchrony in different brain regions may play specialized roles in shaping distinct aspects of social interaction. Together, these results highlight how synchrony can influence social outcomes in nuanced and context-dependent ways.

To better understand the nature of these exploratory links between neural synchrony and social outcomes, it is essential to examine the specific brain regions involved. In the following section, we therefore provide a functional overview of the IFG and the TPJ, which were implicated in the synchrony-outcome associations. This context may help clarify the potential roles these brain areas play in shaping interpersonal alignment and social connection.

### 4.4 Brain areas implicated in synchrony and social outcomes

The right TPJ appeared in ROI pairs associated with the results linking neural synchrony during the free interaction phase to our exploratory findings on liking and bonding. Prior studies have consistently implicated the right TPJ in self-other distinction, attentional shifting, and empathy-related processes (Ahmad et al., 2021), as well as bilateral TPJ in mentalizing—the ability to infer and interpret the mental states of others, including Theory of Mind (Arioli et al., 2021; Lotter et al., 2023; Monticelli et al., 2021; Saxe and Wexler, 2005).

The functional role of the right TPJ is specifically highlighted by Ahmad et al. (2021), who summarize findings from transcranial magnetic and direct current stimulation studies. Their review shows that stimulation of this region influences self-other differentiation, modulates attentional reorienting, and affects how individuals respond empathetically to others depicted in emotional video stimuli. These findings may help explain why right TPJ synchrony in our study was linked to participants’ social responses, as these functions are crucial for dynamically adapting to another person in real-time interaction, including keeping track of shifting perspectives, adjusting one’s own behavior in response to social cues, and regulating self-other boundaries in shared communicative space. Additional insights into the predictive functions of the TPJ come from Doricchi et al. (2022, 2023), who conceptualize the region as a match–mismatch detector in social cognition. According to their framework, the right TPJ responds to violations of social expectations, while the left TPJ tracks alignment between one’s own and others’ perspectives. In their updated account, the TPJ not only detects discrepancies but also builds predictive templates of expected social events, adjusting them dynamically through experience (Doricchi et al., 2023). These anticipatory functions may help explain the TPJ’s role in our study, where real-time adjustments to partner behavior were likely crucial. This framework could also shed light on why the right TPJ was involved in the negative association with liking during the manipulation phase, as mismatches or violations of social expectations may have contributed to reduced liking in this context.

Furthermore, Monticelli et al. (2021) report that the TPJ is consistently engaged during mentalizing tasks, with a tendency for stronger activation in the right hemisphere. Similarly, Arioli et al. (2021) conducted a meta-analysis and found bilateral TPJ activation across both cognitive and affective mentalizing tasks. Notably, cognitive mentalizing recruited the TPJ in both hemispheres, while affective mentalizing showed consistent engagement of the left TPJ (Monticelli et al., 2021). These findings support the TPJ’s central role in representing others’ mental states and point to hemispheric differences in how different forms of social inference are processed. In our study, the involvement of the right TPJ may reflect participants’ efforts to continuously interpret and adjust to their partner’s communicative cues. The left TPJ, on the other hand, may be more involved in affective processes, which, in combination with laughter behavior, could support the development of bonding.

In addition, the left IFG was involved in the ROI pair that predicted liking. Arioli et al. (2021) report that affective mentalizing was associated with stronger activation in the left IFG, particularly during tasks involving emotional facial expressions. The same study found that both affective mentalizing and empathy activated overlapping areas in the left IFG. This could support the idea that, in our study, the involvement of the left IFG reflects sensitivity to emotional cues during the interaction, which in turn may support likeability between the participants. The right IFG, which was part of the ROI pair negatively associated with liking during the manipulation phase, is also involved in affective mentalizing such as emotional contagion and empathy (Arioli et al., 2021). These functions suggest that the right IFG plays a role in detecting and responding to emotional dynamics during social interactions. In our study, the negative association between synchrony in the right IFG and liking may reflect disruptions in these processes during the manipulation phase, potentially due to mismatches in emotional expectations or incongruities in the interaction.

#### 4.4.1 Considering the findings in the context of current interpersonal neural synchrony research

Our exploratory findings align well with the social alignment model proposed by Shamay-Tsoory et al. (2019), which conceptualizes synchrony as part of a neural feedback loop comprising misalignment detection, observation–execution, and reward systems. Within this loop, the IFG encodes motor representations of actions, enabling the generation of congruent responses during social interaction. In parallel, regions involved in self–other distinction, particularly the TPJ, are thought to support alignment by flexibly managing social perspective-taking (Shamay-Tsoory et al., 2019).

The synchrony between left IFG and right TPJ, which predicted liking, may thus reflect the joint recruitment of these core alignment mechanisms: left IFG contributing to affective attunement, and right TPJ supporting dynamic adjustment to a partner’s behavior and mental state. According to Shamay-Tsoory et al. (2019), alignment processes not only facilitate moment-to-moment coordination but also increase subjective likeability. Our findings may therefore reflect a socially rewarding alignment process that made interaction partners more positively evaluated. Additionally, our findings are also consistent with recent meta-analytic evidence identifying the left IFG and right TPJ as central contributors to interpersonal neural synchrony. In their large-scale integration of fMRI and fNIRS hyperscanning studies, Lotter et al. (2023) concluded that the right TPJ supports the encoding and integration of social attention and mental state information, whereas the left IFG facilitates behavioral alignment through mirroring and motor representations. Together, these regions are thought to subserve the observation–execution subsystem of the social alignment system, enabling individuals to dynamically coordinate their actions and attention during social interaction; an interpretation that aligns closely with the framework proposed by Shamay-Tsoory et al. (2019). At the same time, the negative association between synchrony in the right IFG and right TPJ during the manipulation phase suggests that alignment processes may not always be socially rewarding and could depend on the specific dynamics of the interaction. Synchrony during the manipulation phase may engage alignment mechanisms in ways that do not lead to positive evaluations, highlighting a more complex relationship between alignment and reward systems. Future studies are needed to better understand how different interaction contexts shape the social outcomes of neural synchrony.

Our findings of neural synchrony, including left and right TPJ, are consistent with the proposed role of the TPJ in processing dynamic social information. As outlined by Hoehl et al. (2021), the TPJ integrates input from multiple modalities and supports the temporal coordination of socially relevant cues, such as those encountered during interactive exchanges. This function may be particularly relevant in our conversational task, where timing and the integration of verbal and nonverbal signals are essential. At the same time, our findings align with the framework proposed by Shamay-Tsoory et al. (2019), contending that connectedness between individuals emerges as a fundamental outcome of social alignment. This conceptualization fits well with our operationalization of bonding via perceived closeness, suggesting that the increased interpersonal connectedness measured behaviorally may be rooted in the social reward mechanisms triggered by successful neural synchrony.

#### 4.4.2 Synchrony task: free conversation

In our study, interpersonal brain synchrony was measured during a joint video watching task (manipulation phase) and a face-to-face free conversation task. To better understand the observed links between laughter behavior, social outcomes, and synchrony during the conversation, we now examine the nature of the free-conversation task and its implications for interpersonal synchrony.

As participants engaged in verbal interaction, it is important to note that both the IFG and the TPJ are known to play key roles in language processing (Hertrich et al., 2020). Furthermore, the free conversation task in our study relies on the effective coordination of verbal and nonverbal behavior between partners. This typically involves one person speaking while the other listens, followed by a role change. To manage this flow, partners need to take turns, organizing their contributions in a way that enables mutual understanding. This turn-taking mechanism plays a central role in structuring conversation and has been linked to neural synchrony (De Felice et al., 2024). For example, Nguyen et al. (2020) showed that neural synchrony between mothers and their preschool-aged children increased throughout a naturalistic verbal conversation, particularly in bilateral TPJ regions, and that this increase was closely associated with the amount of conversational turn-taking within the dyad. Furthermore, Jiang et al. (2015) found increased synchronization in the left TPJ of leader–follower dyads engaged in face-to-face discussions. Importantly, this synchrony was not simply driven by the number of conversational turns but was linked to the communicative quality of leader-initiated contributions. These findings suggest that neural alignment during conversation reflects subtle social coordination processes, such as mutual adaptation, role differentiation, and shared attention. In our study, participants also engaged in verbal interaction, which likely involved similar processes of social alignment. The neural synchrony we observed may therefore capture how partners continuously adjust to each other’s communicative behavior, possibly reflecting emergent asymmetries in how information is processed and produced across the dyad. This perspective may also help explain why synchrony emerged between functionally distinct brain regions, such as the left IFG and right TPJ. Conversational exchange requires different cognitive operations on each side, including speech production, perspective-taking, and the interpretation of social cues. Taken together, our findings suggest that neural synchrony during conversation is shaped by differentiated but complementary neural processes that allow interaction partners to remain attuned to each other across changing roles and communicative demands. However, due to the limited spatial resolution of fNIRS, any conclusions about the involvement of specific brain areas must be interpreted with caution. Our selection of regions of interest was informed by prior literature and anatomical approximations, but the resulting findings should be viewed in light of the methodological constraints inherent to this technique

### 4.5 Limitations and future directions

This study adds to the emerging field of laughter and interpersonal neural synchrony research by addressing several open questions. It introduces a novel social-affective stimulus, laughter, as a potential trigger for interpersonal synchrony, examines the affiliative outcomes of neural synchrony, and employs a naturalistic interaction context. These elements make the current work a valuable contribution to the conceptual and methodological development of laughter and synchrony research. Despite its novel approach, some limitations should be acknowledged. One limitation of the present study concerns the challenge of reliably eliciting or inhibiting laughter in an experimental laboratory setting. Although the manipulation aimed to induce laughter, the actual amount of laughter displayed by participants varied considerably. Therefore, we included the proportion of time spent laughing in our analyses and showed that the relative laughter duration was successfully manipulated in our experimental conditions and relates to some of our variables of interest. This approach is in line with previous studies that have used auditory laughter stimuli to investigate the effects of laughter (Nummenmaa et al., 2023). A second methodological consideration relates to the analysis of neural synchrony itself. While WTC is a widely used method in synchrony research, there remains considerable heterogeneity in how it is applied across studies. Differences in analytical choices, such as frequency ranges, time windows, and the handling of lagged versus concurrent synchrony, make it challenging to directly compare results or establish standardized benchmarks. As highlighted by Ohayon and Gordon (2025) The field lacks methodological consensus, which may contribute to variability in reported effects and complicates the interpretation of findings across studies. Another consideration concerns the sample size, which may have been relatively small for a full 2 × 2 design, and given the complexity of our statistical models, as data collection had to be terminated due to diminishing resources rather than reaching the stopping cut-off for conclusive Bayes factors. This limitation should be kept in mind when interpreting the findings, as the strength of the evidence for some effects may remain inconclusive. While this may have limited the power to detect effects in the parameter estimation analyses, it reflects the methodological demands of hyperscanning research. Particularly when studying spontaneous social behaviors such as laughter, which require simultaneous measurement of two interacting participants. Furthermore, one has to consider that the surrogate data analysis indicates, as a group, the real pairs of participants did not show higher synchrony compared to random pairs. While this indicates that there is no substantial neural synchronization across the whole sample, individual participant pairs still showed different levels of synchrony; thus, we decided to include the neural data in our analysis. This variability may be particularly pronounced during the free conversation phase, where factors such as the content and dynamics of the conversation and laughter behavior, likely contributed to differences in neural synchrony across dyads. These factors represent inherent challenges in hyperscanning research, especially when studying spontaneous and naturalistic social interactions. We have aimed to frame our findings in relation to the specific conditions of the study rather than as general evidence for synchrony versus no synchrony.

Despite the mentioned constraints, the study offers important initial insights into neural synchrony during naturalistic laughter-based interactions and demonstrates the feasibility of investigating such phenomena in real-time dyadic settings. Importantly, it represents one of the few attempts to systematically examine laughter within a controlled yet ecologically valid experimental paradigm, thereby providing a valuable foundation for future research on laughter, synchrony, and social outcomes. This line of research holds practical relevance: laughter has been recognized as a low-cost, accessible tool to promote social cohesion, reduce stress, and improve health outcomes (Tunaligil, 2025). Understanding the neural mechanisms underlying these effects can inform the development of targeted interventions aimed at strengthening emotional resilience and wellbeing (Delgado et al., 2023). These processes serve as important buffers against stress and isolation and could be utilized as protective factors in public health. Findings from this research could therefore inform therapeutic, educational, or policy-level interventions that aim to promote social connection and emotional wellbeing. To that end, this study contributes to a broader understanding of how everyday social experiences, like laughter, can shape human connection, resilience, and long-term wellbeing. While our study provides a valuable starting point, several questions remain open for future investigation. For example, our study exclusively included stranger dyads, which may have limited the degree of neural synchrony observed due to the absence of established social bonds. Increasing evidence points to a bidirectional relationship between interpersonal synchrony and bonding, where each process mutually reinforces the other (Hoehl et al., 2021; Shamay-Tsoory et al., 2019). Building on this, De Felice et al. (2024) report that interpersonal brain synchrony tends to be stronger in familiar dyads, suggesting that social closeness enhances neural synchrony. Future research should investigate laughter and neural synchrony in dyads with closer relationships to better capture these bidirectional dynamics between social bonding and synchrony.

## 5 Conclusion

All in all, the present study provides important insights into the interplay between laughter behavior, neural synchrony, and positive social outcomes. First, we found that laughter during both the manipulation phase and the free interaction phase was linked to positive social outcomes, including increased liking, bonding, and prosociality (as measured by money-sharing behavior), though this latter finding was less robust and requires further investigation. Second, neural synchrony during the manipulation phase showed a positive association between synchrony in bilateral IFG and prosociality (measured through helping behavior). Additionally, we observed a negative association between synchrony in right IFG and right TPJ and liking during this phase, highlighting the complexity of these neural mechanisms. Third, neural synchrony during the conversation task, particularly in bilateral TPJ and left IFG, was linked to increased liking and bonding, two essential components of meaningful human connection. These heterologous synchrony patterns did not simply reflect symmetrical or mirrored activations across hemispheres, pointing to complementary roles across participants, consistent with a model of co-regulated, dialogic exchange rather than parallel processing in the same brain areas (Jiang et al., 2021). Our results revealed meaningful, functionally differentiated pairings between distinct regions. Such cross-brain, cross-region coupling could facilitate reciprocal coordination, where one partner tracks and interprets social cues while the other formulates timely, socially appropriate responses. While the results provide valuable insights, it is important to keep in mind that data collection was concluded before conclusive Bayes factors could be reached for all comparisons. This context should be considered when interpreting the findings, particularly for effects where the evidence remains inconclusive.

In summary, our findings underscore the role of laughter in shaping social outcomes. Laughter behavior was associated with key indicators of social connection, such as liking and bonding, with some evidence for prosociality. Additionally, neural synchrony was also linked to social outcomes, with distinct patterns of association depending on the brain regions involved, including both positive and negative relationships. However, the absence of a mediating role for neural synchrony highlights the need for future research to explore alternative mechanisms and to further investigate how laughter and synchrony interact to foster meaningful human connections across different contexts and timescales. Future work could examine more local, time-locked effects of laughter on neural synchrony, for example, by aligning neural signals to laughter onsets and offsets to assess transient changes. Moreover, to our knowledge, this is the first study to investigate the connection between laughter and neural synchrony, and our results represent useful information that paves the way for further ecologically valid research on the neural underpinnings of laughter and social connection.

## Data Availability

The datasets presented in this study can be found in online repositories. The names of the repository/repositories and accession number(s) can be found at: OSF (https://osf.io/x8w4n/?view_only=afff50ff8d264f79be7b77955e4fc525).
