# Supplementary material for: In sync through laughter? An fNIRS hyperscanning study on neural synchrony and social connection
Source: Front Neurosci. 2026 Jan 20;19:1697100. doi: 10.3389/fnins.2025.1697100 (PMC12865808; doi:10.3389/fnins.2025.1697100)
Supplement: Supplementary file 1 [file Data_Sheet_1.docx]

Supplementary Material

# Supplementary Figures and Tables

## Figures


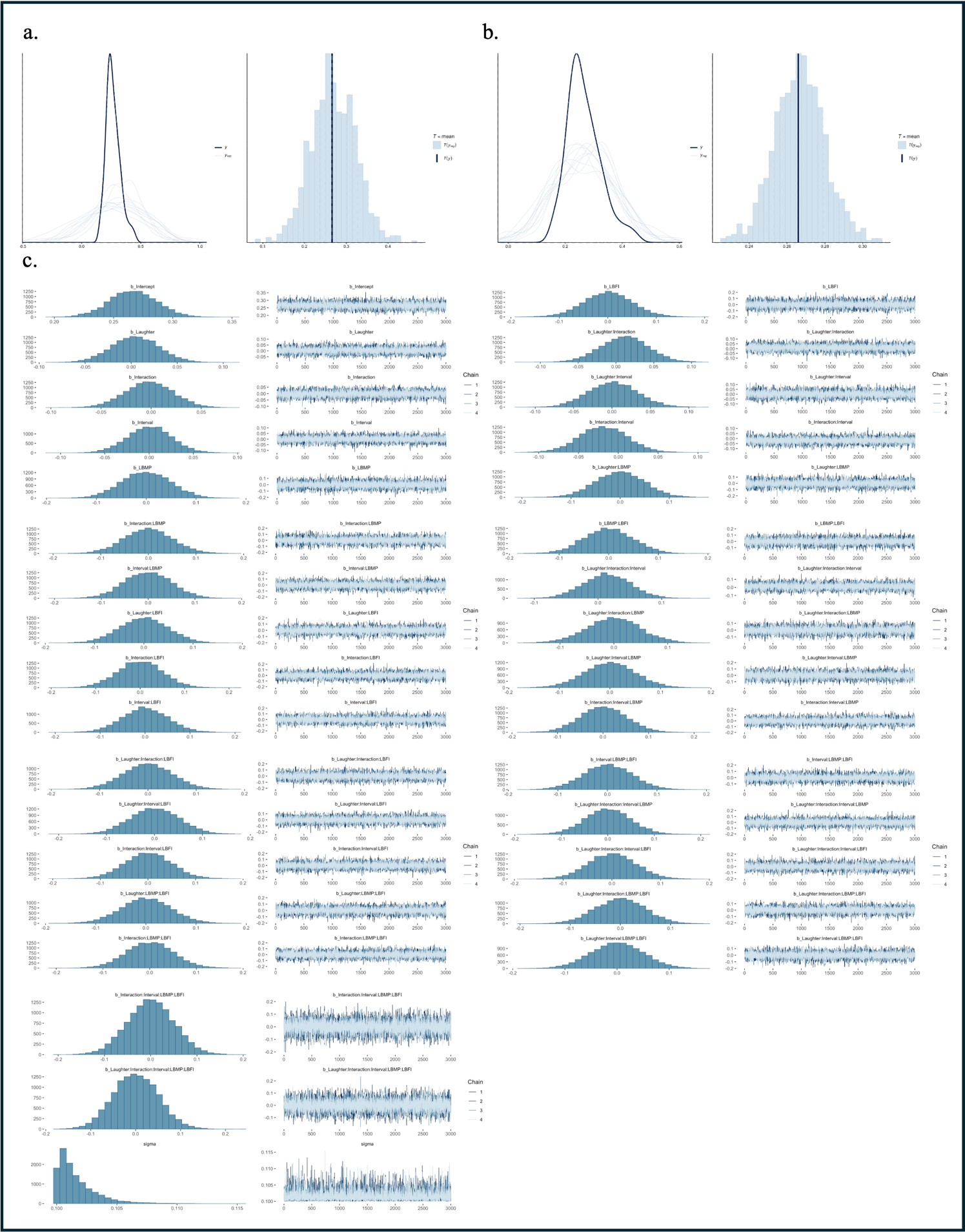
Figure 1. Bayesian workflow example for the ROI pair IFGr_IFGr, during the free interaction phase, with all parameter plots. Panel (a) shows prior predictive checks before model fitting. The density overlay compares prior predictive draws (light lines) with the observed data distribution (bold line), followed by a comparison of the mean statistic. Panel (b) shows the posterior predictive checks comparing the model’s predictions to the observed data, both in terms of the full distribution (density overlay) and the mean as a summary statistic. Panel (c) presents diagnostics and model evaluation after fitting the final model. The left column displays the posterior distributions of all model parameters, while the right column shows their respective trace plots across four chains, indicating convergence. Laughter Behavior during the Manipulation phase (LBMP) and Laughter Behavior during the Free Interaction phase (LBFI).

## Supplementary Tables

| **ROI pair** | **Real Estimate [95% CI]** | **Interval Estimate [95% CI]** | **Real*Interval Estimate [95% CI]** |
| --- | --- | --- | --- |
| **IFGl_IFGl** | 0.00 [-0.02, 0.03] | -0.00 [-0.02, 0.02] | -0.00 [-0.04, 0.02] |
| **IFGl_TPJl** | 0.00 [-0.01, 0.02] | 0.00 [-0.01, 0.02] | -0.00 [-0.03, 0.02] |
| **IFGl_TPJr** | 0.00 [-0.01, 0.02] | 0.00 [-0.01, 0.02] | -0.00 [-0.03, 0.02] |
| **IFGr_IFGl** | 0.00 [-0.01, 0.02] | -0.00 [-0.01, 0.01] | -0.00 [-0.02, 0.02] |
| **IFGr_IFGr** | 0.00 [-0.02, 0.03] | -0.00 [-0.02, 0.02] | 0.00 [-0.03, 0.04] |
| **IFGr_TPJl** | 0.00 [-0.01, 0.02] | 0.00 [-0.01, 0.02] | -0.00 [-0.03, 0.02] |
| **IFGr_TPJr** | 0.00 [-0.01, 0.02] | 0.00 [-0.01, 0.02] | -0.00 [-0.02, 0.02] |
| **TPJl_TPJl** | 0.00 [-0.02, 0.03] | 0.00 [-0.02, 0.03] | -0.00 [-0.04, 0.03] |
| **TPJr_TPJl** | 0.00 [-0.01, 0.02] | 0.00 [-0.01, 0.02] | -0.00 [-0.04, 0.02] |
| **TPJr_TPJr** | 0.01 [-0.01, 0.03] | 0.00 [-0.02, 0.02] | -0.02 [-0.06, 0.01] |

Table 1. This table reports the Bayesian parameter estimates for synchrony during the manipulation phase, comparing real data and surrogate data (random permutation). Predictors include Real (real data vs. surrogate data), Interval (first 5-minute video vs. second 5-minute video), and the interaction term Real * Interval. The analysis was conducted for 10 ROI pairs, consisting of the inferior frontal gyrus (IFG) and the temporoparietal junction (TPJ), labeled as "l" (left hemisphere) and "r" (right hemisphere).

| **ROI pair** | **Real Estimate [95% CI]** | **Interval Estimate [95% CI]** | **Real*Interval Estimate [95% CI]** |
| --- | --- | --- | --- |
| **IFGl_IFGl** | -0.00 [-0.03, 0.01] | 0.00 [-0.02, 0.02] | 0.01 [-0.02, 0.04] |
| **IFGl_TPJl** | 0.00 [-0.01, 0.02] | -0.00 [-0.02, 0.01] | -0.00 [-0.03, 0.02] |
| **IFGl_TPJr** | -0.00 [-0.02, 0.01] | 0.00 [-0.01, 0.02] | 0.01 [-0.01, 0.04] |
| **IFGr_IFGl** | 0.00 [-0.01, 0.02] | 0.00 [-0.01, 0.02] | -0.00 [-0.03, 0.02] |
| **IFGr_IFGr** | 0.00 [-0.02, 0.03] | 0.00 [-0.02, 0.02] | -0.00 [-0.04, 0.03] |
| **IFGr_TPJl** | 0.00 [-0.01, 0.02] | -0.00 [-0.02, 0.02] | -0.00 [-0.03, 0.03] |
| **IFGr_TPJr** | 0.00 [-0.02, 0.02] | 0.00 [-0.01, 0.02] | 0.00 [-0.02, 0.03] |
| **TPJl_TPJl** | 0.00 [-0.02, 0.03] | -0.00 [-0.03, 0.02] | -0.00 [-0.04, 0.03] |
| **TPJr_TPJl** | 0.00 [-0.02, 0.02] | -0.00 [-0.02, 0.02] | 0.00 [-0.02, 0.03] |
| **TPJr_TPJr** | 0.00 [-0.02, 0.04] | 0.00 [-0.02, 0.02] | 0.00 [-0.03, 0.04] |

Table 2. This table reports the Bayesian parameter estimates for synchrony during the free interaction phase, comparing real data and surrogate data (random permutation). Predictors include Real (real data vs. surrogate data), Interval (first 5-minute segment vs. second 5-minute segment), and the interaction term Real * Interval. The analysis was conducted for 10 ROI pairs, consisting of the inferior frontal gyrus (IFG) and the temporoparietal junction (TPJ), labeled as "l" (left hemisphere) and "r" (right hemisphere).

| **Parameter Estimate [95% CI]** | **IFGl_ IFGl** | **IFGl_ TPJl** | **IFGl_ TPJr** | **IFGr_ IFGl** | **IFGr_ IFGr** | **IFGr_ TPJl** | **IFGr_ TPJr** | **TPJl_ TPJl** | **TPJr_ TPJl** | **TPJr_ TPJr** |
| --- | --- | --- | --- | --- | --- | --- | --- | --- | --- | --- |
| **Sample size** | N (Dyad) = 95  N( Interval 1) = 95  N (Interval 2) = 94 | N (Dyad) = 94  N( Interval 1) = 160  N (Interval 2) = 155 | N (Dyad) = 94  N( Interval 1) = 160  N (Interval 2) = 156 | N (Dyad) = 96  N( Interval 1) = 181  N (Interval 2) = 177 | N (Dyad) = 87  N( Interval 1) = 86  N (Interval 2) = 83 | N (Dyad) = 88  N( Interval 1) = 152  N (Interval 2) = 145 | N (Dyad) = 89  N( Interval 1) = 152  N (Interval 2) = 146 | N (Dyad) = 68  N( Interval 1) = 66  N (Interval 2) = 63 | N (Dyad) = 78  N( Interval 1) = 132  N (Interval 2) = 126 | N (Dyad) = 68  N( Interval 1) = 66  N (Interval 2) = 63 |
| **Intercept** | 0.28 [0.25, 0.31] | 0.26 [0.24, 0.29] | 0.27 [0.24, 0.30] | 0.28 [0.25, 0.30] | 0.28 [0.25, 0.31] | 0.27 [0.25, 0.30] | 0.27 [0.24, 0.30] | 0.28 [0.24, 0.32] | 0.28 [0.25, 0.31] | 0.28 [0.24, 0.32] |
| **Laughter Manipulation** | -0.01 [-0.05, 0.02] | 0.00 [-0.02, 0.04] | 0.00 [-0.03, 0.03] | -0.02 [-0.05, 0.01] | -0.01 [-0.06, 0.02] | 0.00 [-0.03, 0.03] | 0.00 [-0.02, 0.04] | -0.00 [-0.05, 0.04] | 0.00 [-0.03, 0.03] | 0.01 [-0.03, 0.05] |
| **Social** | 0.00 [-0.04, 0.04] | 0.00 [-0.03, 0.03] | -0.00 [-0.03, 0.03] | 0.01 [-0.01, 0.04] | 0.00 [-0.03, 0.05] | -0.00 [-0.03, 0.03] | -0.00 [-0.04, 0.03] | -0.00 [-0.05, 0.04] | -0.01 [-0.04, 0.02] | 0.00 [-0.04, 0.05] |
| **LBMP** | -0.00 [-0.09, 0.09] | 0.00 [-0.09, 0.09] | -0.00 [-0.10, 0.09] | 0.00 [-0.09, 0.09] | 0.00 [-0.09, 0.10] | 0.00 [-0.09, 0.09] | -0.00 [-0.09, 0.09] | -0.00 [-0.09, 0.09] | -0.00 [-0.10, 0.10] | 0.00 [-0.09, 0.09] |
| **Interval** | -0.00 [-0.04, 0.04] | -0.01 [-0.04, 0.02] | -0.01 [-0.04, 0.02] | -0.00 [-0.03, 0.02] | -0.01 [-0.05, 0.03] | -0.00 [-0.04, 0.02] | -0.00 [-0.04, 0.03] | -0.01 [-0.06, 0.03] | -0.02 [-0.06, 0.00] | -0.01 [-0.06, 0.03] |
| **Laughter Manipulation:Social** | 0.01 [-0.04, 0.06] | -0.00 [-0.05, 0.04] | -0.00 [-0.05, 0.04] | 0.00 [-0.04, 0.04] | 0.00 [-0.05, 0.05] | -0.00 [-0.05, 0.04] | -0.00 [-0.05, 0.04] | -0.00 [-0.06, 0.05] | -0.01 [-0.06, 0.03] | -0.00 [-0.06, 0.05] |
| **Laughter Manipulation:Interval** | 0.00 [-0.04, 0.06] | 0.00 [-0.04, 0.05] | 0.00 [-0.04, 0.05] | 0.01 [-0.02, 0.06] | 0.02 [-0.03, 0.07] | 0.01 [-0.03, 0.05] | -0.00 [-0.05, 0.04] | 0.00 [-0.05, 0.06] | 0.02 [-0.02, 0.06] | -0.01 [-0.07, 0.04] |
| **Social:Interval** | -0.02 [-0.07, 0.03] | 0.00 [-0.03, 0.05] | 0.01 [-0.03, 0.05] | -0.01 [-0.05, 0.03] | -0.00 [-0.05, 0.05] | 0.00 [-0.04, 0.04] | 0.00 [-0.04, 0.05] | 0.00 [-0.05, 0.06] | 0.02 [-0.02, 0.07] | 0.01 [-0.04, 0.06] |
| **Laughter Manipulation:LBMP** | 0.00 [-0.09, 0.09] | -0.00 [-0.09, 0.09] | -0.00 [-0.09, 0.10] | -0.00 [-0.09, 0.09] | 0.00 [-0.09, 0.09] | -0.00 [-0.09, 0.09] | -0.00 [-0.09, 0.09] | 0.00 [-0.09, 0.09] | 0.00 [-0.09, 0.09] | 0.00 [-0.09, 0.09] |
| **Social:LBMP** | 0.00 [-0.09, 0.09] | -0.00 [-0.09, 0.09] | 0.00 [-0.09, 0.09] | 0.00 [-0.10, 0.10] | -0.00 [-0.09, 0.09] | 0.00 [-0.09, 0.09] | 0.00 [-0.09, 0.09] | -0.00 [-0.09, 0.09] | -0.00 [-0.09, 0.09] | -0.00 [-0.09, 0.09] |
| **Interval:LBMP** | 0.00 [-0.09, 0.10] | -0.00 [-0.09, 0.09] | 0.00 [-0.09, 0.09] | -0.00 [-0.09, 0.10] | 0.00 [-0.09, 0.09] | 0.00 [-0.09, 0.09] | 0.00 [-0.09, 0.09] | -0.00 [-0.09, 0.09] | -0.00 [-0.09, 0.09] | 0.00 [-0.09, 0.10] |
| **Laughter Manipulation:Social:Interval** | 0.00 [-0.06, 0.06] | 0.00 [-0.05, 0.06] | -0.00 [-0.06, 0.05] | 0.00 [-0.05, 0.06] | 0.00 [-0.06, 0.06] | 0.01 [-0.04, 0.07] | 0.01 [-0.04, 0.07] | 0.01 [-0.05, 0.09] | 0.00 [-0.05, 0.07] | -0.01 [-0.08, 0.05] |
| **Laughter Manipulation:Social:LBMP** | 0.00 [-0.09, 0.10] | -0.00 [-0.09, 0.09] | 0.00 [-0.09, 0.09] | 0.00 [-0.09, 0.09] | 0.00 [-0.09, 0.09] | 0.00 [-0.09, 0.09] | -0.00 [-0.09, 0.09] | -0.00 [-0.09, 0.09] | 0.00 [-0.09, 0.09] | -0.00 [-0.09, 0.09] |
| **Laughter Manipulation:Interval:LBMP** | 0.00 [-0.09, 0.09] | 0.00 [-0.10, 0.09] | -0.00 [-0.09, 0.09] | -0.00 [-0.10, 0.09] | 0.00 [-0.09, 0.09] | 0.00 [-0.09, 0.09] | 0.00 [-0.09, 0.09] | 0.00 [-0.09, 0.09] | 0.00 [-0.09, 0.09] | 0.00 [-0.09, 0.09] |
| **Social:Interval:LBMP** | -0.00 [-0.09, 0.09] | 0.00 [-0.09, 0.09] | 0.00 [-0.09, 0.09] | -0.00 [-0.09, 0.09] | 0.00 [-0.09, 0.09] | -0.00 [-0.09, 0.09] | 0.00 [-0.09, 0.09] | 0.00 [-0.09, 0.09] | 0.00 [-0.09, 0.09] | -0.00 [-0.10, 0.09] |
| **Laughter Manipulation:Social:Interval:LBMP** | 0.00 [-0.09, 0.09] | 0.00 [-0.09, 0.09] | -0.00 [-0.10, 0.09] | 0.00 [-0.09, 0.10] | 0.00 [-0.09, 0.09] | 0.00 [-0.09, 0.09] | 0.00 [-0.09, 0.09] | -0.00 [-0.09, 0.09] | -0.00 [-0.09, 0.09] | 0.00 [-0.09, 0.09] |

**Table 3.** Posterior Estimates for all Effects from the Bayesian parameter estimations by ROI Pair for synchrony measured during the manipulation phase. The analysis was conducted for 10 ROI pairs, consisting of the inferior frontal gyrus (IFG) and the temporoparietal junction (TPJ). The ROIs are labeled as "l" (left hemisphere) and "r" (right hemisphere). The model includes the following predictors: Laughter Manipulation(yes/no), Social Context (yes/no), Interval (first or second 5-minute video), and LBMP (laughter behavior during the manipulation phase). Sample sizes for Interval 1 and Interval 2 refer to the number of WTC values; each dyad contributes one measure per interval, resulting in two per dyad overall. For heterogeneous ROI pairs, sample sizes are higher than for homologous pairs because each pair includes bidirectional synchrony (e.g., TPJl-IFGr and IFGr-TPJl), both of which are included together for one ROI-pair analysis.

| **Parameter Estimate [95% CI]** | **IFGl_ IFGl** | **IFGl_ TPJl** | **IFGl_ TPJr** | **IFGr_ IFGl** | **IFGr_ IFGr** | **IFGr_ TPJl** | **IFGr_ TPJr** | **TPJl_ TPJl** | **TPJr_ TPJl** | **TPJr_ TPJr** |
| --- | --- | --- | --- | --- | --- | --- | --- | --- | --- | --- |
| **Sample size** | N (Dyad) = 86  N( Interval 1) = 84  N (Interval 2) = 79 | N (Dyad) = 90  N( Interval 1) = 141  N (Interval 2) = 135 | N (Dyad) = 91  N( Interval 1) = 139  N (Interval 2) = 132 | N (Dyad) = 88  N( Interval 1) = 146  N (Interval 2) = 141 | N (Dyad) = 70  N( Interval 1) = 65  N (Interval 2) = 64 | N (Dyad) = 81  N( Interval 1) = 124  N (Interval 2) = 122 | N (Dyad) = 82  N( Interval 1) = 122  N (Interval 2) = 122 | N (Dyad) = 62  N( Interval 1) = 58  N (Interval 2) = 57 | N (Dyad) = 70  N( Interval 1) = 112  N (Interval 2) = 109 | N (Dyad) = 60  N( Interval 1) = 56  N (Interval 2) = 54 |
| **Intercept** | 0.26 [0.23, 0.30] | 0.28 [0.25, 0.31] | 0.26 [0.23, 0.29] | 0.27 [0.24, 0.30] | 0.26 [0.22, 0.30] | 0.27 [0.24, 0.30] | 0.26 [0.23, 0.29] | 0.27 [0.23, 0.32] | 0.27 [0.24, 0.30] | 0.29 [0.25, 0.34] |
| **Laughter Manipulation** | -0.00 [-0.04, 0.04] | 0.00 [-0.03, 0.03] | 0.00 [-0.03, 0.03] | 0.00 [-0.03, 0.04] | 0.00 [-0.04, 0.05] | 0.00 [-0.03, 0.04] | -0.00 [-0.04, 0.03] | 0.01 [-0.03, 0.06] | -0.00 [-0.04, 0.03] | -0.00 [-0.05, 0.04] |
| **Social** | 0.00 [-0.03, 0.04] | -0.01 [-0.05, 0.02] | 0.00 [-0.03, 0.04] | -0.00 [-0.04, 0.03] | -0.01 [-0.06, 0.03] | -0.00 [-0.04, 0.03] | 0.00 [-0.03, 0.04] | -0.01 [-0.06, 0.03] | 0.00 [-0.03, 0.04] | -0.01 [-0.06, 0.03] |
| **LBMP** | 0.00 [-0.09, 0.10] | -0.00 [-0.09, 0.09] | -0.00 [-0.09, 0.09] | 0.00 [-0.09, 0.10] | 0.00 [-0.09, 0.09] | 0.00 [-0.10, 0.10] | 0.00 [-0.09, 0.09] | -0.00 [-0.09, 0.09] | -0.00 [-0.09, 0.09] | -0.00 [-0.09, 0.09] |
| **LBFI** | -0.00 [-0.09, 0.09] | -0.00 [-0.09, 0.09] | 0.00 [-0.09, 0.09] | 0.00 [-0.09, 0.09] | -0.00 [-0.09, 0.09] | -0.00 [-0.09, 0.09] | -0.00 [-0.09, 0.09] | -0.00 [-0.09, 0.09] | 0.00 [-0.09, 0.09] | -0.00 [-0.09, 0.09] |
| **Interval** | -0.00 [-0.04, 0.04] | -0.00 [-0.04, 0.03] | -0.00 [-0.04, 0.03] | -0.00 [-0.04, 0.02] | 0.00 [-0.04, 0.04] | 0.00 [-0.03, 0.04] | -0.00 [-0.04, 0.03] | -0.00 [-0.05, 0.04] | -0.00 [-0.04, 0.03] | -0.00 [-0.05, 0.04] |
| **Laughter Manipulation:Social** | -0.00 [-0.06, 0.05] | -0.00 [-0.05, 0.04] | -0.01 [-0.06, 0.03] | -0.00 [-0.05, 0.04] | 0.01 [-0.04, 0.07] | 0.00 [-0.04, 0.05] | 0.00 [-0.04, 0.05] | -0.00 [-0.06, 0.05] | 0.01 [-0.04, 0.06] | 0.01 [-0.04, 0.07] |
| **Laughter Manipulation:Interval** | -0.00 [-0.06, 0.05] | -0.00 [-0.05, 0.04] | 0.01 [-0.03, 0.06] | -0.00 [-0.05, 0.04] | -0.00 [-0.06, 0.05] | -0.00 [-0.05, 0.05] | 0.03 [-0.02, 0.08] | -0.00 [-0.06, 0.06] | 0.00 [-0.04, 0.05] | 0.00 [-0.05, 0.06] |
| **Social:Interval** | 0.02 [-0.03, 0.07] | 0.00 [-0.04, 0.05] | 0.02 [-0.01, 0.07] | 0.02 [-0.02, 0.06] | 0.01 [-0.04, 0.07] | 0.00 [-0.04, 0.05] | 0.00 [-0.04, 0.05] | 0.01 [-0.04, 0.07] | 0.01 [-0.03, 0.06] | -0.00 [-0.06, 0.05] |
| **Laughter Manipulation:LBMP** | -0.00 [-0.09, 0.09] | 0.00 [-0.09, 0.09] | 0.00 [-0.09, 0.09] | 0.00 [-0.10, 0.09] | -0.00 [-0.09, 0.09] | 0.00 [-0.09, 0.09] | -0.00 [-0.09, 0.09] | 0.00 [-0.09, 0.09] | -0.00 [-0.09, 0.09] | -0.00 [-0.09, 0.09] |
| **Social:LBMP** | 0.00 [-0.09, 0.09] | 0.00 [-0.09, 0.09] | -0.00 [-0.10, 0.09] | 0.00 [-0.09, 0.09] | -0.00 [-0.09, 0.09] | 0.00 [-0.09, 0.09] | -0.00 [-0.09, 0.09] | -0.00 [-0.09, 0.09] | 0.00 [-0.09, 0.09] | 0.00 [-0.09, 0.09] |
| **Interval:LBMP** | -0.00 [-0.09, 0.09] | -0.00 [-0.09, 0.09] | -0.00 [-0.09, 0.09] | 0.00 [-0.09, 0.09] | -0.00 [-0.09, 0.09] | 0.00 [-0.09, 0.09] | 0.00 [-0.09, 0.09] | 0.00 [-0.09, 0.09] | 0.00 [-0.09, 0.09] | 0.00 [-0.09, 0.09] |
| **Laughter Manipulation:LBFI** | -0.00 [-0.09, 0.09] | -0.00 [-0.09, 0.09] | 0.00 [-0.09, 0.09] | -0.00 [-0.09, 0.09] | -0.00 [-0.09, 0.09] | 0.00 [-0.09, 0.09] | 0.00 [-0.09, 0.09] | 0.00 [-0.09, 0.09] | -0.00 [-0.09, 0.09] | -0.00 [-0.10, 0.09] |
| **Social:LBFI** | -0.00 [-0.09, 0.09] | 0.00 [-0.09, 0.09] | 0.00 [-0.09, 0.09] | 0.00 [-0.09, 0.09] | 0.00 [-0.09, 0.09] | 0.00 [-0.09, 0.10] | 0.00 [-0.09, 0.09] | -0.00 [-0.09, 0.09] | -0.00 [-0.09, 0.09] | -0.00 [-0.09, 0.09] |
| **Interval:LBFI** | 0.00 [-0.09, 0.09] | 0.00 [-0.09, 0.09] | 0.00 [-0.09, 0.09] | 0.00 [-0.09, 0.09] | 0.00 [-0.09, 0.09] | -0.00 [-0.09, 0.09] | -0.00 [-0.09, 0.09] | 0.00 [-0.09, 0.09] | 0.00 [-0.09, 0.09] | -0.00 [-0.09, 0.09] |
| **LBMP:LBFI** | 0.00 [-0.09, 0.09] | 0.00 [-0.09, 0.09] | -0.00 [-0.09, 0.09] | 0.00 [-0.09, 0.09] | 0.00 [-0.09, 0.09] | -0.00 [-0.09, 0.09] | 0.00 [-0.09, 0.09] | -0.00 [-0.09, 0.09] | 0.00 [-0.09, 0.09] | -0.00 [-0.09, 0.09] |
| **Laughter Manipulation:Social:Interval** | 0.02 [-0.04, 0.09] | 0.01 [-0.05, 0.07] | -0.00 [-0.06, 0.05] | 0.01 [-0.04, 0.07] | -0.00 [-0.07, 0.06] | 0.00 [-0.06, 0.06] | -0.00 [-0.07, 0.05] | -0.00 [-0.08, 0.06] | -0.00 [-0.07, 0.05] | -0.00 [-0.08, 0.06] |
| **Laughter Manipulation:Social:LBMP** | 0.00 [-0.09, 0.09] | -0.00 [-0.10, 0.10] | 0.00 [-0.09, 0.10] | -0.00 [-0.09, 0.09] | 0.00 [-0.09, 0.09] | -0.00 [-0.10, 0.09] | 0.00 [-0.09, 0.09] | 0.00 [-0.09, 0.09] | 0.00 [-0.09, 0.09] | -0.00 [-0.09, 0.09] |
| **Laughter Manipulation:Interval:LBMP** | 0.00 [-0.09, 0.09] | 0.00 [-0.09, 0.09] | 0.00 [-0.09, 0.10] | 0.00 [-0.09, 0.09] | -0.00 [-0.09, 0.09] | 0.00 [-0.09, 0.09] | -0.00 [-0.09, 0.10] | 0.00 [-0.09, 0.09] | 0.00 [-0.09, 0.09] | -0.00 [-0.09, 0.09] |
| **Social:Interval:LBMP** | 0.00 [-0.09, 0.09] | 0.00 [-0.09, 0.09] | -0.00 [-0.09, 0.09] | 0.00 [-0.09, 0.09] | 0.00 [-0.09, 0.09] | 0.00 [-0.09, 0.09] | -0.00 [-0.09, 0.09] | -0.00 [-0.09, 0.09] | -0.00 [-0.10, 0.09] | -0.00 [-0.09, 0.09] |
| **Laughter Manipulation:Social:LBFI** | 0.00 [-0.09, 0.09] | 0.00 [-0.09, 0.09] | 0.00 [-0.09, 0.09] | 0.00 [-0.09, 0.09] | 0.00 [-0.09, 0.10] | 0.00 [-0.09, 0.09] | -0.00 [-0.09, 0.09] | -0.00 [-0.09, 0.09] | 0.00 [-0.09, 0.09] | 0.00 [-0.09, 0.09] |
| **Laughter Manipulation:Interval:LBFI** | -0.00 [-0.09, 0.09] | -0.00 [-0.09, 0.09] | -0.00 [-0.09, 0.09] | 0.00 [-0.09, 0.09] | -0.00 [-0.09, 0.09] | -0.00 [-0.09, 0.09] | 0.00 [-0.09, 0.09] | 0.00 [-0.09, 0.09] | 0.00 [-0.09, 0.09] | 0.00 [-0.09, 0.09] |
| **Social:Interval:LBFI** | -0.00 [-0.09, 0.09] | 0.00 [-0.09, 0.09] | 0.00 [-0.10, 0.09] | -0.00 [-0.09, 0.09] | 0.00 [-0.09, 0.09] | 0.00 [-0.09, 0.10] | -0.00 [-0.09, 0.09] | 0.00 [-0.09, 0.09] | -0.00 [-0.09, 0.09] | 0.00 [-0.09, 0.09] |
| **Laughter Manipulation:LBMP:LBFI** | -0.00 [-0.09, 0.09] | -0.00 [-0.09, 0.09] | 0.00 [-0.09, 0.09] | -0.00 [-0.09, 0.09] | -0.00 [-0.09, 0.09] | -0.00 [-0.09, 0.09] | 0.00 [-0.09, 0.09] | -0.00 [-0.09, 0.09] | -0.00 [-0.10, 0.09] | 0.00 [-0.10, 0.09] |
| **Social:LBMP:LBFI** | 0.00 [-0.09, 0.09] | 0.00 [-0.09, 0.09] | 0.00 [-0.09, 0.09] | 0.00 [-0.09, 0.10] | -0.00 [-0.09, 0.09] | 0.00 [-0.09, 0.09] | 0.00 [-0.09, 0.09] | 0.00 [-0.09, 0.09] | -0.00 [-0.09, 0.09] | -0.00 [-0.09, 0.09] |
| **Interval:LBMP:LBFI** | -0.00 [-0.09, 0.09] | -0.00 [-0.09, 0.09] | -0.00 [-0.09, 0.09] | 0.00 [-0.09, 0.09] | -0.00 [-0.09, 0.09] | -0.00 [-0.09, 0.09] | 0.00 [-0.09, 0.09] | 0.00 [-0.09, 0.09] | -0.00 [-0.09, 0.09] | 0.00 [-0.09, 0.09] |
| **Laughter Manipulation:Social:Interval:LBMP** | 0.00 [-0.09, 0.09] | -0.00 [-0.09, 0.09] | -0.00 [-0.09, 0.09] | -0.00 [-0.09, 0.09] | 0.00 [-0.09, 0.10] | 0.00 [-0.09, 0.09] | 0.00 [-0.09, 0.09] | -0.00 [-0.10, 0.09] | 0.00 [-0.09, 0.09] | -0.00 [-0.09, 0.09] |
| **Laughter Manipulation:Social:Interval:LBFI** | -0.00 [-0.09, 0.09] | -0.00 [-0.10, 0.09] | 0.00 [-0.09, 0.09] | -0.00 [-0.09, 0.09] | 0.00 [-0.09, 0.09] | 0.00 [-0.09, 0.09] | -0.00 [-0.09, 0.09] | 0.00 [-0.09, 0.09] | -0.00 [-0.09, 0.09] | 0.00 [-0.09, 0.10] |
| **Laughter Manipulation:Social:LBMP:LBFI** | -0.00 [-0.10, 0.09] | 0.00 [-0.09, 0.09] | 0.00 [-0.09, 0.10] | 0.00 [-0.09, 0.09] | 0.00 [-0.09, 0.09] | -0.00 [-0.10, 0.09] | -0.00 [-0.09, 0.09] | -0.00 [-0.09, 0.09] | 0.00 [-0.09, 0.09] | 0.00 [-0.09, 0.09] |
| **Laughter Manipulation:Interval:LBMP:LBFI** | 0.00 [-0.09, 0.09] | -0.00 [-0.09, 0.09] | 0.00 [-0.09, 0.09] | 0.00 [-0.09, 0.09] | 0.00 [-0.10, 0.10] | -0.00 [-0.10, 0.10] | -0.00 [-0.09, 0.09] | 0.00 [-0.09, 0.09] | 0.00 [-0.09, 0.09] | 0.00 [-0.09, 0.09] |
| **Social:Interval:LBMP:LBFI** | -0.00 [-0.10, 0.09] | 0.00 [-0.09, 0.09] | 0.00 [-0.09, 0.09] | -0.00 [-0.09, 0.09] | -0.00 [-0.09, 0.09] | -0.00 [-0.09, 0.09] | 0.00 [-0.09, 0.09] | -0.00 [-0.09, 0.09] | -0.00 [-0.09, 0.09] | -0.00 [-0.09, 0.09] |
| **Laughter Manipulation:Social:Interval:LBMP:LBFI** | -0.00 [-0.09, 0.09] | 0.00 [-0.09, 0.09] | 0.00 [-0.09, 0.09] | 0.00 [-0.09, 0.09] | 0.00 [-0.09, 0.09] | 0.00 [-0.09, 0.09] | -0.00 [-0.09, 0.09] | 0.00 [-0.09, 0.10] | 0.00 [-0.09, 0.09] | -0.00 [-0.09, 0.09] |

Table 4. Posterior Estimates for all Effects from the Bayesian parameter estimations by ROI Pair for synchrony measured during the free interaction phase. Posterior means and 95% credible intervals (CI) are reported for all main effects and interactions separately for each ROI pair. The analyses were conducted for 10 ROI pairs, which include the inferior frontal gyrus (IFG) and the temporoparietal junction (TPJ) in both hemispheres, labeled as left (l) and right (r). Laughter behavior during the manipulation phase (LBMP) and laughter behavior during the free interaction phase (FI). Sample sizes for Interval 1 and Interval 2 refer to the number of WTC values; each dyad contributes one measure per interval, resulting in two per dyad overall. For heterogeneous ROI pairs, sample sizes are higher than for homologous pairs because each pair includes bidirectional synchrony (e.g., TPJl-IFGr and IFGr-TPJl), both of which are included together for one ROI-pair analysis.

| ROI | H1a_full vs H1a_null_manipulation | H1a_full vs H1a_null_LBMP | H1b_full vs H1a_full |
| --- | --- | --- | --- |
| IFGl_IFGl | 0.43 | 0.00 | 0.16 |
| IFGl_TPJl | 0.25 | 0.00 | 0.12 |
| IFGl_TPJr | 0.23 | 0.00 | 0.11 |
| IFGr_IFGl | 0.72 | 0.00 | 0.08 |
| IFGr_IFGr | 0.53 | 0.00 | 0.18 |
| IFGr_TPJl | 0.41 | 0.00 | 0.14 |
| IFGr_TPJr | 0.31 | 0.00 | 0.11 |
| TPJl_TPJl | 0.39 | 0.00 | 0.27 |
| TPJr_TPJl | 0.18 | 0.00 | 0.40 |
| TPJr_TPJr | 0.42 | 0.00 | 0.31 |

Table 5. This table reports Bayes factor comparisons for model comparisons testing Hypotheses 1a and 1b for synchrony during manipulation phase. The H1b_full model includes all predictors and interactions (*Synchrony ∼ Laughter Manipulation*Social*Interval* LBMP (Laughter Behavior during Manipulation Phase)*. The H1a_full model excludes the key interaction term between Laughter (Manipulation, LBMP) and Social, focusing instead on main effects and simpler interactions.

| ROI | H1a_full vs H1a_null_manipulation | H1a_full vs H1a_null_behavior_mp | H1a_full vs H1a_null_behavior_fi | H1b_full vs H1a_full |
| --- | --- | --- | --- | --- |
| IFGl_IFGl | 0.39 | 0.00 | 0.02 | 0.23 |
| IFGl_TPJl | 0.30 | 0.00 | 0.00 | 0.12 |
| IFGl_TPJr | 0.34 | 0.00 | 0.00 | 0.13 |
| IFGr_IFGl | 0.28 | 0.00 | 0.01 | 0.14 |
| IFGr_IFGr | 0.58 | 0.00 | 0.23 | 0.26 |
| IFGr_TPJl | 0.40 | 0.00 | 0.00 | 0.13 |
| IFGr_TPJr | 0.96 | 0.00 | 0.00 | 0.13 |
| TPJl_TPJl | 0.52 | 0.00 | 0.01 | 0.25 |
| TPJr_TPJl | 0.32 | 0.00 | 0.00 | 0.16 |
| TPJr_TPJr | 0.45 | 0.00 | 0.01 | 0.26 |

Table 6. This table reports Bayes factor comparisons for model comparisons testing Hypotheses 1a and 1b for synchrony during the free interaction phase. The H1b_full model includes all predictors and interactions (*Synchrony ∼ Laughter Manipulation*Social*Interval* LBMP (Laughter Behavior during Manipulation Phase)*LBFI (Laughter Behavior during Free Interaction).* H1a_full model excludes the key interaction term between Laughter (Manipulation, LBMP, LBFI) and Social, focusing instead on main effects and simpler interactions. Additionally, H1a_null_manipulation excludes Laughter Manipulation, H1a_null_LBMP excludes LBMP, and H1a_null_LBFI excludes LBFI to evaluate the contribution of these predictors to the full model. The Bayes factors quantify the relative evidence for the full models compared to the reduced models.

| Parameter | Liking | Bonding | Prosociality (Helping) | Prosociality (Money Sharing) |
| --- | --- | --- | --- | --- |
| Intercept | 6.34 [6.12, 6.56] | 2.56 [2.31, 2.82] | 0.84 [0.77, 0.90] | 0.61 [0.54, 0.68] |
| Laughter Manipulation | -0.03 [-0.33, 0.26] | -0.05 [-0.38, 0.29] | -0.01 [-0.11, 0.09] | -0.08 [-0.19, 0.03] |
| Social | -0.08 [-0.36, 0.21] | 0.28 [-0.05, 0.60] | 0.07 [-0.03, 0.16] | 0.03 [-0.08, 0.13] |
| LBMP | 0.00 [-0.59, 0.61] | -0.00 [-0.59, 0.59] | 0.00 [-0.58, 0.59] | -0.00 [-0.59, 0.58] |
| LBFI | -0.00 [-0.59, 0.59] | -0.00 [-0.59, 0.58] | 0.00 [-0.57, 0.59] | 0.00 [-0.57, 0.58] |
| Laughter Manipulation:Social | -0.12 [-0.49, 0.26] | -0.24 [-0.64, 0.18] | -0.02 [-0.16, 0.12] | 0.05 [-0.10, 0.20] |
| Laughter Manipulation:LBMP | 0.00 [-0.58, 0.58] | -0.00 [-0.58, 0.59] | 0.00 [-0.58, 0.60] | 0.00 [-0.58, 0.59] |
| Social:LBMP | 0.00 [-0.59, 0.58] | -0.00 [-0.58, 0.57] | -0.00 [-0.60, 0.59] | 0.00 [-0.59, 0.60] |
| Laughter Manipulation:LBFI | -0.00 [-0.58, 0.58] | -0.00 [-0.58, 0.58] | 0.00 [-0.57, 0.60] | 0.00 [-0.59, 0.60] |
| Social:LBFI | -0.00 [-0.59, 0.58] | -0.01 [-0.58, 0.58] | 0.00 [-0.58, 0.59] | 0.00 [-0.59, 0.59] |
| LBMP:LBFI | -0.00 [-0.58, 0.58] | -0.00 [-0.57, 0.56] | -0.00 [-0.59, 0.58] | -0.00 [-0.58, 0.60] |
| Laughter Manipulation:Social: LBMP | -0.00 [-0.58, 0.58] | -0.01 [-0.59, 0.59] | -0.00 [-0.60, 0.60] | -0.00 [-0.58, 0.58] |
| Laughter Manipulation:Social: LBFI | -0.00 [-0.58, 0.57] | -0.00 [-0.59, 0.58] | -0.00 [-0.57, 0.58] | 0.00 [-0.58, 0.60] |
| Laughter Manipulation:LBMP: LBFI | -0.00 [-0.59, 0.59] | 0.00 [-0.59, 0.59] | -0.00 [-0.58, 0.60] | 0.00 [-0.58, 0.58] |
| Social:LBMP:LBFI | 0.00 [-0.58, 0.59] | 0.00 [-0.58, 0.58] | 0.00 [-0.57, 0.59] | -0.00 [-0.57, 0.58] |
| Laughter Manipulation:Social: LBMP:LBFI | -0.00 [-0.58, 0.58] | -0.01 [-0.59, 0.57] | 0.00 [-0.59, 0.58] | -0.00 [-0.58, 0.57] |
| Liking_1 | 0.70 [0.60, 0.81] |  |  |  |

Table 7. This table reports the posterior estimates and 95% credible intervals (CI) from the Bayesian parameter estimation analyses for Hypothesis 2, which examines the effects of Laughter on social outcome variables (Liking, Bonding, and Prosociality (Helping and Money Sharing). The predictors include Laughter Manipulation (yes/no), Social Context (yes/no), LBMP (Laughter Behavior during the manipulation phase), and LBFI (Laughter Behavior during the free interaction phase). The posterior estimates provide information on the strength and direction of the effects of these predictors and their interactions. Liking_1 was measured before the experiment and included as a covariate in the model predicting the outcome on Liking (measured after the free interaction phase).

| Outcome | H2a_full vs H2a_null_manipulation | H2a_full vs H2a_null_LBMP | H2a_full vs H2a_null_LBFI | H2b_full vs H2a_full |
| --- | --- | --- | --- | --- |
| Liking | 0.51 | 20745691772.16 | 157.54 | 0.76 |
| Prosociality (Money Sharing) | 0.34 | 23.25 | 23.84 | 0.32 |
| Prosociality (Helping) | 0.13 | 0.01 | 0.61 | 0.24 |
| Bonding | 0.79 | 8267849351869.52 | 544.31 | 1.35 |

Table 8. This table reports Bayes factor comparisons for model comparisons testing Hypothesis 2 (Laughter increases social outcomes). The H2b_full model (*outcome variable ∼ Laughter Manipulation * Social * LBMP * LBFI + (1| Dyad)*) includes all predictors and their interactions. This model was compared to the H2a_full model *(outcome variable ~ Laughter Manipulation + Social + LBMP + LBFI + (1| Dyad))*, which represents a simpler model without the main interaction terms. Additionally, the H2a_full model was compared to reduced models to assess the contribution of individual predictors: H2a_null_manipulation excludes the predictor Laughter Manipulation (yes/no). H2a_null_LBMP excludes the predictor LBMP (laughter behavior during the manipulation phase). H2a_null_LBFI excludes the predictor LBFI (laughter behavior during the free interaction phase).

| Treatment: Condition  Outcome: Liking | | | | | |
| --- | --- | --- | --- | --- | --- |
| ROI Pair | Direct Effect | Indirect Effect | Mediator Effect | Total Effect | Proportion Mediated |
| IFGl_IFGl | -0.35[-1.17, 0.46] | 0.02[-0.08, 0.21] | -4.50[-16.55, 7.03] | -0.31[-1.14, 0.48] | -6.99[-207.62, 193.65] |
| IFGl_TPJl | -0.29[-1.11, 0.48] | -0.04[-0.27, 0.08] | -5.73[-18.83, 7.13] | -0.36[-1.17, 0.43] | 11.91[-173.75, 197.56] |
| IFGl_TPJr | -0.39[-1.18, 0.38] | 0.01[-0.11, 0.18] | -9.58[-23.09, 4.09] | -0.36[-1.17, 0.41] | -2.83[-133.55, 127.85] |
| IFGr_IFGl | -0.34[-1.11, 0.46] | 0.00[-0.18, 0.19] | -0.15[-11.36, 10.86] | -0.35[-1.11, 0.48] | -0.19[-171.65, 171.28] |
| IFGr_IFGr | -0.23[-1.06, 0.61] | -0.00[-0.13, 0.10] | 1.08[-7.37, 9.72] | -0.23[-1.07, 0.60] | 1.01[-113.57, 115.59] |
| IFGr_TPJl | -0.21[-1.08, 0.62] | 0.01[-0.16, 0.23] | 1.73[-10.08, 13.35] | -0.19[-1.04, 0.62] | -8.44[-236.16, 219.27] |
| IFGr_TPJr | -0.13[-0.92, 0.65] | -0.04[-0.28, 0.10] | -10.19[-21.30, 0.88] | -0.18[-0.99, 0.60] | 22.42[-257.57, 302.41] |
| TPJl_TPJl | -0.16[-1.08, 0.81] | -0.00[-0.22, 0.14] | -1.76[-12.13, 8.20] | -0.18[-1.11, 0.81] | 3.14[-148.46, 154.75] |
| TPJr_TPJl | 0.06[-0.85, 0.97] | -0.02[-0.27, 0.13] | -3.21[-14.30, 7.73] | 0.02[-0.90, 0.92] | -113.82[-337.37, 109.73] |
| TPJr_TPJr | 0.18[-0.69, 1.06] | -0.00[-0.21, 0.15] | -5.29[-14.41, 3.98] | 0.16[-0.74, 1.04] | -4.04[-202.83, 194.75] |
| Treatment: Laughter Behavior during Manipulation phase  Outcome: Liking | | | | | |
| ROI Pair | Direct Effect | Indirect Effect | Mediator Effect | Total Effect | Proportion Mediated |
| IFGl_IFGl | 4098.65[-3676.43, 11969.14] | -85.19[-1730.24, 828.19] | -3.70[-16.75, 9.42] | 3959.03[-3863.92, 11737.31] | -2.14[-112.18, 107.87] |
| IFGl_TPJl | 3877.27[-3327.71, 11383.62] | -110.80[-2108.90, 1310.90] | -9.80[-24.17, 4.51] | 3722.19[-3931.47, 11195.17] | -2.97[-170.99, 165.03] |
| IFGl_TPJr | 3465.72[-3885.79, 10930.02] | 72.05[-1217.64, 1933.33] | -9.40[-23.64, 4.69] | 3649.78[-3783.01, 11154.82] | 1.96[-150.58, 154.53] |
| IFGr_IFGl | 3790.63[-3818.70, 11447.27] | 1.18[-983.31, 1018.96] | -0.59[-13.28, 12.12] | 3796.32[-3820.14, 11516.59] | 0.02[-74.81, 74.87] |
| IFGr_IFGr | 3224.32[-4957.61, 11605.81] | -14.43[-1626.96, 1428.59] | -0.54[-9.02, 8.33] | 3211.61[-4996.90, 11568.47] | -0.45[-126.90, 126.01] |
| IFGr_TPJl | 3461.96[-4804.14, 12307.63] | -195.86[-2529.80, 1660.92] | -2.31[-14.73, 10.58] | 3125.23[-4919.84, 11944.94] | -6.26[-228.58, 216.03] |
| IFGr_TPJr | 5549.07[-2127.27, 13160.57] | -1359.92[-4423.31, 289.09] | -13.99[-27.25, -0.72] | 4013.77[-3727.73, 11826.17] | -33.88[-452.81, 385.05] |
| TPJl_TPJl | 3887.86[-6734.29, 14792.72] | 315.24[-1505.52, 3470.28] | -5.94[-16.96, 5.50] | 4472.91[-6405.21, 15322.85] | 7.04[-151.55, 165.65] |
| TPJr_TPJl | 5236.43[-3188.95, 13607.36] | 88.18[-1173.16, 2179.48] | -4.91[-16.02, 6.06] | 5450.58[-3135.65, 14069.97] | 1.62[-73.04, 76.26] |
| TPJr_TPJr | 5724.70[-2236.97, 13657.40] | 429.72[-993.94, 3155.37] | -5.89[-15.46, 3.54] | 6403.66[-1590.57, 14141.58] | 6.70[-80.43, 93.85] |
| Treatment: Laughter Behavior during Free Interaction phase  Outcome: Liking | | | | | |
| ROI Pair | Direct Effect | Indirect Effect | Mediator Effect | Total Effect | Proportion Mediated |
| IFGl_IFGl | 13071.05[5194.11, 21069.97] | -16.57[-1555.05, 1419.08] | 0.62[-10.77, 11.97] | 13059.46[5168.27, 21061.79] | -0.13[-14.18, 13.92] |
| IFGl_TPJl | 13088.64[5360.81, 20802.29] | -57.16[-1814.14, 1188.25] | -7.68[-20.07, 4.01] | 12969.85[5032.69, 20737.39] | -0.44[-15.10, 14.22] |
| IFGl_TPJr | 12993.28[5236.10, 20678.95] | -65.05[-1860.35, 1498.41] | -10.00[-23.67, 2.78] | 12892.88[4869.47, 20664.28] | -0.50[-17.28, 16.26] |
| IFGr_IFGl | 12955.14[5301.02, 21126.50] | -14.29[-1200.08, 934.64] | 1.70[-9.13, 12.28] | 12944.82[5262.99, 21143.19] | -0.11[-9.75, 9.52] |
| IFGr_IFGr | 14072.53[6063.27, 21929.40] | -9.02[-1163.84, 1046.07] | 1.19[-7.10, 9.45] | 14014.87[6086.11, 21996.29] | -0.05[-9.11, 8.99] |
| IFGr_TPJl | 14185.93[6097.46, 22264.39] | -122.78[-1959.06, 936.03] | 3.43[-8.19, 14.95] | 13970.70[5818.66, 22097.32] | -0.88[-13.44, 11.68] |
| IFGr_TPJr | 13020.51[5656.87, 20394.80] | 170.95[-1468.00, 2260.63] | -10.26[-21.75, 0.81] | 13271.90[5732.87, 21110.58] | 1.29[-14.84, 17.42] |
| TPJl_TPJl | 15019.72[6572.61, 23129.23] | 10.00[-1155.51, 1537.34] | -1.69[-10.56, 7.10] | 15032.33[6439.21, 23256.08] | 0.07[-10.33, 10.46] |
| TPJr_TPJl | 12851.59[4577.68, 21016.98] | 23.47[-1469.74, 1757.84] | -0.66[-10.79, 9.75] | 12881.71[4591.64, 20991.19] | 0.17[-15.39, 15.75] |
| TPJr_TPJr | 10105.61[2231.29, 17850.61] | 190.18[-1841.70, 2814.49] | -1.33[-10.67, 7.97] | 10376.24[2671.78, 18025.85] | 1.83[-29.37, 33.03] |
| Treatment: Condition  Outcome: Prosociality (Money Sharing) | | | | | |
| ROI Pair | Direct Effect | Indirect Effect | Mediator Effect | Total Effect | Proportion Mediated |
| IFGl_IFGl | -0.07[-0.23, 0.08] | -0.00[-0.03, 0.02] | 0.28[-2.09, 2.69] | -0.07[-0.23, 0.08] | 1.19[-110.18, 112.57] |
| IFGl_TPJl | -0.07[-0.22, 0.08] | -0.00[-0.04, 0.02] | -0.46[-3.13, 2.07] | -0.07[-0.22, 0.07] | 3.62[-147.24, 154.47] |
| IFGl_TPJr | -0.07[-0.23, 0.08] | 0.00[-0.02, 0.03] | -1.57[-4.10, 1.00] | -0.07[-0.23, 0.09] | -1.86[-128.19, 124.48] |
| IFGr_IFGl | -0.06[-0.22, 0.08] | -0.01[-0.06, 0.01] | 1.08[-1.10, 3.27] | -0.08[-0.23, 0.07] | 14.14[-179.80, 208.08] |
| IFGr_IFGr | -0.08[-0.25, 0.07] | -0.00[-0.02, 0.02] | 0.39[-1.33, 2.13] | -0.09[-0.26, 0.07] | 0.81[-70.34, 71.95] |
| IFGr_TPJl | -0.08[-0.24, 0.08] | -0.00[-0.05, 0.02] | -0.63[-2.93, 1.76] | -0.09[-0.25, 0.07] | 6.83[-152.94, 166.62] |
| IFGr_TPJr | -0.07[-0.23, 0.08] | -0.00[-0.04, 0.01] | -1.14[-3.58, 1.15] | -0.08[-0.24, 0.07] | 4.04[-98.78, 106.85] |
| TPJl_TPJl | -0.10[-0.30, 0.09] | -0.00[-0.03, 0.02] | -0.14[-2.08, 1.82] | -0.10[-0.30, 0.09] | 0.46[-85.84, 86.76] |
| TPJr_TPJl | -0.08[-0.26, 0.09] | 0.00[-0.02, 0.05] | 0.73[-1.57, 3.03] | -0.08[-0.25, 0.10] | -7.84[-200.90, 185.21] |
| TPJr_TPJr | -0.09[-0.29, 0.10] | -0.00[-0.02, 0.02] | -0.23[-2.39, 1.92] | -0.09[-0.29, 0.10] | 0.02[-76.54, 76.59] |
| Treatment: Laughter Behavior during Manipulation phase  Outcome: Prosociality (Money Sharing) | | | | | |
| ROI Pair | Direct Effect | Indirect Effect | Mediator Effect | Total Effect | Proportion Mediated |
| IFGl_IFGl | 902.79[-558.60, 2426.51] | 10.33[-196.36, 299.09] | 0.40[-2.17, 2.88] | 927.87[-553.75, 2446.50] | 1.11[-68.39, 70.62] |
| IFGl_TPJl | 960.39[-508.61, 2381.21] | -8.51[-289.87, 189.84] | -1.02[-3.60, 1.62] | 941.72[-551.63, 2371.50] | -0.90[-81.85, 80.04] |
| IFGl_TPJr | 913.32[-528.51, 2358.20] | 10.47[-205.80, 316.99] | -1.37[-4.08, 1.34] | 944.83[-527.21, 2419.68] | 1.11[-76.25, 78.46] |
| IFGr_IFGl | 854.57[-636.11, 2264.59] | -0.70[-260.26, 242.00] | 1.17[-1.21, 3.63] | 854.86[-641.56, 2271.01] | -0.08[-85.15, 84.98] |
| IFGr_IFGr | 577.02[-1064.60, 2107.35] | 1.25[-274.95, 310.04] | 0.06[-1.63, 1.78] | 575.65[-1064.65, 2136.58] | 0.22[-132.80, 133.24] |
| IFGr_TPJl | 663.42[-1002.39, 2267.55] | -89.88[-605.50, 232.25] | -0.91[-3.50, 1.56] | 550.59[-1034.96, 2142.95] | -16.32[-276.99, 244.34] |
| IFGr_TPJr | 925.47[-491.63, 2381.94] | -111.51[-604.45, 148.98] | -1.35[-3.84, 1.13] | 788.97[-637.51, 2232.29] | -14.13[-225.50, 197.24] |
| TPJl_TPJl | 627.84[-1384.63, 2613.27] | 93.45[-286.51, 774.65] | -1.50[-3.76, 0.69] | 763.99[-1241.57, 2832.66] | 12.23[-200.30, 224.77] |
| TPJr_TPJl | 934.17[-775.82, 2584.22] | -0.98[-285.11, 259.75] | 0.10[-2.23, 2.42] | 936.95[-798.45, 2591.26] | -0.11[-88.59, 88.39] |
| TPJr_TPJr | 955.96[-944.42, 2842.92] | 13.44[-327.50, 502.29] | -0.34[-2.69, 1.88] | 995.79[-898.17, 2886.10] | 1.35[-103.37, 106.06] |
| Treatment: Laughter Behavior during Free Interaction phase  Outcome: Prosociality (Money Sharing) | | | | | |
| ROI Pair | Direct Effect | Indirect Effect | Mediator Effect | Total Effect | Proportion Mediated |
| IFGl_IFGl | 1377.12[-87.57, 2936.48] | -44.97[-424.14, 168.21] | 0.79[-1.43, 2.99] | 1299.76[-162.51, 2859.21] | -3.45[-68.75, 61.82] |
| IFGl_TPJl | 1343.11[-196.27, 2866.00] | -2.94[-287.32, 228.97] | -0.85[-3.25, 1.61] | 1332.24[-188.58, 2865.09] | -0.22[-42.14, 41.70] |
| IFGl_TPJr | 1346.58[-228.66, 2839.73] | -5.81[-292.73, 237.58] | -1.42[-3.91, 1.01] | 1329.41[-262.85, 2818.45] | -0.44[-44.74, 43.86] |
| IFGr_IFGl | 1369.31[-142.89, 2841.07] | -31.08[-434.71, 221.78] | 1.55[-0.50, 3.75] | 1309.99[-198.18, 2835.82] | -2.37[-68.92, 64.17] |
| IFGr_IFGr | 1466.14[-42.92, 2952.06] | -13.33[-365.97, 254.69] | 0.93[-0.63, 2.47] | 1433.74[-89.31, 2935.79] | -0.93[-46.67, 44.82] |
| IFGr_TPJl | 1362.74[-195.09, 2924.28] | 17.89[-197.38, 373.14] | -0.53[-2.82, 1.78] | 1395.48[-200.46, 2956.53] | 1.28[-36.38, 38.93] |
| IFGr_TPJr | 1250.81[-369.93, 2806.10] | 22.55[-283.37, 396.12] | -1.72[-4.00, 0.40] | 1290.31[-353.12, 2869.49] | 1.75[-54.96, 58.46] |
| TPJl_TPJl | 1271.38[-441.44, 2930.14] | 3.57[-223.76, 304.24] | -0.34[-2.11, 1.45] | 1280.74[-459.05, 2912.57] | 0.28[-49.14, 49.71] |
| TPJr_TPJl | 1376.26[-309.13, 2985.66] | -80.91[-600.28, 181.22] | 1.09[-1.06, 3.23] | 1235.47[-436.75, 2940.13] | -6.54[-119.28, 106.18] |
| TPJr_TPJr | 1247.96[-671.11, 3158.32] | -103.78[-770.23, 384.35] | 0.63[-1.60, 2.81] | 1101.85[-800.16, 2990.40] | -9.41[-195.80, 176.96] |
| Treatment: Condition  Outcome: Prosociality (Helping) | | | | | |
| ROI Pair | Direct Effect | Indirect Effect | Mediator Effect | Total Effect | Proportion Mediated |
| IFGl_IFGl | -0.00[-0.13, 0.12] | -0.00[-0.02, 0.02] | 0.19[-1.64, 2.08] | -0.01[-0.13, 0.11] | 6.91[-155.43, 169.25] |
| IFGl_TPJl | -0.00[-0.13, 0.12] | -0.01[-0.05, 0.00] | -1.53[-3.59, 0.57] | -0.01[-0.14, 0.11] | 67.29[-336.39, 470.97] |
| IFGl_TPJr | -0.02[-0.15, 0.10] | 0.00[-0.02, 0.02] | -1.55[-3.57, 0.53] | -0.01[-0.15, 0.11] | -8.01[-208.58, 192.53] |
| IFGr_IFGl | 0.01[-0.11, 0.14] | -0.02[-0.07, 0.00] | 1.84[0.12, 3.65] | -0.00[-0.14, 0.11] | 261.68[-406.88, 930.26] |
| IFGr_IFGr | 0.00[-0.13, 0.13] | -0.00[-0.03, 0.01] | 0.80[-0.62, 2.24] | -0.00[-0.13, 0.13] | 55.53[-144.15, 255.21] |
| IFGr_TPJl | -0.00[-0.14, 0.13] | -0.00[-0.04, 0.02] | -0.71[-2.61, 1.27] | -0.01[-0.14, 0.12] | 62.31[-260.25, 384.88] |
| IFGr_TPJr | 0.00[-0.13, 0.13] | -0.00[-0.03, 0.01] | -1.01[-2.97, 0.86] | -0.00[-0.14, 0.13] | 78.15[-135.62, 291.93] |
| TPJl_TPJl | -0.00[-0.15, 0.15] | -0.00[-0.04, 0.01] | -0.61[-2.16, 0.96] | -0.00[-0.16, 0.15] | 52.90[-144.06, 249.90] |
| TPJr_TPJl | 0.00[-0.13, 0.14] | 0.00[-0.02, 0.03] | 0.17[-1.58, 1.92] | 0.01[-0.12, 0.15] | 8.35[-211.58, 228.30] |
| TPJr_TPJr | 0.00[-0.15, 0.16] | 0.00[-0.02, 0.02] | 0.04[-1.68, 1.71] | 0.00[-0.15, 0.16] | 3.99[-124.95, 132.93] |
| Treatment: Laughter Behavior during Manipulation phase  Outcome: Prosociality (Helping) | | | | | |
| ROI Pair | Direct Effect | Indirect Effect | Mediator Effect | Total Effect | Proportion Mediated |
| IFGl_IFGl | 299.92[-927.16, 1555.55] | 2.04[-190.19, 215.64] | 0.07[-1.98, 2.13] | 299.43[-915.23, 1567.81] | 0.68[-141.97, 143.34] |
| IFGl_TPJl | 310.86[-876.40, 1470.09] | -27.67[-356.27, 229.17] | -1.83[-3.84, 0.29] | 263.57[-956.20, 1466.03] | -10.50[-225.12, 204.12] |
| IFGl_TPJr | 222.31[-954.36, 1380.19] | 13.11[-195.76, 309.39] | -1.51[-3.72, 0.69] | 248.02[-939.84, 1448.76] | 5.29[-180.78, 191.34] |
| IFGr_IFGl | 328.27[-868.05, 1515.90] | -2.14[-260.07, 239.79] | 1.43[-0.38, 3.33] | 326.37[-898.48, 1534.61] | -0.66[-169.56, 168.25] |
| IFGr_IFGr | 160.75[-1140.96, 1529.93] | 21.07[-166.55, 341.16] | 0.38[-1.05, 1.77] | 207.21[-1110.02, 1564.12] | 10.16[-164.30, 184.63] |
| IFGr_TPJl | 317.25[-1025.97, 1706.32] | -131.13[-595.66, 140.97] | -1.20[-3.37, 0.93] | 160.74[-1178.82, 1520.35] | -81.57[-551.65, 388.50] |
| IFGr_TPJr | 527.19[-729.92, 1753.99] | -150.58[-615.68, 83.76] | -1.61[-3.74, 0.58] | 344.82[-913.47, 1572.49] | -43.67[-506.04, 418.69] |
| TPJl_TPJl | -35.03[-1673.37, 1581.94] | 65.37[-218.78, 601.46] | -1.11[-2.78, 0.61] | 83.99[-1572.26, 1706.11] | 77.84[-200.37, 356.05] |
| TPJr_TPJl | 437.59[-830.57, 1769.93] | 0.77[-206.74, 234.60] | -0.16[-1.94, 1.71] | 447.79[-837.51, 1797.83] | 0.17[-109.06, 109.42] |
| TPJr_TPJr | 568.05[-879.10, 1990.81] | 1.25[-305.08, 310.43] | -0.05[-1.76, 1.68] | 578.59[-900.02, 2014.13] | 0.22[-131.37, 131.81] |
| Treatment: Laughter Behavior during Free Interaction phase  Outcome: Prosociality (Helping) | | | | | |
| ROI Pair | Direct Effect | Indirect Effect | Mediator Effect | Total Effect | Proportion Mediated |
| IFGl_IFGl | 950.77[-373.26, 2349.97] | -26.69[-340.36, 178.35] | 0.50[-1.38, 2.37] | 900.95[-437.74, 2281.79] | -2.95[-100.68, 94.76] |
| IFGl_TPJl | 927.99[-316.54, 2210.21] | -17.69[-332.07, 248.44] | -1.61[-3.68, 0.38] | 897.59[-371.52, 2199.97] | -1.96[-92.50, 88.56] |
| IFGl_TPJr | 930.35[-381.68, 2232.83] | -8.54[-321.48, 237.39] | -1.63[-3.87, 0.56] | 905.72[-449.75, 2223.09] | -0.93[-96.87, 95.00] |
| IFGr_IFGl | 983.72[-331.12, 2231.02] | -51.14[-479.62, 254.42] | 1.91[0.13, 3.63] | 905.99[-465.12, 2268.35] | -5.65[-153.40, 142.11] |
| IFGr_IFGr | 1169.72[-227.26, 2603.09] | -13.96[-326.11, 231.13] | 0.93[-0.45, 2.40] | 1138.32[-309.23, 2569.83] | -1.22[-69.34, 66.90] |
| IFGr_TPJl | 994.64[-441.08, 2395.08] | 15.98[-171.46, 316.21] | -0.48[-2.50, 1.48] | 1035.21[-404.71, 2476.53] | 1.54[-69.50, 72.59] |
| IFGr_TPJr | 938.54[-449.33, 2310.20] | 14.16[-197.79, 300.94] | -1.24[-3.18, 0.73] | 954.87[-405.78, 2355.25] | 1.47[-67.18, 70.15] |
| TPJl_TPJl | 1062.89[-358.49, 2558.39] | 8.14[-221.06, 309.87] | -0.60[-2.15, 0.95] | 1095.65[-379.12, 2626.32] | 0.73[-63.07, 64.56] |
| TPJr_TPJl | 1086.09[-320.17, 2467.34] | -22.17[-377.19, 210.18] | 0.40[-1.33, 2.22] | 1035.88[-364.71, 2402.94] | -2.14[-85.79, 81.51] |
| TPJr_TPJr | 1491.75[-81.59, 3016.61] | -95.87[-653.30, 275.37] | 0.59[-1.14, 2.37] | 1365.28[-209.52, 2913.81] | -7.01[-95.35, 81.31] |
| Treatment: Condition  Outcome: Bonding | | | | | |
| ROI Pair | Direct Effect | Indirect Effect | Mediator Effect | Total Effect | Proportion Mediated |
| IFGl_IFGl | -0.29[-0.93, 0.34] | 0.04[-0.05, 0.23] | -6.91[-16.12, 2.49] | -0.23[-0.88, 0.41] | -19.46[-306.60, 267.66] |
| IFGl_TPJl | -0.25[-0.90, 0.38] | -0.00[-0.13, 0.12] | -0.73[-10.99, 9.75] | -0.26[-0.90, 0.37] | 1.37[-138.36, 141.09] |
| IFGl_TPJr | -0.27[-0.92, 0.34] | 0.00[-0.08, 0.13] | -5.82[-16.57, 4.79] | -0.26[-0.90, 0.37] | -1.36[-140.08, 137.36] |
| IFGr_IFGl | -0.24[-0.89, 0.41] | 0.00[-0.15, 0.16] | -0.69[-10.28, 8.60] | -0.23[-0.90, 0.42] | -2.06[-201.71, 197.59] |
| IFGr_IFGr | -0.22[-0.90, 0.44] | -0.01[-0.17, 0.08] | 3.77[-3.08, 10.36] | -0.25[-0.93, 0.42] | 4.57[-154.03, 163.16] |
| IFGr_TPJl | -0.14[-0.79, 0.53] | -0.05[-0.27, 0.05] | -5.19[-14.70, 4.19] | -0.21[-0.86, 0.45] | 26.71[-271.37, 324.80] |
| IFGr_TPJr | -0.17[-0.84, 0.50] | -0.03[-0.22, 0.08] | -7.88[-17.10, 1.29] | -0.22[-0.91, 0.47] | 14.28[-199.68, 228.28] |
| TPJl_TPJl | 0.05[-0.77, 0.85] | -0.03[-0.27, 0.10] | -5.30[-13.06, 2.69] | 0.00[-0.82, 0.79] | -653.95[-947.00, -360.89] |
| TPJr_TPJl | -0.05[-0.76, 0.63] | 0.00[-0.15, 0.18] | 0.32[-9.12, 9.85] | -0.04[-0.76, 0.63] | -2.68[-259.86, 254.47] |
| TPJr_TPJr | -0.08[-0.85, 0.66] | -0.00[-0.11, 0.10] | -0.70[-9.18, 7.47] | -0.09[-0.86, 0.66] | 1.02[-108.20, 110.26] |
| Treatment: Laughter Behavior during Manipulation phase  Outcome: Bonding | | | | | |
| ROI Pair | Direct Effect | Indirect Effect | Mediator Effect | Total Effect | Proportion Mediated |
| IFGl_IFGl | 1288.90[-5033.95, 7516.63] | -221.33[-2006.38, 673.43] | -6.68[-17.56, 4.23] | 881.65[-5416.04, 7268.41] | -25.10[-284.97, 234.75] |
| IFGl_TPJl | 833.63[-5749.48, 7142.64] | -16.86[-1193.54, 789.29] | -2.82[-14.45, 8.65] | 727.59[-5870.47, 7103.51] | -2.31[-132.47, 127.84] |
| IFGl_TPJr | 627.09[-5931.18, 7222.54] | 39.19[-988.62, 1329.76] | -5.67[-17.23, 5.84] | 741.82[-5925.31, 7520.07] | 5.28[-150.21, 160.78] |
| IFGr_IFGl | 974.48[-5223.64, 7093.75] | -1.23[-829.64, 757.83] | -0.23[-10.57, 10.12] | 951.14[-5283.36, 7127.13] | -0.13[-110.31, 110.06] |
| IFGr_IFGr | 584.07[-6303.41, 7320.59] | 142.21[-769.74, 1928.12] | 2.67[-4.91, 10.23] | 885.37[-5996.93, 7736.69] | 16.05[-176.58, 208.69] |
| IFGr_TPJl | 2439.18[-4734.88, 9436.09] | -1166.82[-4051.21, 366.28] | -10.26[-21.27, 0.89] | 1104.78[-6055.51, 8060.57] | -105.62[-765.58, 554.35] |
| IFGr_TPJr | 2145.34[-4190.57, 8374.50] | -1111.27[-3626.75, 242.71] | -11.61[-22.79, -0.70] | 873.49[-5653.08, 7318.72] | -127.21[-741.44, 486.99] |
| TPJl_TPJl | 5692.72[-3452.77, 14358.74] | 277.25[-1211.43, 2935.00] | -4.86[-14.01, 3.98] | 6165.02[-3138.11, 15031.27] | 4.50[-86.25, 95.25] |
| TPJr_TPJl | 4222.31[-3124.13, 11469.93] | 3.29[-1086.02, 1076.65] | -0.16[-9.30, 9.00] | 4253.66[-3127.29, 11408.34] | 0.08[-67.07, 67.23] |
| TPJr_TPJr | 4000.41[-3321.20, 11175.89] | 36.35[-1246.61, 1900.04] | -1.19[-9.96, 7.46] | 4101.49[-3181.05, 11314.95] | 0.89[-106.78, 108.54] |
| Treatment: Laughter Behavior during Free Interaction phase  Outcome: Bonding | | | | | |
| ROI Pair | Direct Effect | Indirect Effect | Mediator Effect | Total Effect | Proportion Mediated |
| IFGl_IFGl | 7091.01[682.49, 13520.27] | 250.75[-633.77, 2009.35] | -4.07[-13.36, 5.43] | 7488.86[1084.34, 13861.78] | 3.35[-24.33, 31.03] |
| IFGl_TPJl | 7474.90[1019.13, 13966.74] | -7.07[-912.48, 709.54] | -2.00[-11.55, 8.30] | 7399.96[980.59, 14051.48] | -0.10[-17.71, 17.51] |
| IFGl_TPJr | 7461.40[809.87, 14037.63] | -29.88[-1403.91, 1017.14] | -6.53[-16.86, 3.91] | 7374.08[642.94, 13879.64] | -0.40[-27.33, 26.53] |
| IFGr_IFGl | 7416.38[775.79, 14039.24] | -3.54[-895.78, 807.19] | 0.68[-8.32, 9.39] | 7410.20[736.39, 14062.23] | -0.05[-18.76, 18.67] |
| IFGr_IFGr | 7276.60[484.91, 13826.72] | -46.09[-1502.76, 995.00] | 3.66[-2.93, 10.52] | 7181.55[283.25, 13868.74] | -0.64[-30.71, 29.42] |
| IFGr_TPJl | 7154.51[202.65, 14078.80] | 276.19[-618.45, 2098.08] | -5.70[-15.25, 3.63] | 7509.43[538.45, 14635.67] | 3.68[-26.28, 33.64] |
| IFGr_TPJr | 7484.55[776.63, 14271.00] | 91.41[-1251.27, 1778.17] | -7.88[-17.55, 1.84] | 7634.84[934.85, 14700.06] | 1.19[-28.23, 30.64] |
| TPJl_TPJl | 6911.06[-520.27, 14118.80] | 104.91[-1418.15, 2144.57] | -5.31[-12.65, 2.59] | 7155.37[-480.31, 14469.80] | 1.46[-45.04, 47.97] |
| TPJr_TPJl | 7269.58[483.11, 14319.38] | -70.31[-1758.53, 1123.74] | 1.49[-7.31, 10.47] | 7085.79[194.44, 14017.48] | -0.98[-40.21, 38.22] |
| TPJr_TPJr | 6592.65[-1113.06, 14082.35] | -229.34[-2648.15, 1840.20] | 1.63[-7.18, 10.34] | 6259.55[-1143.78, 13654.64] | -3.66[-109.62, 102.29] |

Table 9. Posterior estimates from Bayesian mediation models for Synchrony during the manipulation phase. Social outcome variables (Liking, Prosociality (Money Sharing, and Helping), and Bonding) by ROI Pair. Separate models were computed for each ROI pair (as the mediator) with either the Laughter Condition (Laughter experience vs. no Laughter experience) or the Laughter Behavior for both time points of the experiment (during the manipulation phase, and during the free Interaction phase) as the predictor (treatment). Posterior means and 95% Equal-Tailed Intervals (ETIs) are reported.

| Treatment: Condition  Outcome: Liking | | | | | |
| --- | --- | --- | --- | --- | --- |
| ROI Pair | Direct Effect | Indirect Effect | Mediator Effect | Total Effect | Proportion Mediated |
| IFGl_IFGl | -0.23[-1.08, 0.66] | 0.00[-0.11, 0.11] | 0.69[-8.27, 9.74] | -0.23[-1.07, 0.66] | -0.13[-87.71, 87.45] |
| IFGl_TPJl | -0.34[-1.13, 0.45] | 0.01[-0.09, 0.21] | -6.69[-18.13, 4.75] | -0.31[-1.11, 0.48] | -5.50[-179.91, 168.91] |
| IFGl_TPJr | -0.22[-1.01, 0.53] | -0.02[-0.28, 0.19] | 11.09[2.33, 20.23] | -0.25[-1.07, 0.55] | 11.07[-257.12, 279.26] |
| IFGr_IFGl | -0.33[-1.13, 0.41] | 0.02[-0.07, 0.24] | 5.77[-4.34, 16.10] | -0.28[-1.09, 0.49] | -10.24[-238.56, 218.08] |
| IFGr_IFGr | -0.09[-0.98, 0.80] | -0.00[-0.22, 0.21] | -0.46[-10.22, 9.53] | -0.10[-0.98, 0.80] | 2.62[-238.84, 244.06] |
| IFGr_TPJl | -0.04[-0.90, 0.82] | 0.00[-0.17, 0.20] | 0.37[-10.98, 12.01] | -0.03[-0.89, 0.82] | -2.75[-226.56, 221.06] |
| IFGr_TPJr | -0.04[-0.92, 0.84] | -0.04[-0.32, 0.13] | -3.67[-15.05, 7.37] | -0.10[-0.95, 0.76] | 42.57[-290.55, 375.72] |
| TPJl_TPJl | -0.09[-1.09, 0.97] | -0.00[-0.24, 0.21] | -1.35[-14.56, 11.89] | -0.11[-1.11, 0.95] | 4.98[-173.69, 183.65] |
| TPJr_TPJl | 0.25[-0.64, 1.15] | -0.08[-0.42, 0.09] | 10.46[-1.61, 22.47] | 0.14[-0.78, 1.08] | -59.28[-492.63, 374.06] |
| TPJr_TPJr | 0.19[-0.68, 1.07] | 0.00[-0.13, 0.15] | -1.39[-10.49, 7.52] | 0.20[-0.67, 1.09] | 0.52[-124.79, 125.84] |
| Treatment: Laughter Behavior during Free Interaction phase  Outcome: Liking | | | | | |
| ROI Pair | Direct Effect | Indirect Effect | Mediator Effect | Total Effect | Proportion Mediated |
| IFGl_IFGl | 13676.11[5132.62, 22123.40] | -113.81[-2060.57, 1080.79] | -2.09[-10.35, 6.39] | 13445.44[4810.03, 22032.76] | -0.84[-15.41, 13.72] |
| IFGl_TPJl | 12093.69[4088.47, 20358.05] | 510.70[-613.40, 2934.92] | -7.28[-18.28, 3.71] | 12746.24[4779.02, 21100.22] | 4.00[-12.77, 20.78] |
| IFGl_TPJr | 11859.10[4041.88, 19485.87] | 363.80[-1316.50, 2848.59] | 9.06[-0.26, 18.23] | 12367.94[4394.43, 20326.00] | 2.93[-17.23, 23.12] |
| IFGr_IFGl | 12029.76[4018.08, 20333.56] | 44.64[-1134.16, 1591.02] | 4.33[-6.07, 15.22] | 12178.86[3985.61, 20462.03] | 0.36[-13.05, 13.78] |
| IFGr_IFGr | 11265.30[2445.04, 19911.33] | 0.06[-1308.15, 1326.32] | 0.14[-9.19, 9.54] | 11224.81[2549.11, 20066.90] | 0.00[-16.14, 16.14] |
| IFGr_TPJl | 12504.82[4036.47, 20724.53] | -47.39[-1825.81, 1325.85] | 1.78[-9.26, 12.97] | 12334.13[3997.97, 20446.51] | -0.38[-17.01, 16.25] |
| IFGr_TPJr | 13516.44[5184.75, 21561.07] | -245.42[-2541.59, 963.41] | -5.54[-16.46, 4.66] | 13044.11[4548.60, 21319.45] | -1.87[-20.17, 16.39] |
| TPJl_TPJl | 17043.38[7017.90, 26889.38] | -173.94[-2946.04, 1368.82] | -5.77[-17.80, 6.12] | 16660.26[6640.19, 26737.53] | -1.04[-17.85, 15.75] |
| TPJr_TPJl | 11705.09[2135.55, 20868.45] | 745.16[-1444.81, 4087.05] | 4.97[-6.92, 16.62] | 12676.77[3260.24, 21761.38] | 5.87[-22.89, 34.64] |
| TPJr_TPJr | 10767.11[1411.26, 19938.74] | -4.94[-1724.63, 1440.95] | -1.62[-10.02, 6.77] | 10778.80[1177.11, 19876.34] | -0.05[-23.17, 23.07] |
| Treatment: Condition  Outcome: Prosociality (Money Sharing) | | | | | |
| ROI Pair | Direct Effect | Indirect Effect | Mediator Effect | Total Effect | Proportion Mediated |
| IFGl_IFGl | -0.07[-0.24, 0.08] | -0.00[-0.02, 0.02] | -0.15[-1.96, 1.60] | -0.07[-0.24, 0.08] | 0.08[-85.03, 85.18] |
| IFGl_TPJl | -0.07[-0.24, 0.08] | 0.00[-0.01, 0.03] | -0.85[-3.26, 1.48] | -0.07[-0.23, 0.08] | -2.39[-116.45, 111.65] |
| IFGl_TPJr | -0.07[-0.23, 0.08] | -0.00[-0.02, 0.01] | 0.67[-1.07, 2.55] | -0.07[-0.23, 0.08] | 1.37[-95.29, 98.06] |
| IFGr_IFGl | -0.09[-0.26, 0.06] | -0.00[-0.03, 0.01] | -0.56[-2.59, 1.52] | -0.09[-0.27, 0.06] | 1.86[-87.42, 91.15] |
| IFGr_IFGr | -0.08[-0.26, 0.10] | -0.00[-0.06, 0.02] | -0.79[-2.72, 1.04] | -0.09[-0.28, 0.08] | 9.50[-156.53, 175.53] |
| IFGr_TPJl | -0.07[-0.23, 0.10] | -0.00[-0.05, 0.02] | -0.79[-3.16, 1.67] | -0.08[-0.24, 0.09] | 7.67[-145.34, 160.71] |
| IFGr_TPJr | -0.09[-0.28, 0.08] | 0.00[-0.04, 0.05] | 0.18[-2.16, 2.56] | -0.09[-0.26, 0.08] | -1.78[-169.02, 165.44] |
| TPJr_TPJl | -0.10[-0.29, 0.09] | -0.00[-0.05, 0.02] | 0.63[-1.88, 3.27] | -0.10[-0.30, 0.08] | 3.14[-116.51, 122.78] |
| IFGl_IFGl | -0.07[-0.24, 0.08] | -0.00[-0.02, 0.02] | -0.15[-1.96, 1.60] | -0.07[-0.24, 0.08] | 0.08[-85.03, 85.18] |
| IFGl_TPJl | -0.07[-0.24, 0.08] | 0.00[-0.01, 0.03] | -0.85[-3.26, 1.48] | -0.07[-0.23, 0.08] | -2.39[-116.45, 111.65] |
| Treatment: Laughter Behavior during Free Interaction phase  Outcome: Prosociality (Money Sharing) | | | | | |
| ROI Pair | Direct Effect | Indirect Effect | Mediator Effect | Total Effect | Proportion Mediated |
| IFGl_IFGl | 1241.72[-480.62, 2924.93] | -3.73[-336.33, 273.37] | -0.10[-1.81, 1.54] | 1214.39[-510.88, 2902.05] | -0.30[-69.71, 69.09] |
| IFGl_TPJl | 1082.54[-501.24, 2725.73] | 73.94[-148.56, 507.24] | -1.13[-3.49, 1.11] | 1188.69[-358.08, 2850.57] | 6.21[-83.51, 95.95] |
| IFGl_TPJr | 1170.21[-424.65, 2771.05] | 8.04[-174.47, 301.62] | 0.47[-1.24, 2.28] | 1200.03[-406.22, 2803.42] | 0.67[-48.00, 49.35] |
| IFGr_IFGl | 1279.47[-392.86, 2883.48] | -0.28[-218.95, 209.44] | -0.16[-2.22, 1.95] | 1266.13[-390.77, 2879.15] | -0.02[-43.17, 43.13] |
| IFGr_IFGr | 1231.22[-557.87, 2974.08] | 2.64[-253.07, 312.37] | -0.55[-2.42, 1.34] | 1242.69[-546.42, 3045.50] | 0.20[-55.15, 55.57] |
| IFGr_TPJl | 1277.92[-370.17, 2981.79] | 32.45[-196.14, 439.96] | -0.73[-2.89, 1.49] | 1342.33[-322.00, 3020.23] | 2.41[-51.00, 55.84] |
| IFGr_TPJr | 1466.32[-249.70, 3149.75] | -8.78[-345.93, 219.53] | -0.33[-2.49, 1.73] | 1436.61[-279.14, 3167.38] | -0.60[-50.85, 49.63] |
| TPJl_TPJl | 1215.50[-797.49, 3159.18] | -30.65[-560.09, 286.74] | -1.14[-3.64, 1.29] | 1147.40[-900.64, 3088.19] | -2.66[-130.46, 125.12] |
| TPJr_TPJl | 1017.23[-1026.22, 3099.35] | 71.35[-423.30, 719.18] | 0.50[-1.91, 3.02] | 1114.14[-843.16, 3106.17] | 6.40[-148.15, 160.96] |
| TPJr_TPJr | 1580.43[-695.13, 3856.63] | -24.39[-706.99, 557.49] | -1.62[-3.59, 0.41] | 1537.78[-778.13, 3858.82] | -1.59[-128.81, 125.65] |
| Treatment: Condition  Outcome: Prosociality (Helping) | | | | | |
| ROI Pair | Direct Effect | Indirect Effect | Mediator Effect | Total Effect | Proportion Mediated |
| IFGl_IFGl | -0.01[-0.14, 0.12] | -0.00[-0.02, 0.01] | -0.72[-2.06, 0.68] | -0.01[-0.14, 0.12] | 4.28[-160.28, 168.84] |
| IFGl_TPJl | -0.01[-0.14, 0.11] | 0.00[-0.01, 0.03] | -1.21[-3.16, 0.68] | -0.01[-0.14, 0.12] | -31.71[-231.00, 167.56] |
| IFGl_TPJr | -0.01[-0.15, 0.11] | -0.00[-0.02, 0.01] | 0.38[-1.23, 1.97] | -0.01[-0.15, 0.11] | 1.82[-129.78, 133.41] |
| IFGr_IFGl | -0.00[-0.14, 0.13] | -0.00[-0.03, 0.01] | -0.83[-2.53, 0.92] | -0.01[-0.14, 0.12] | 34.29[-183.06, 251.65] |
| IFGr_IFGr | -0.02[-0.17, 0.13] | 0.00[-0.03, 0.03] | 0.05[-1.56, 1.70] | -0.01[-0.17, 0.13] | -1.60[-215.77, 212.58] |
| IFGr_TPJl | -0.00[-0.14, 0.13] | -0.00[-0.03, 0.02] | -0.53[-2.37, 1.28] | -0.01[-0.15, 0.12] | 32.53[-221.66, 286.74] |
| IFGr_TPJr | -0.02[-0.16, 0.11] | 0.01[-0.01, 0.05] | 0.82[-0.95, 2.62] | -0.01[-0.14, 0.12] | -67.51[-398.43, 263.41] |
| TPJl_TPJl | -0.01[-0.17, 0.14] | 0.01[-0.01, 0.06] | 1.38[-0.55, 3.33] | 0.00[-0.16, 0.17] | 620.59[312.37, 928.80] |
| TPJr_TPJl | 0.01[-0.13, 0.16] | -0.00[-0.04, 0.02] | 0.71[-1.39, 2.80] | 0.01[-0.14, 0.16] | -34.10[-272.22, 204.00] |
| TPJr_TPJr | -0.00[-0.17, 0.17] | 0.00[-0.02, 0.03] | -0.63[-2.24, 1.03] | -0.00[-0.17, 0.17] | -15.86[-182.62, 150.87] |
| Treatment: Laughter Behavior during Free Interaction phase  Outcome: Prosociality (Helping) | | | | | |
| ROI Pair | Direct Effect | Indirect Effect | Mediator Effect | Total Effect | Proportion Mediated |
| IFGl_IFGl | 852.19[-666.18, 2388.55] | -69.67[-517.66, 146.36] | -0.90[-2.36, 0.56] | 745.68[-821.06, 2274.62] | -9.33[-191.72, 173.03] |
| IFGl_TPJl | 633.10[-711.00, 2060.19] | 108.47[-112.61, 552.98] | -1.43[-3.36, 0.46] | 774.55[-584.41, 2187.46] | 14.00[-159.37, 187.37] |
| IFGl_TPJr | 829.30[-540.75, 2206.94] | 3.12[-159.17, 237.18] | 0.24[-1.28, 1.82] | 844.90[-513.67, 2242.47] | 0.36[-57.74, 58.47] |
| IFGr_IFGl | 997.60[-434.42, 2400.02] | -13.33[-313.81, 196.23] | -0.91[-2.67, 0.91] | 974.03[-503.78, 2400.65] | -1.37[-75.40, 72.67] |
| IFGr_IFGr | 913.72[-640.35, 2468.59] | 0.68[-219.89, 205.23] | 0.11[-1.52, 1.71] | 919.43[-645.32, 2463.93] | 0.07[-58.46, 58.60] |
| IFGr_TPJl | 1100.95[-316.70, 2520.76] | 12.65[-187.16, 330.97] | -0.36[-2.21, 1.47] | 1145.61[-290.70, 2560.23] | 1.10[-50.47, 52.68] |
| IFGr_TPJr | 1118.98[-255.93, 2500.71] | 25.05[-147.79, 344.05] | 0.64[-1.10, 2.36] | 1168.51[-219.27, 2609.64] | 2.14[-50.78, 55.07] |
| TPJl_TPJl | 1124.59[-554.06, 2866.59] | 20.46[-254.65, 466.08] | 0.95[-1.16, 3.09] | 1182.69[-556.39, 2930.46] | 1.72[-79.29, 82.76] |
| TPJr_TPJl | 966.29[-657.94, 2642.93] | 45.37[-374.08, 592.86] | 0.34[-1.71, 2.46] | 1028.68[-605.12, 2660.84] | 4.41[-129.38, 138.21] |
| TPJr_TPJr | 1655.49[-163.78, 3525.68] | -4.30[-373.37, 293.58] | -0.58[-2.25, 1.04] | 1638.12[-244.58, 3531.03] | -0.26[-39.52, 39.00] |
| Treatment: Condition  Outcome: Bonding | | | | | |
| ROI Pair | Direct Effect | Indirect Effect | Mediator Effect | Total Effect | Proportion Mediated |
| IFGl_IFGl | -0.19[-0.89, 0.46] | 0.00[-0.07, 0.09] | 1.36[-5.37, 8.43] | -0.18[-0.89, 0.47] | -0.44[-96.17, 95.29] |
| IFGl_TPJl | -0.29[-0.94, 0.35] | 0.00[-0.07, 0.13] | -3.54[-13.25, 6.06] | -0.27[-0.93, 0.37] | -2.35[-121.62, 116.90] |
| IFGl_TPJr | -0.26[-0.87, 0.35] | -0.00[-0.10, 0.07] | 1.46[-6.23, 9.13] | -0.26[-0.88, 0.36] | 0.47[-78.84, 79.79] |
| IFGr_IFGl | -0.36[-1.02, 0.30] | 0.01[-0.07, 0.17] | 3.49[-4.96, 12.43] | -0.34[-0.99, 0.33] | -4.17[-131.06, 122.70] |
| IFGr_IFGr | -0.27[-1.04, 0.50] | 0.07[-0.06, 0.36] | 6.03[-2.08, 14.24] | -0.17[-0.94, 0.61] | -43.79[-438.62, 351.04] |
| IFGr_TPJl | -0.16[-0.84, 0.48] | -0.00[-0.15, 0.15] | 0.07[-9.14, 9.27] | -0.17[-0.85, 0.49] | 0.04[-185.78, 185.87] |
| IFGr_TPJr | -0.21[-0.88, 0.48] | 0.08[-0.05, 0.33] | 6.27[-2.49, 15.18] | -0.11[-0.80, 0.59] | -72.12[-530.38, 386.16] |
| TPJl_TPJl | -0.16[-1.00, 0.69] | 0.03[-0.09, 0.29] | 4.98[-4.85, 15.30] | -0.11[-0.93, 0.75] | -31.91[-307.79, 243.97] |
| TPJr_TPJl | -0.02[-0.80, 0.74] | -0.09[-0.40, 0.07] | 10.71[0.56, 20.75] | -0.14[-0.92, 0.66] | 65.03[-372.75, 502.81] |
| TPJr_TPJr | -0.05[-0.88, 0.77] | 0.00[-0.12, 0.19] | -3.17[-11.65, 5.15] | -0.04[-0.89, 0.81] | -13.39[-191.08, 164.31] |
| Treatment: Laughter Behavior during Free Interaction phase  Outcome: Bonding | | | | | |
| ROI Pair | Direct Effect | Indirect Effect | Mediator Effect | Total Effect | Proportion Mediated |
| IFGl_IFGl | 8070.25[1477.49, 14942.12] | -11.00[-1232.12, 1116.02] | -0.24[-7.10, 6.59] | 8063.69[1451.10, 14955.34] | -0.14[-21.92, 21.64] |
| IFGl_TPJl | 6733.55[177.92, 13529.41] | 204.85[-813.67, 2057.96] | -3.38[-13.36, 6.51] | 7135.04[497.62, 13841.57] | 2.87[-30.03, 35.78] |
| IFGl_TPJr | 7251.43[568.11, 13714.62] | -2.21[-932.65, 933.48] | 0.03[-7.29, 7.71] | 7198.43[684.28, 13822.74] | -0.02[-21.07, 21.01] |
| IFGr_IFGl | 6665.35[33.82, 13494.67] | 21.68[-844.25, 1285.02] | 2.64[-6.11, 11.68] | 6746.77[71.24, 13578.20] | 0.32[-25.10, 25.76] |
| IFGr_IFGr | 6282.32[-1790.70, 14375.67] | -51.98[-2187.59, 1749.31] | 6.34[-1.89, 14.62] | 6145.76[-2061.08, 14370.45] | -0.84[-90.59, 88.90] |
| IFGr_TPJl | 6607.93[-473.42, 13481.74] | -8.73[-1319.21, 1208.20] | 0.44[-9.23, 9.83] | 6609.06[-513.42, 13573.95] | -0.13[-39.34, 39.07] |
| IFGr_TPJr | 5917.61[-1103.39, 12521.31] | 238.86[-769.88, 2161.72] | 4.99[-3.62, 13.68] | 6285.52[-753.88, 13079.87] | 3.79[-45.71, 53.32] |
| TPJl_TPJl | 6998.22[-1232.63, 15548.74] | 79.16[-1131.65, 2001.07] | 3.47[-6.36, 13.65] | 7215.76[-986.80, 15685.95] | 1.10[-48.99, 51.18] |
| TPJr_TPJl | 7641.03[-574.86, 16177.47] | 1143.63[-650.68, 4681.65] | 7.14[-3.30, 17.12] | 9111.80[881.95, 17281.25] | 12.55[-42.07, 67.18] |
| TPJr_TPJr | 9419.49[524.72, 18085.24] | -29.95[-2170.47, 1606.61] | -3.95[-12.28, 3.71] | 9285.13[287.52, 18128.71] | -0.32[-38.25, 37.60] |

Table 10. Posterior estimates from Bayesian mediation models for Synchrony during the free Interaction phase. Social outcome variables (Liking, Prosociality (Money Sharing, and Helping), and Bonding) by ROI Pair. Separate models were computed for each ROI pair (as the mediator) with either the Laughter Condition (Laughter experience vs. no Laughter experience) or the Laughter Behavior during the free interaction phase as the predictor (treatment). Posterior means and 95% Equal-Tailed Intervals (ETIs) are reported.

## HbR Data analyses

To provide a comprehensive picture of the fNIRS data and to assess potential differences from the oxygenated hemoglobin (HbO) results, we additionally analyzed the deoxygenated hemoglobin (HbR) signal. Specifically, we conducted the same analyses for Hypothesis 1 and Hypothesis 3 using the HbR data. For the comparison of synchrony between real and surrogate data as well as for Hypothesis 1, the analyses revealed no meaningful effects. Additionally, no evidence for a mediation effect was found. However, several interesting direct effects emerged from the mediation analyses. Specifically, we observed direct effects of LBFI on Liking, Money Sharing, and Bonding. Regarding synchrony during the manipulation phase, we found direct effects of synchrony between TPJr and TPJl on Money Sharing and between IFGr and TPJl on Helping. During the free interaction phase, synchrony between IFGl_IFGl was positively associated with Liking. Furthermore, we observed negative associations between synchrony and outcomes in several cases. For the manipulation phase, synchrony between IFGl_IFGl was negatively associated with Liking. During the free interaction phase, negative associations were observed between TPJr_TPJr and Money Sharing, IFGr_TPJr and Helping, as well as IFGl_TPJr and IFGr_TPJr with Bonding.

For a detailed overview of all results, please refer to the following tables.

| **ROI pair** | **Real Estimate [95% CI]** | **Interval Estimate [95% CI]** | **Real*Interval Estimate [95% CI]** |
| --- | --- | --- | --- |
| **IFGl_IFGl** | -0.01 [-0.03, 0.01] | -0.00 [-0.02, 0.02] | 0.01 [-0.02, 0.04] |
| **IFGl_TPJl** | 0.00 [-0.01, 0.02] | 0.00 [-0.01, 0.02] | 0.00 [-0.02, 0.03] |
| **IFGl_TPJr** | 0.00 [-0.01, 0.02] | 0.00 [-0.02, 0.02] | -0.00 [-0.03, 0.02] |
| **IFGr_IFGl** | 0.00 [-0.01, 0.02] | -0.00 [-0.02, 0.01] | -0.00 [-0.03, 0.02] |
| **IFGr_IFGr** | 0.00 [-0.02, 0.03] | -0.00 [-0.02, 0.02] | -0.00 [-0.04, 0.03] |
| **IFGr_TPJl** | 0.00 [-0.01, 0.02] | 0.00 [-0.01, 0.02] | -0.00 [-0.03, 0.02] |
| **IFGr_TPJr** | 0.00 [-0.01, 0.02] | 0.00 [-0.01, 0.02] | -0.00 [-0.03, 0.02] |
| **TPJl_TPJl** | 0.01 [-0.01, 0.04] | -0.00 [-0.02, 0.02] | -0.01 [-0.05, 0.02] |
| **TPJr_TPJl** | 0.00 [-0.01, 0.03] | 0.00 [-0.01, 0.02] | -0.00 [-0.03, 0.02] |
| **TPJr_TPJr** | 0.00 [-0.02, 0.03] | 0.00 [-0.02, 0.02] | -0.01 [-0.05, 0.02] |

Table 11. This table reports the Bayesian parameter estimates for synchrony during the manipulation phase using HbR data, comparing real data and surrogate data (random permutation). Predictors include Real (real data vs. surrogate data), Interval (first 5-minute video vs. second 5-minute video), and the interaction term Real * Interval. The analysis was conducted for 10 ROI pairs, consisting of the inferior frontal gyrus (IFG) and the temporoparietal junction (TPJ), labeled as "l" (left hemisphere) and "r" (right hemisphere).

| **ROI pair** | **Real Estimate [95% CI]** | **Interval Estimate [95% CI]** | **Real*Interval Estimate [95% CI]** |
| --- | --- | --- | --- |
| **IFGl_IFGl** | -0.00 [-0.09, 0.08] | 0.00 [-0.06, 0.07] | 0.00 [-0.06, 0.07] |
| **IFGl_TPJl** | 0.00 [-0.01, 0.02] | -0.00 [-0.02, 0.02] | -0.00 [-0.03, 0.02] |
| **IFGl_TPJr** | 0.00 [-0.01, 0.02] | -0.00 [-0.02, 0.01] | -0.00 [-0.03, 0.02] |
| **IFGr_IFGl** | 0.00 [-0.01, 0.02] | -0.00 [-0.02, 0.01] | -0.00 [-0.02, 0.02] |
| **IFGr_IFGr** | -0.00 [-0.08, 0.08] | -0.01 [-0.08, 0.06] | -0.01 [-0.08, 0.06] |
| **IFGr_TPJl** | 0.00 [-0.01, 0.03] | 0.00 [-0.01, 0.02] | -0.00 [-0.03, 0.02] |
| **IFGr_TPJr** | 0.00 [-0.01, 0.03] | -0.00 [-0.02, 0.01] | -0.01 [-0.04, 0.02] |
| **TPJl_TPJl** | -0.00 [-0.09, 0.09] | 0.01 [-0.05, 0.08] | 0.01 [-0.05, 0.08] |
| **TPJr_TPJl** | 0.01 [-0.01, 0.03] | 0.00 [-0.01, 0.02] | -0.00 [-0.03, 0.02] |
| **TPJr_TPJr** | -0.00 [-0.09, 0.09] | -0.00 [-0.07, 0.06] | -0.00 [-0.07, 0.06] |

Table 12. This table reports the Bayesian parameter estimates for synchrony during the free interaction phase using HbR data, comparing real data and surrogate data (random permutation). Predictors include Real (real data vs. surrogate data), Interval (first 5-minute segment vs. second 5-minute segment), and the interaction term Real * Interval. The analysis was conducted for 10 ROI pairs, consisting of the inferior frontal gyrus (IFG) and the temporoparietal junction (TPJ), labeled as "l" (left hemisphere) and "r" (right hemisphere).

| **Parameter Estimate [95% CI]** | **IFGl_ IFGl** | **IFGl_ TPJl** | **IFGl_ TPJr** | **IFGr_ IFGl** | **IFGr_ IFGr** | **IFGr_ TPJl** | **IFGr_ TPJr** | **TPJl_ TPJl** | **TPJr_ TPJl** | **TPJr_ TPJr** |
| --- | --- | --- | --- | --- | --- | --- | --- | --- | --- | --- |
| **Sample size** | N (Dyad) = 95  N( Interval 1) = 95  N (Interval 2) = 94 | N (Dyad) = 94  N( Interval 1) = 160  N (Interval 2) = 155 | N (Dyad) = 94  N( Interval 1) = 160  N (Interval 2) = 156 | N (Dyad) = 96  N( Interval 1) = 181  N (Interval 2) = 177 | N (Dyad) = 87  N( Interval 1) = 86  N (Interval 2) = 83 | N (Dyad) = 88  N( Interval 1) = 152  N (Interval 2) = 145 | N (Dyad) = 89  N( Interval 1) = 132  N (Interval 2) = 126 | N (Dyad) = 68  N( Interval 1) = 66  N (Interval 2) = 63 | N (Dyad) = 78  N( Interval 1) = 132  N (Interval 2) = 126 | N (Dyad) = 68  N( Interval 1) = 66  N (Interval 2) = 63 |
| **Intercept** | 0.26 [0.23, 0.29] | 0.27 [0.25, 0.30] | 0.28 [0.25, 0.30] | 0.27 [0.24, 0.29] | 0.27 [0.24, 0.31] | 0.27 [0.25, 0.30] | 0.28 [0.25, 0.31] | 0.29 [0.25, 0.33] | 0.28 [0.25, 0.31] | 0.27 [0.23, 0.31] |
| **Laughter Manipulation** | -0.02 [-0.06, 0.02] | -0.00 [-0.04, 0.03] | -0.00 [-0.04, 0.02] | -0.00 [-0.03, 0.03] | -0.00 [-0.05, 0.03] | -0.00 [-0.03, 0.03] | -0.00 [-0.04, 0.03] | -0.00 [-0.05, 0.04] | 0.00 [-0.03, 0.04] | -0.00 [-0.04, 0.04] |
| **Social** | 0.01 [-0.02, 0.05] | 0.00 [-0.03, 0.03] | 0.00 [-0.03, 0.03] | 0.02 [-0.00, 0.05] | 0.02 [-0.02, 0.06] | 0.00 [-0.03, 0.03] | -0.00 [-0.04, 0.02] | -0.02 [-0.07, 0.02] | -0.00 [-0.04, 0.03] | 0.00 [-0.04, 0.04] |
| **LBMP** | -0.00 [-0.09, 0.09] | 0.00 [-0.09, 0.09] | 0.00 [-0.09, 0.09] | 0.00 [-0.09, 0.09] | 0.00 [-0.09, 0.09] | 0.00 [-0.09, 0.09] | 0.00 [-0.09, 0.09] | 0.00 [-0.09, 0.09] | 0.00 [-0.09, 0.09] | -0.00 [-0.09, 0.09] |
| **Interval** | 0.01 [-0.02, 0.05] | 0.00 [-0.03, 0.03] | -0.02 [-0.05, 0.01] | -0.00 [-0.03, 0.02] | 0.00 [-0.04, 0.04] | -0.00 [-0.03, 0.03] | -0.01 [-0.05, 0.02] | -0.01 [-0.06, 0.03] | -0.01 [-0.05, 0.02] | -0.01 [-0.06, 0.03] |
| **Laughter Manipulation:Social** | 0.00 [-0.04, 0.06] | 0.00 [-0.04, 0.05] | -0.00 [-0.05, 0.04] | -0.01 [-0.05, 0.03] | 0.00 [-0.04, 0.06] | 0.00 [-0.04, 0.05] | 0.00 [-0.04, 0.05] | 0.02 [-0.03, 0.08] | -0.00 [-0.05, 0.04] | -0.00 [-0.06, 0.05] |
| **Laughter Manipulation:Interval** | 0.00 [-0.05, 0.05] | 0.00 [-0.03, 0.05] | 0.01 [-0.03, 0.06] | 0.00 [-0.04, 0.04] | -0.00 [-0.05, 0.05] | -0.00 [-0.05, 0.04] | -0.00 [-0.05, 0.04] | -0.00 [-0.06, 0.05] | -0.00 [-0.05, 0.04] | -0.00 [-0.05, 0.05] |
| **Social:Interval** | -0.01 [-0.06, 0.03] | 0.00 [-0.04, 0.05] | 0.01 [-0.02, 0.06] | -0.01 [-0.06, 0.02] | -0.01 [-0.07, 0.03] | 0.00 [-0.04, 0.05] | 0.01 [-0.03, 0.06] | -0.00 [-0.06, 0.05] | 0.01 [-0.03, 0.06] | 0.01 [-0.04, 0.07] |
| **Laughter Manipulation:LBMP** | -0.00 [-0.09, 0.09] | 0.00 [-0.09, 0.09] | 0.00 [-0.09, 0.09] | -0.00 [-0.09, 0.09] | -0.00 [-0.10, 0.09] | -0.00 [-0.10, 0.10] | -0.00 [-0.09, 0.09] | 0.00 [-0.09, 0.09] | 0.00 [-0.09, 0.09] | 0.00 [-0.09, 0.09] |
| **Social:LBMP** | -0.00 [-0.09, 0.09] | -0.00 [-0.09, 0.09] | -0.00 [-0.09, 0.09] | -0.00 [-0.09, 0.09] | 0.00 [-0.09, 0.09] | -0.00 [-0.09, 0.09] | -0.00 [-0.10, 0.09] | -0.00 [-0.09, 0.09] | 0.00 [-0.09, 0.09] | 0.00 [-0.09, 0.09] |
| **Interval:LBMP** | -0.00 [-0.09, 0.09] | 0.00 [-0.09, 0.09] | -0.00 [-0.09, 0.09] | 0.00 [-0.09, 0.09] | -0.00 [-0.09, 0.09] | 0.00 [-0.09, 0.09] | -0.00 [-0.09, 0.09] | 0.00 [-0.09, 0.09] | 0.00 [-0.09, 0.09] | -0.00 [-0.09, 0.09] |
| **Laughter Manipulation:Social:Interval** | 0.01 [-0.05, 0.07] | -0.00 [-0.07, 0.05] | 0.01 [-0.04, 0.07] | 0.01 [-0.04, 0.07] | -0.01 [-0.08, 0.05] | 0.01 [-0.04, 0.08] | 0.02 [-0.03, 0.08] | -0.00 [-0.08, 0.06] | 0.01 [-0.04, 0.08] | 0.00 [-0.07, 0.07] |
| **Laughter Manipulation:Social:LBMP** | -0.00 [-0.09, 0.09] | 0.00 [-0.09, 0.09] | -0.00 [-0.09, 0.09] | 0.00 [-0.09, 0.09] | 0.00 [-0.09, 0.09] | -0.00 [-0.09, 0.09] | 0.00 [-0.09, 0.09] | -0.00 [-0.09, 0.09] | 0.00 [-0.09, 0.10] | -0.00 [-0.09, 0.09] |
| **Laughter Manipulation:Interval:LBMP** | 0.00 [-0.09, 0.09] | -0.00 [-0.09, 0.09] | 0.00 [-0.09, 0.09] | 0.00 [-0.09, 0.10] | -0.00 [-0.09, 0.09] | -0.00 [-0.09, 0.09] | 0.00 [-0.10, 0.10] | -0.00 [-0.09, 0.09] | -0.00 [-0.09, 0.09] | -0.00 [-0.09, 0.09] |
| **Social:Interval:LBMP** | 0.00 [-0.09, 0.09] | -0.00 [-0.09, 0.09] | -0.00 [-0.09, 0.09] | 0.00 [-0.09, 0.09] | -0.00 [-0.09, 0.09] | -0.00 [-0.09, 0.09] | -0.00 [-0.09, 0.09] | 0.00 [-0.09, 0.09] | -0.00 [-0.09, 0.09] | 0.00 [-0.09, 0.09] |
| **Laughter Manipulation:Social:Interval:LBMP** | 0.00 [-0.10, 0.10] | 0.00 [-0.09, 0.09] | 0.00 [-0.09, 0.10] | -0.00 [-0.09, 0.09] | -0.00 [-0.09, 0.09] | -0.00 [-0.09, 0.09] | 0.00 [-0.09, 0.09] | -0.00 [-0.10, 0.09] | -0.00 [-0.09, 0.09] | 0.00 [-0.09, 0.09] |

Table 13. Posterior estimates for all effects from the Bayesian parameter estimations by ROI pair for synchrony measured during the manipulation phase using HbR data. The analysis was conducted for 10 ROI pairs, consisting of the inferior frontal gyrus (IFG) and the temporoparietal junction (TPJ), labeled as "l" (left hemisphere) and "r" (right hemisphere). The model includes the predictors laughter manipulation (yes/no), social context (yes/no), interval (first or second 5-minute video), and LBMP (laughter behavior during the manipulation phase). Sample sizes for Interval 1 and Interval 2 refer to the number of WTC values, with each dyad contributing one measure per interval (two per dyad overall). For heterogeneous ROI pairs, sample sizes are higher than for homologous pairs because bidirectional synchrony (e.g., TPJl-IFGr and IFGr-TPJl) is included together in one ROI-pair analysis.

| **Parameter Estimate [95% CI]** | **IFGl_ IFGl** | **IFGl_ TPJl** | **IFGl_ TPJr** | **IFGr_ IFGl** | **IFGr_ IFGr** | **IFGr_ TPJl** | **IFGr_ TPJr** | **TPJl_ TPJl** | **TPJr_ TPJl** | **TPJr_ TPJr** |
| --- | --- | --- | --- | --- | --- | --- | --- | --- | --- | --- |
| **Sample size** | N (Dyad) = 86  N( Interval 1) = 84  N (Interval 2) = 79 | N (Dyad) = 90  N( Interval 1) = 141  N (Interval 2) = 135 | N (Dyad) = 91  N( Interval 1) = 139  N (Interval 2) = 132 | N (Dyad) = 88  N( Interval 1) = 146  N (Interval 2) = 141 | N (Dyad) = 70  N( Interval 1) = 65  N (Interval 2) = 64 | N (Dyad) = 81  N( Interval 1) = 124  N (Interval 2) = 122 | N (Dyad) = 82  N( Interval 1) = 122  N (Interval 2) = 122 | N (Dyad) = 62  N( Interval 1) = 58  N (Interval 2) = 57 | N (Dyad) = 70  N( Interval 1) = 112  N (Interval 2) = 109 | N (Dyad) = 60  N( Interval 1) = 56  N (Interval 2) = 54 |
| **Intercept** | 0.29 [0.26, 0.33] | 0.28 [0.25, 0.30] | 0.29 [0.26, 0.32] | 0.28 [0.26, 0.31] | 0.27 [0.23, 0.31] | 0.27 [0.24, 0.30] | 0.29 [0.26, 0.32] | 0.26 [0.22, 0.30] | 0.29 [0.26, 0.32] | 0.30 [0.25, 0.34] |
| **Laughter Manipulation** | -0.00 [-0.04, 0.04] | -0.00 [-0.04, 0.03] | -0.02 [-0.06, 0.01] | 0.02 [-0.01, 0.06] | 0.00 [-0.04, 0.05] | 0.01 [-0.03, 0.05] | -0.00 [-0.04, 0.03] | 0.01 [-0.03, 0.06] | -0.02 [-0.06, 0.01] | -0.01 [-0.07, 0.03] |
| **Social** | 0.00 [-0.03, 0.05] | 0.00 [-0.02, 0.04] | -0.00 [-0.04, 0.02] | -0.00 [-0.04, 0.02] | 0.00 [-0.03, 0.05] | 0.00 [-0.03, 0.04] | -0.00 [-0.04, 0.02] | -0.00 [-0.05, 0.04] | 0.00 [-0.03, 0.04] | 0.00 [-0.04, 0.05] |
| **LBMP** | 0.00 [-0.09, 0.09] | -0.00 [-0.09, 0.09] | -0.00 [-0.09, 0.10] | 0.00 [-0.09, 0.09] | 0.00 [-0.09, 0.09] | 0.00 [-0.09, 0.09] | 0.00 [-0.09, 0.09] | 0.00 [-0.09, 0.09] | 0.00 [-0.09, 0.10] | 0.00 [-0.09, 0.09] |
| **LBFI** | 0.00 [-0.09, 0.09] | -0.00 [-0.09, 0.09] | -0.00 [-0.09, 0.09] | 0.00 [-0.09, 0.09] | -0.00 [-0.09, 0.09] | -0.00 [-0.09, 0.09] | 0.00 [-0.09, 0.09] | 0.00 [-0.09, 0.09] | -0.00 [-0.09, 0.09] | 0.00 [-0.10, 0.09] |
| **Interval** | -0.00 [-0.05, 0.03] | 0.00 [-0.03, 0.03] | -0.00 [-0.04, 0.02] | 0.00 [-0.03, 0.03] | -0.00 [-0.05, 0.03] | 0.01 [-0.02, 0.05] | -0.01 [-0.05, 0.02] | 0.02 [-0.02, 0.07] | -0.00 [-0.04, 0.03] | -0.00 [-0.05, 0.04] |
| **Laughter Manipulation:Social** | -0.03 [-0.09, 0.02] | -0.00 [-0.05, 0.04] | -0.00 [-0.05, 0.04] | -0.02 [-0.07, 0.02] | 0.01 [-0.04, 0.08] | 0.01 [-0.03, 0.06] | -0.00 [-0.05, 0.04] | 0.00 [-0.06, 0.06] | 0.01 [-0.04, 0.06] | -0.00 [-0.06, 0.06] |
| **Laughter Manipulation:Interval** | 0.01 [-0.04, 0.07] | 0.01 [-0.03, 0.06] | 0.02 [-0.02, 0.07] | -0.02 [-0.07, 0.02] | -0.01 [-0.07, 0.05] | -0.01 [-0.06, 0.03] | 0.01 [-0.03, 0.06] | -0.00 [-0.06, 0.05] | 0.00 [-0.04, 0.05] | 0.00 [-0.05, 0.06] |
| **Social:Interval** | 0.00 [-0.04, 0.06] | -0.01 [-0.06, 0.03] | 0.00 [-0.04, 0.04] | 0.00 [-0.04, 0.04] | -0.01 [-0.07, 0.04] | -0.00 [-0.05, 0.04] | 0.00 [-0.04, 0.05] | 0.00 [-0.06, 0.06] | -0.01 [-0.06, 0.04] | -0.01 [-0.07, 0.04] |
| **Laughter Manipulation:LBMP** | 0.00 [-0.09, 0.09] | 0.00 [-0.09, 0.09] | -0.00 [-0.09, 0.09] | -0.00 [-0.10, 0.09] | -0.00 [-0.09, 0.09] | -0.00 [-0.10, 0.09] | 0.00 [-0.09, 0.09] | 0.00 [-0.09, 0.09] | 0.00 [-0.09, 0.09] | 0.00 [-0.09, 0.09] |
| **Social:LBMP** | -0.00 [-0.09, 0.09] | 0.00 [-0.09, 0.09] | 0.00 [-0.09, 0.09] | -0.00 [-0.09, 0.09] | -0.00 [-0.09, 0.09] | 0.00 [-0.09, 0.09] | 0.00 [-0.09, 0.09] | -0.00 [-0.09, 0.09] | -0.00 [-0.09, 0.09] | -0.00 [-0.09, 0.09] |
| **Interval:LBMP** | 0.00 [-0.09, 0.09] | 0.00 [-0.09, 0.09] | -0.00 [-0.09, 0.09] | -0.00 [-0.09, 0.09] | 0.00 [-0.09, 0.09] | 0.00 [-0.09, 0.09] | 0.00 [-0.09, 0.09] | -0.00 [-0.09, 0.09] | -0.00 [-0.09, 0.09] | -0.00 [-0.09, 0.09] |
| **Laughter Manipulation:LBFI** | 0.00 [-0.09, 0.09] | -0.00 [-0.10, 0.09] | 0.00 [-0.09, 0.09] | -0.00 [-0.09, 0.09] | 0.00 [-0.09, 0.09] | -0.00 [-0.09, 0.09] | -0.00 [-0.09, 0.09] | 0.00 [-0.09, 0.09] | 0.00 [-0.09, 0.09] | 0.00 [-0.09, 0.09] |
| **Social:LBFI** | 0.00 [-0.09, 0.10] | -0.00 [-0.09, 0.09] | -0.00 [-0.09, 0.09] | -0.00 [-0.09, 0.09] | -0.00 [-0.09, 0.09] | 0.00 [-0.09, 0.09] | -0.00 [-0.09, 0.09] | -0.00 [-0.09, 0.09] | 0.00 [-0.09, 0.09] | 0.00 [-0.09, 0.09] |
| **Interval:LBFI** | 0.00 [-0.09, 0.09] | -0.00 [-0.09, 0.09] | 0.00 [-0.09, 0.09] | -0.00 [-0.09, 0.09] | -0.00 [-0.09, 0.09] | -0.00 [-0.09, 0.09] | -0.00 [-0.09, 0.10] | 0.00 [-0.09, 0.09] | -0.00 [-0.09, 0.09] | 0.00 [-0.09, 0.09] |
| **LBMP:LBFI** | -0.00 [-0.09, 0.09] | 0.00 [-0.09, 0.09] | -0.00 [-0.09, 0.09] | 0.00 [-0.09, 0.09] | 0.00 [-0.09, 0.09] | -0.00 [-0.09, 0.09] | -0.00 [-0.10, 0.09] | 0.00 [-0.09, 0.10] | 0.00 [-0.09, 0.09] | 0.00 [-0.09, 0.09] |
| **Laughter Manipulation:Social:Interval** | 0.00 [-0.06, 0.07] | -0.00 [-0.07, 0.05] | -0.00 [-0.06, 0.05] | 0.03 [-0.03, 0.09] | 0.01 [-0.06, 0.08] | -0.00 [-0.06, 0.06] | -0.01 [-0.07, 0.04] | 0.00 [-0.07, 0.08] | 0.03 [-0.03, 0.09] | 0.00 [-0.07, 0.07] |
| **Laughter Manipulation:Social:LBMP** | 0.00 [-0.09, 0.09] | -0.00 [-0.09, 0.09] | 0.00 [-0.09, 0.09] | 0.00 [-0.09, 0.09] | -0.00 [-0.10, 0.09] | -0.00 [-0.09, 0.09] | 0.00 [-0.09, 0.10] | 0.00 [-0.09, 0.09] | 0.00 [-0.09, 0.09] | -0.00 [-0.09, 0.09] |
| **Laughter Manipulation:Interval:LBMP** | -0.00 [-0.10, 0.09] | 0.00 [-0.09, 0.09] | 0.00 [-0.09, 0.09] | -0.00 [-0.09, 0.09] | -0.00 [-0.09, 0.09] | 0.00 [-0.09, 0.09] | -0.00 [-0.09, 0.09] | -0.00 [-0.09, 0.09] | -0.00 [-0.09, 0.09] | 0.00 [-0.09, 0.09] |
| **Social:Interval:LBMP** | -0.00 [-0.09, 0.09] | -0.00 [-0.09, 0.09] | -0.00 [-0.09, 0.09] | 0.00 [-0.09, 0.09] | -0.00 [-0.09, 0.09] | 0.00 [-0.09, 0.09] | 0.00 [-0.09, 0.09] | -0.00 [-0.09, 0.09] | -0.00 [-0.09, 0.09] | -0.00 [-0.09, 0.09] |
| **Laughter Manipulation:Social:LBFI** | 0.00 [-0.09, 0.09] | 0.00 [-0.09, 0.09] | -0.00 [-0.09, 0.09] | 0.00 [-0.09, 0.09] | -0.00 [-0.09, 0.09] | -0.00 [-0.09, 0.09] | 0.00 [-0.09, 0.09] | 0.00 [-0.09, 0.09] | -0.00 [-0.09, 0.09] | -0.00 [-0.09, 0.09] |
| **Laughter Manipulation:Interval:LBFI** | -0.00 [-0.09, 0.09] | 0.00 [-0.09, 0.09] | -0.00 [-0.09, 0.09] | 0.00 [-0.09, 0.09] | 0.00 [-0.09, 0.09] | 0.00 [-0.10, 0.10] | -0.00 [-0.09, 0.09] | 0.00 [-0.09, 0.09] | -0.00 [-0.09, 0.09] | 0.00 [-0.09, 0.09] |
| **Social:Interval:LBFI** | -0.00 [-0.09, 0.09] | -0.00 [-0.09, 0.09] | 0.00 [-0.09, 0.09] | -0.00 [-0.10, 0.09] | 0.00 [-0.09, 0.09] | 0.00 [-0.09, 0.09] | -0.00 [-0.09, 0.09] | -0.00 [-0.09, 0.09] | 0.00 [-0.09, 0.09] | 0.00 [-0.09, 0.09] |
| **Laughter Manipulation:LBMP:LBFI** | -0.00 [-0.09, 0.09] | 0.00 [-0.09, 0.09] | 0.00 [-0.09, 0.09] | -0.00 [-0.09, 0.09] | 0.00 [-0.09, 0.09] | -0.00 [-0.09, 0.09] | -0.00 [-0.09, 0.09] | 0.00 [-0.09, 0.09] | 0.00 [-0.09, 0.09] | -0.00 [-0.09, 0.09] |
| **Social:LBMP:LBFI** | 0.00 [-0.09, 0.09] | -0.00 [-0.09, 0.09] | 0.00 [-0.09, 0.10] | 0.00 [-0.09, 0.09] | -0.00 [-0.09, 0.09] | 0.00 [-0.09, 0.09] | -0.00 [-0.09, 0.09] | 0.00 [-0.09, 0.09] | -0.00 [-0.09, 0.09] | 0.00 [-0.09, 0.09] |
| **Interval:LBMP:LBFI** | 0.00 [-0.09, 0.09] | -0.00 [-0.09, 0.09] | 0.00 [-0.09, 0.09] | -0.00 [-0.09, 0.09] | -0.00 [-0.09, 0.09] | -0.00 [-0.09, 0.09] | -0.00 [-0.09, 0.09] | -0.00 [-0.09, 0.09] | -0.00 [-0.09, 0.09] | 0.00 [-0.09, 0.09] |
| **Laughter Manipulation:Social:Interval:LBMP** | -0.00 [-0.09, 0.09] | -0.00 [-0.09, 0.09] | 0.00 [-0.10, 0.10] | 0.00 [-0.09, 0.09] | -0.00 [-0.09, 0.09] | 0.00 [-0.10, 0.09] | 0.00 [-0.09, 0.09] | -0.00 [-0.09, 0.09] | -0.00 [-0.09, 0.09] | 0.00 [-0.09, 0.09] |
| **Laughter Manipulation:Social:Interval:LBFI** | -0.00 [-0.09, 0.09] | -0.00 [-0.09, 0.09] | 0.00 [-0.09, 0.09] | 0.00 [-0.09, 0.09] | -0.00 [-0.10, 0.10] | 0.00 [-0.09, 0.09] | 0.00 [-0.09, 0.09] | 0.00 [-0.09, 0.09] | 0.00 [-0.09, 0.09] | -0.00 [-0.09, 0.09] |
| **Laughter Manipulation:Social:LBMP:LBFI** | -0.00 [-0.10, 0.09] | 0.00 [-0.09, 0.09] | 0.00 [-0.09, 0.09] | -0.00 [-0.09, 0.09] | -0.00 [-0.10, 0.09] | 0.00 [-0.09, 0.09] | 0.00 [-0.09, 0.10] | -0.00 [-0.09, 0.09] | -0.00 [-0.09, 0.09] | 0.00 [-0.09, 0.09] |
| **Laughter Manipulation:Interval:LBMP:LBFI** | -0.00 [-0.09, 0.09] | -0.00 [-0.09, 0.09] | 0.00 [-0.09, 0.09] | -0.00 [-0.09, 0.09] | 0.00 [-0.09, 0.09] | -0.00 [-0.09, 0.09] | 0.00 [-0.10, 0.09] | 0.00 [-0.09, 0.09] | 0.00 [-0.09, 0.09] | 0.00 [-0.09, 0.09] |
| **Social:Interval:LBMP:LBFI** | -0.00 [-0.09, 0.09] | 0.00 [-0.09, 0.09] | 0.00 [-0.09, 0.09] | -0.00 [-0.10, 0.09] | -0.00 [-0.09, 0.09] | -0.00 [-0.09, 0.09] | -0.00 [-0.09, 0.09] | 0.00 [-0.09, 0.09] | -0.00 [-0.09, 0.09] | 0.00 [-0.09, 0.09] |
| **Laughter Manipulation:Social:Interval:LBMP:LBFI** | 0.00 [-0.09, 0.09] | 0.00 [-0.09, 0.09] | 0.00 [-0.09, 0.09] | -0.00 [-0.10, 0.10] | 0.00 [-0.09, 0.09] | -0.00 [-0.09, 0.10] | -0.00 [-0.09, 0.09] | 0.00 [-0.09, 0.09] | -0.00 [-0.09, 0.09] | -0.00 [-0.09, 0.09] |

Table 14. Posterior Estimates for all Effects from the Bayesian parameter estimations by ROI Pair for synchrony measured during the free interaction phase for HbR data. Posterior means and 95% credible intervals (CI) are reported for all main effects and interactions separately for each ROI pair. ROI pairs included left (l) and right (r) inferior frontal gyrus (IFG) and the temporoparietal junction (TPJ). Laughter Behavior during the manipulation phase (LBMP) and Laughter Behavior during the free interaction phase (LBFI). Sample sizes for Interval 1 and Interval 2 refer to the number of WTC values; each dyad contributes one measure per interval, resulting in two per dyad overall. For heterogeneous ROI pairs, sample sizes are higher than for homologous pairs because each pair includes bidirectional synchrony (e.g., TPJl-IFGr and IFGr-TPJl), both of which are included together for one ROI-pair analysis.

| ROI | H1a_full vs H1a_null_manipulation | H1a_full vs H1a_null_LBMP | H1b_full vs H1a_full |
| --- | --- | --- | --- |
| IFGl_IFGl | 0.45 | 0.00 | 0.20 |
| IFGl_TPJl | 0.28 | 0.00 | 0.09 |
| IFGl_TPJr | 0.20 | 0.00 | 0.26 |
| IFGr_IFGl | 0.28 | 0.00 | 0.10 |
| IFGr_IFGr | 0.42 | 0.00 | 0.18 |
| IFGr_TPJl | 0.28 | 0.00 | 0.15 |
| IFGr_TPJr | 0.17 | 0.00 | 0.27 |
| TPJl_TPJl | 0.37 | 0.00 | 0.33 |
| TPJr_TPJl | 0.23 | 0.00 | 0.19 |
| TPJr_TPJr | 0.36 | 0.00 | 0.25 |

Table 15. This table reports Bayes factor comparisons for model comparisons testing Hypotheses 1a and 1b for synchrony during manipulation phase for HbR data. The H1b_full model includes all predictors and interactions (*Synchrony ∼ Laughter Manipulation*Social*Interval* LBMP (Laughter Behavior during Manipulation Phase)*. The H1a_full model excludes the key interaction term between Laughter (Manipulation, LBMP) and Social, focusing instead on main effects and simpler interactions.

| ROI | H1a_full vs H1a_null_manipulation | H1a_full vs H1a_null_LBMP | H1a_full vs H1a_null_LBFI | H1b_full vs H1a_full |
| --- | --- | --- | --- | --- |
| IFGl_IFGl | 0.50 | 0.00 | 0.01 | 0.38 |
| IFGl_TPJl | 0.41 | 0.00 | 0.00 | 0.12 |
| IFGl_TPJr | 0.97 | 0.00 | 0.00 | 0.12 |
| IFGr_IFGl | 0.41 | 0.00 | 0.00 | 0.22 |
| IFGr_IFGr | 0.58 | 0.00 | 0.26 | 0.33 |
| IFGr_TPJl | 0.43 | 0.00 | 0.00 | 0.18 |
| IFGr_TPJr | 0.24 | 0.00 | 0.00 | 0.22 |
| TPJl_TPJl | 0.35 | 0.00 | 0.00 | 0.38 |
| TPJr_TPJl | 0.56 | 0.00 | 0.00 | 0.39 |
| TPJr_TPJr | 0.63 | 0.00 | 0.02 | 0.25 |

Table 16. This table reports Bayes factor comparisons for model comparisons testing Hypotheses 1a and 1b for synchrony during the free interaction phase for HbR data. The H1b_full model includes all predictors and interactions (*Synchrony ∼ Laughter Manipulation*Social*Interval* LBMP (Laughter Behavior during Manipulation Phase)*LBFI (Laughter Behavior during Free Interaction).* H1a_full model excludes the key interaction term between Laughter (Manipulation, LBMP, LBFI) and Social, focusing instead on main effects and simpler interactions. Additionally, H1a_null_manipulation excludes Laughter Manipulation, H1a_null_LBMP excludes LBMP, and H1a_null_LBFI excludes LBFI to evaluate the contribution of these predictors to the full model. The Bayes factors quantify the relative evidence for the full models compared to the reduced models.

| Treatment: Condition  Outcome: Liking | | | | | |
| --- | --- | --- | --- | --- | --- |
| ROI Pair | Direct Effect | Indirect Effect | Mediator Effect | Total Effect | Proportion Mediated |
| **IFGl_IFGl** | -0.44[-1.21, 0.34] | 0.11[-0.06, 0.40] | -10.97[-20.15, -1.75] | -0.31[-1.11, 0.50] | -35.32[-470.16, 399.50] |
| **IFGl_TPJl** | -0.35[-1.13, 0.43] | -0.00[-0.15, 0.07] | -3.80[-14.70, 7.34] | -0.36[-1.16, 0.41] | 1.55[-83.81, 86.90] |
| **IFGl_TPJr** | -0.36[-1.15, 0.41] | 0.00[-0.08, 0.12] | 3.43[-9.66, 16.51] | -0.35[-1.15, 0.41] | -0.40[-84.09, 83.29] |
| **IFGr_IFGl** | -0.35[-1.12, 0.40] | 0.00[-0.11, 0.18] | -6.71[-17.96, 4.61] | -0.34[-1.12, 0.43] | -2.31[-137.80, 133.18] |
| **IFGr_IFGr** | -0.19[-1.01, 0.62] | -0.02[-0.23, 0.07] | 4.88[-3.57, 13.39] | -0.23[-1.05, 0.60] | 10.11[-151.44, 171.68] |
| **IFGr_TPJl** | -0.19[-0.98, 0.61] | -0.00[-0.17, 0.13] | -1.03[-11.99, 9.34] | -0.21[-0.99, 0.59] | 1.42[-158.58, 161.43] |
| **IFGr_TPJr** | -0.17[-0.99, 0.62] | 0.00[-0.13, 0.14] | -0.15[-10.59, 10.84] | -0.17[-0.99, 0.62] | -0.01[-150.18, 150.15] |
| **TPJl_TPJl** | -0.17[-1.14, 0.76] | 0.00[-0.13, 0.15] | 0.39[-9.04, 9.95] | -0.18[-1.14, 0.75] | -0.16[-138.94, 138.62] |
| **TPJr_TPJl** | 0.01[-0.82, 0.88] | 0.00[-0.19, 0.21] | 0.72[-11.17, 12.25] | 0.01[-0.81, 0.86] | 21.55[-214.94, 258.07] |
| **TPJr_TPJr** | 0.15[-0.75, 1.05] | 0.00[-0.12, 0.14] | -1.83[-10.98, 7.43] | 0.16[-0.75, 1.06] | 0.85[-112.87, 114.59] |
| Treatment: Laughter Behavior during Manipulation phase  Outcome: Liking | | | | | |
| ROI Pair | Direct Effect | Indirect Effect | Mediator Effect | Total Effect | Proportion Mediated |
| **IFGl_IFGl** | 5420.69[-1578.94, 12915.71] | -1393.64[-4863.07, 1067.22] | -14.90[-24.48, -5.77] | 3934.81[-3693.26, 11788.37] | -35.42[-491.74, 420.91] |
| **IFGl_TPJl** | 4555.14[-3233.53, 12171.84] | -691.52[-3434.19, 559.08] | -8.16[-20.42, 3.79] | 3676.55[-4168.41, 11451.19] | -18.80[-304.87, 267.25] |
| **IFGl_TPJr** | 3579.07[-3867.23, 10877.19] | 28.24[-956.19, 1330.03] | 2.83[-10.52, 17.03] | 3663.99[-3850.42, 10985.44] | 0.77[-91.34, 92.89] |
| **IFGr_IFGl** | 4867.57[-3182.80, 12420.89] | -951.45[-3632.02, 529.38] | -10.59[-22.32, 1.09] | 3768.48[-4217.11, 11389.48] | -25.25[-393.83, 343.33] |
| **IFGr_IFGr** | 2772.43[-5331.17, 10915.25] | 364.61[-1108.27, 2788.95] | 3.63[-5.72, 12.77] | 3335.07[-4741.64, 11391.97] | 10.92[-193.25, 215.12] |
| **IFGr_TPJl** | 3860.12[-4566.15, 12168.79] | -375.16[-2946.05, 956.27] | -4.77[-16.15, 6.55] | 3265.40[-5010.66, 11597.21] | -11.49[-260.08, 237.11] |
| **IFGr_TPJr** | 4332.90[-3649.19, 11997.23] | -247.77[-2927.82, 2073.25] | -1.76[-13.21, 9.24] | 3921.92[-3864.95, 11388.09] | -6.32[-222.75, 210.12] |
| **TPJl_TPJl** | 4827.99[-6204.06, 15430.69] | -9.93[-1759.05, 1637.53] | -1.21[-11.30, 8.88] | 4772.31[-6382.98, 15361.24] | -0.20[-109.48, 109.06] |
| **TPJr_TPJl** | 6131.69[-2813.57, 14441.25] | -402.66[-3742.85, 1957.20] | -2.97[-15.45, 9.65] | 5483.32[-3216.30, 13609.11] | -7.33[-186.12, 171.43] |
| **TPJr_TPJr** | 6497.93[-1242.78, 14231.96] | 3.47[-1343.31, 1416.57] | 0.44[-8.82, 9.77] | 6500.19[-1193.21, 14204.91] | 0.05[-54.17, 54.28] |
| Treatment: Laughter Behavior during Free Interaction phase  Outcome: Liking | | | | | |
| ROI Pair | Direct Effect | Indirect Effect | Mediator Effect | Total Effect | Proportion Mediated |
| **IFGl_IFGl** | 12217.37[4314.68, 19664.36] | 659.71[-1128.02, 3352.34] | -10.49[-19.03, -1.61] | 13068.23[4860.40, 20952.60] | 5.04[-15.15, 25.25] |
| **IFGl_TPJl** | 12571.50[4456.79, 20373.96] | 193.00[-1271.39, 2309.45] | -2.40[-13.38, 8.46] | 12914.50[4792.96, 20417.26] | 1.48[-15.17, 18.17] |
| **IFGl_TPJr** | 13310.20[5611.52, 21562.57] | -250.25[-2434.99, 817.78] | 5.32[-6.30, 17.67] | 12905.00[4987.11, 21045.72] | -1.93[-17.60, 13.72] |
| **IFGr_IFGl** | 13226.68[5462.00, 20765.55] | -235.92[-2328.06, 1175.97] | -9.05[-19.67, 1.75] | 12839.53[4990.95, 20678.42] | -1.84[-19.96, 16.28] |
| **IFGr_IFGr** | 13743.78[5796.85, 21879.44] | 48.20[-923.75, 1690.19] | 2.70[-5.30, 11.14] | 13893.82[5930.36, 21953.29] | 0.34[-9.91, 10.59] |
| **IFGr_TPJl** | 13911.12[6400.26, 21935.43] | -23.11[-1584.84, 1298.72] | 0.70[-8.87, 10.74] | 13854.86[6332.58, 21824.41] | -0.17[-11.57, 11.23] |
| **IFGr_TPJr** | 13381.37[5529.44, 21086.20] | -1.43[-1015.24, 1103.56] | 0.40[-9.87, 10.44] | 13403.47[5489.92, 21049.03] | -0.01[-8.98, 8.94] |
| **TPJl_TPJl** | 15172.11[6823.12, 23299.95] | -9.39[-1399.34, 1170.26] | -1.21[-9.45, 7.23] | 15163.09[6669.44, 23252.19] | -0.05[-9.92, 9.80] |
| **TPJr_TPJl** | 12884.47[4959.50, 20622.63] | -3.40[-1133.19, 1061.59] | 0.34[-10.29, 11.10] | 12852.47[4964.70, 20491.55] | -0.02[-10.50, 10.46] |
| **TPJr_TPJr** | 10533.83[2638.24, 18173.73] | -46.20[-1869.19, 1387.76] | 1.13[-7.52, 9.74] | 10402.95[2525.51, 18194.97] | -0.44[-21.16, 20.26] |
| Treatment: Condition  Outcome: Prosociality (Money Sharing) | | | | | |
| ROI Pair | Direct Effect | Indirect Effect | Mediator Effect | Total Effect | Proportion Mediated |
| **IFGl_IFGl** | -0.06[-0.22, 0.09] | -0.00[-0.04, 0.01] | 0.77[-1.10, 2.61] | -0.07[-0.23, 0.08] | 7.62[-124.07, 139.31] |
| **IFGl_TPJl** | -0.08[-0.23, 0.08] | 0.00[-0.01, 0.03] | 0.86[-1.43, 3.19] | -0.07[-0.24, 0.08] | -2.35[-91.64, 86.90] |
| **IFGl_TPJr** | -0.07[-0.23, 0.09] | -0.00[-0.02, 0.01] | -0.52[-3.05, 2.05] | -0.07[-0.23, 0.08] | 0.44[-71.31, 72.18] |
| **IFGr_IFGl** | -0.08[-0.23, 0.07] | -0.00[-0.02, 0.01] | 0.83[-1.27, 2.94] | -0.08[-0.23, 0.07] | 0.92[-79.60, 81.45] |
| **IFGr_IFGr** | -0.08[-0.25, 0.07] | -0.00[-0.03, 0.02] | 0.38[-1.21, 2.08] | -0.09[-0.25, 0.07] | 1.23[-80.35, 82.82] |
| **IFGr_TPJl** | -0.10[-0.26, 0.06] | 0.00[-0.01, 0.05] | 1.12[-1.10, 3.31] | -0.09[-0.25, 0.07] | -7.25[-168.47, 153.99] |
| **IFGr_TPJr** | -0.09[-0.26, 0.07] | 0.00[-0.01, 0.04] | 1.19[-1.01, 3.26] | -0.08[-0.25, 0.07] | -7.01[-169.28, 155.24] |
| **TPJl_TPJl** | -0.11[-0.30, 0.07] | 0.00[-0.03, 0.05] | 1.04[-0.72, 2.82] | -0.10[-0.30, 0.08] | -2.14[-127.15, 122.87] |
| **TPJr_TPJl** | -0.10[-0.28, 0.07] | 0.02[-0.01, 0.09] | 2.06[-0.26, 4.35] | -0.07[-0.26, 0.10] | -29.14[-418.30, 359.99] |
| **TPJr_TPJr** | -0.10[-0.30, 0.10] | 0.00[-0.03, 0.03] | -0.84[-2.86, 1.24] | -0.10[-0.30, 0.10] | -0.39[-96.26, 95.48] |
| Treatment: Laughter Behavior during Manipulation phase  Outcome: Prosociality (Money Sharing) | | | | | |
| ROI Pair | Direct Effect | Indirect Effect | Mediator Effect | Total Effect | Proportion Mediated |
| **IFGl_IFGl** | 875.28[-543.58, 2302.83] | 7.41[-236.44, 305.38] | 0.24[-1.61, 2.11] | 894.68[-545.25, 2294.42] | 0.82[-86.35, 88.01] |
| **IFGl_TPJl** | 936.14[-472.57, 2466.76] | -5.64[-346.95, 293.48] | -0.12[-2.39, 2.14] | 915.35[-493.32, 2448.11] | -0.61[-111.04, 109.81] |
| **IFGl_TPJr** | 968.53[-543.26, 2414.16] | -9.19[-290.90, 165.37] | -0.72[-3.37, 1.81] | 935.44[-588.33, 2403.48] | -0.97[-75.04, 73.07] |
| **IFGr_IFGl** | 782.55[-654.89, 2238.01] | 29.90[-218.54, 402.58] | 0.50[-1.69, 2.62] | 829.56[-604.58, 2298.20] | 3.60[-108.40, 115.62] |
| **IFGr_IFGr** | 578.49[-1056.80, 2131.84] | 1.50[-350.19, 376.52] | 0.05[-1.71, 1.82] | 591.41[-1036.70, 2112.21] | 0.25[-159.86, 160.37] |
| **IFGr_TPJl** | 477.60[-1169.32, 2149.36] | 49.08[-205.32, 474.19] | 0.67[-1.45, 2.78] | 548.08[-1064.04, 2204.21] | 8.96[-192.43, 210.34] |
| **IFGr_TPJr** | 579.60[-1013.20, 2137.81] | 152.59[-227.28, 731.30] | 0.98[-1.17, 3.09] | 745.48[-738.94, 2295.68] | 20.46[-230.81, 271.75] |
| **TPJl_TPJl** | 755.66[-1358.17, 2952.81] | 5.71[-329.66, 455.16] | 0.52[-1.49, 2.53] | 779.76[-1393.44, 2964.44] | 0.72[-119.71, 121.18] |
| **TPJr_TPJl** | 648.42[-1021.88, 2433.88] | 216.57[-195.23, 956.61] | 1.39[-1.04, 3.82] | 912.80[-790.75, 2664.52] | 23.73[-246.62, 294.07] |
| **TPJr_TPJr** | 1014.89[-836.12, 2912.38] | -9.84[-442.83, 266.99] | -0.50[-2.62, 1.65] | 986.66[-895.84, 2885.03] | -1.00[-123.45, 121.45] |
| Treatment: Laughter Behavior during Free Interaction phase  Outcome: Prosociality (Money Sharing) | | | | | |
| ROI Pair | Direct Effect | Indirect Effect | Mediator Effect | Total Effect | Proportion Mediated |
| **IFGl_IFGl** | 1342.96[-173.79, 2902.50] | -6.02[-279.18, 196.45] | 0.23[-1.55, 1.98] | 1333.12[-186.86, 2886.19] | -0.45[-40.24, 39.32] |
| **IFGl_TPJl** | 1434.29[-90.03, 2967.57] | -72.81[-530.18, 187.32] | 0.86[-1.26, 3.02] | 1325.71[-194.08, 2830.58] | -5.49[-96.17, 85.18] |
| **IFGl_TPJr** | 1271.11[-229.97, 2819.21] | 14.92[-226.53, 353.18] | -0.46[-2.81, 1.86] | 1303.76[-179.65, 2873.51] | 1.13[-41.96, 44.25] |
| **IFGr_IFGl** | 1302.25[-217.68, 2865.24] | 4.64[-184.60, 247.99] | 0.36[-1.62, 2.43] | 1316.78[-207.22, 2872.99] | 0.34[-32.31, 33.00] |
| **IFGr_IFGr** | 1422.19[-185.00, 3063.15] | 2.06[-204.72, 229.31] | 0.15[-1.39, 1.67] | 1431.61[-195.11, 3069.26] | 0.14[-31.92, 32.21] |
| **IFGr_TPJl** | 1510.15[34.93, 2999.72] | -96.60[-578.25, 128.09] | 1.43[-0.67, 3.49] | 1379.62[-101.61, 2879.36] | -7.00[-80.59, 66.59] |
| **IFGr_TPJr** | 1306.86[-201.94, 2801.01] | -12.64[-349.95, 219.11] | 1.04[-0.90, 2.94] | 1269.16[-259.60, 2807.19] | -1.00[-60.86, 58.88] |
| **TPJl_TPJl** | 1250.81[-512.90, 2954.55] | 2.27[-284.93, 333.95] | 0.75[-0.88, 2.40] | 1270.49[-570.34, 3013.48] | 0.17[-58.82, 59.17] |
| **TPJr_TPJl** | 1292.37[-301.41, 2921.91] | -52.80[-596.53, 366.65] | 2.32[0.12, 4.41] | 1214.53[-435.23, 2903.36] | -4.34[-145.37, 136.68] |
| **TPJr_TPJr** | 1093.17[-755.14, 2993.91] | 4.51[-359.57, 393.69] | -0.08[-2.11, 1.94] | 1108.39[-714.58, 3014.73] | 0.40[-100.60, 101.43] |
| Treatment: Condition  Outcome: Prosociality (Helping) | | | | | |
| ROI Pair | Direct Effect | Indirect Effect | Mediator Effect | Total Effect | Proportion Mediated |
| **IFGl_IFGl** | 0.00[-0.12, 0.13] | -0.01[-0.05, 0.00] | 1.21[-0.28, 2.72] | -0.00[-0.13, 0.11] | 149.71[-189.46, 488.91] |
| **IFGl_TPJl** | -0.01[-0.14, 0.11] | -0.00[-0.02, 0.01] | -0.56[-2.46, 1.26] | -0.01[-0.14, 0.11] | 5.34[-112.46, 123.17] |
| **IFGl_TPJr** | -0.01[-0.14, 0.11] | 0.00[-0.01, 0.01] | 0.05[-1.98, 2.06] | -0.01[-0.14, 0.11] | -0.10[-103.04, 102.84] |
| **IFGr_IFGl** | -0.00[-0.13, 0.12] | -0.00[-0.02, 0.01] | 1.06[-0.63, 2.79] | -0.00[-0.14, 0.11] | 16.21[-157.28, 189.71] |
| **IFGr_IFGr** | -0.00[-0.13, 0.13] | -0.00[-0.02, 0.01] | 0.11[-1.26, 1.44] | -0.00[-0.14, 0.13] | 2.33[-134.50, 139.16] |
| **IFGr_TPJl** | -0.02[-0.15, 0.09] | 0.01[-0.01, 0.05] | 1.53[-0.18, 3.25] | -0.01[-0.14, 0.12] | -95.00[-527.74, 337.74] |
| **IFGr_TPJr** | -0.01[-0.14, 0.12] | 0.00[-0.01, 0.03] | 0.88[-0.81, 2.62] | -0.00[-0.13, 0.12] | -69.29[-290.97, 152.36] |
| **TPJl_TPJl** | -0.00[-0.15, 0.14] | 0.00[-0.02, 0.02] | 0.18[-1.29, 1.67] | -0.00[-0.15, 0.14] | -3.20[-121.68, 115.26] |
| **TPJr_TPJl** | -0.00[-0.13, 0.13] | 0.01[-0.01, 0.05] | 1.04[-0.75, 2.85] | 0.01[-0.12, 0.15] | 88.65[-244.09, 421.41] |
| **TPJr_TPJr** | -0.00[-0.15, 0.15] | 0.00[-0.02, 0.02] | -0.22[-1.85, 1.41] | -0.00[-0.15, 0.16] | -21.01[-163.08, 121.04] |
| Treatment: Laughter Behavior during Manipulation phase  Outcome: Prosociality (Helping) | | | | | |
| ROI Pair | Direct Effect | Indirect Effect | Mediator Effect | Total Effect | Proportion Mediated |
| **IFGl_IFGl** | 236.46[-971.86, 1461.73] | 59.86[-114.63, 397.66] | 0.88[-0.61, 2.43] | 332.04[-907.88, 1548.49] | 18.03[-205.86, 241.91] |
| **IFGl_TPJl** | 356.52[-805.21, 1540.49] | -77.20[-469.32, 133.34] | -1.02[-2.89, 0.95] | 241.25[-881.26, 1430.30] | -32.00[-337.22, 273.22] |
| **IFGl_TPJr** | 217.72[-954.57, 1368.57] | 7.58[-140.58, 241.81] | 0.61[-1.50, 2.72] | 241.77[-949.21, 1414.87] | 3.14[-155.18, 161.46] |
| **IFGr_IFGl** | 248.85[-919.52, 1434.64] | 58.28[-136.25, 392.54] | 0.78[-1.01, 2.52] | 322.83[-829.94, 1506.89] | 18.05[-244.66, 280.77] |
| **IFGr_IFGr** | 206.12[-1123.03, 1524.87] | 6.10[-278.92, 323.81] | 0.09[-1.39, 1.60] | 223.00[-1117.00, 1537.23] | 2.74[-200.25, 205.74] |
| **IFGr_TPJl** | 27.30[-1283.29, 1254.68] | 101.26[-97.51, 535.58] | 1.12[-0.69, 2.89] | 151.96[-1141.06, 1415.19] | 66.64[-255.09, 388.36] |
| **IFGr_TPJr** | 161.25[-1093.37, 1434.05] | 165.65[-140.65, 657.63] | 1.01[-0.76, 2.81] | 348.67[-857.44, 1595.70] | 47.50[-425.70, 520.73] |
| **TPJl_TPJl** | 84.88[-1563.21, 1757.84] | 1.48[-237.51, 283.40] | 0.17[-1.33, 1.70] | 94.32[-1571.35, 1764.36] | 1.58[-133.52, 136.68] |
| **TPJr_TPJl** | 234.93[-1144.09, 1624.30] | 200.18[-131.40, 797.39] | 1.21[-0.67, 3.15] | 476.43[-919.11, 1832.16] | 42.02[-468.76, 552.79] |
| **TPJr_TPJr** | 560.18[-886.72, 2027.71] | 3.04[-218.18, 291.88] | 0.28[-1.40, 1.95] | 569.84[-901.54, 2023.44] | 0.53[-109.93, 111.01] |
| Treatment: Laughter Behavior during Free Interaction phase  Outcome: Prosociality (Helping) | | | | | |
| ROI Pair | Direct Effect | Indirect Effect | Mediator Effect | Total Effect | Proportion Mediated |
| **IFGl_IFGl** | 1021.97[-261.20, 2276.16] | -63.53[-434.50, 140.09] | 1.12[-0.35, 2.70] | 934.41[-368.86, 2243.83] | -6.79[-124.79, 111.20] |
| **IFGl_TPJl** | 820.94[-481.56, 2164.53] | 35.92[-214.64, 385.83] | -0.48[-2.38, 1.47] | 881.45[-419.94, 2185.06] | 4.08[-95.15, 103.29] |
| **IFGl_TPJr** | 913.30[-467.94, 2259.37] | -5.30[-264.17, 220.69] | 0.17[-1.90, 2.17] | 898.60[-475.44, 2232.65] | -0.58[-79.09, 77.92] |
| **IFGr_IFGl** | 867.75[-416.66, 2200.55] | 16.51[-161.57, 306.08] | 0.89[-0.85, 2.70] | 903.37[-362.68, 2255.52] | 1.83[-66.85, 70.51] |
| **IFGr_IFGr** | 1165.92[-262.13, 2498.20] | -0.84[-210.05, 181.74] | -0.07[-1.46, 1.32] | 1163.85[-266.48, 2504.02] | -0.07[-38.28, 38.14] |
| **IFGr_TPJl** | 1188.58[-134.25, 2584.85] | -140.02[-629.67, 155.47] | 1.79[0.14, 3.50] | 1029.23[-315.10, 2437.06] | -13.59[-250.87, 223.65] |
| **IFGr_TPJr** | 972.38[-395.12, 2338.40] | -12.81[-300.13, 187.91] | 0.86[-0.82, 2.59] | 943.00[-437.07, 2313.79] | -1.36[-81.95, 79.23] |
| **TPJl_TPJl** | 1065.27[-410.27, 2573.96] | -0.20[-237.80, 241.76] | 0.09[-1.43, 1.58] | 1063.17[-453.77, 2565.29] | -0.02[-53.14, 53.10] |
| **TPJr_TPJl** | 1064.31[-315.52, 2457.98] | -19.81[-365.47, 233.28] | 1.21[-0.64, 3.05] | 1033.79[-363.85, 2448.70] | -1.91[-87.90, 84.06] |
| **TPJr_TPJr** | 1367.13[-215.37, 2917.41] | -6.77[-338.73, 263.85] | 0.14[-1.50, 1.75] | 1346.09[-239.35, 2904.94] | -0.50[-52.06, 51.06] |
| Treatment: Condition  Outcome: Bonding | | | | | |
| ROI Pair | Direct Effect | Indirect Effect | Mediator Effect | Total Effect | Proportion Mediated |
| **IFGl_IFGl** | -0.24[-0.87, 0.37] | 0.00[-0.11, 0.12] | -0.23[-7.70, 7.15] | -0.24[-0.87, 0.38] | -0.23[-131.77, 131.31] |
| **IFGl_TPJl** | -0.25[-0.90, 0.38] | -0.00[-0.10, 0.07] | -1.64[-10.61, 7.32] | -0.26[-0.91, 0.37] | 0.82[-92.95, 94.62] |
| **IFGl_TPJr** | -0.26[-0.92, 0.38] | 0.00[-0.07, 0.10] | 3.33[-7.01, 13.59] | -0.26[-0.92, 0.38] | -0.50[-95.90, 94.90] |
| **IFGr_IFGl** | -0.23[-0.84, 0.39] | 0.00[-0.07, 0.08] | -0.27[-8.84, 8.45] | -0.23[-0.85, 0.38] | -0.13[-77.59, 77.31] |
| **IFGr_IFGr** | -0.20[-0.88, 0.45] | -0.03[-0.24, 0.08] | 6.20[-0.72, 12.88] | -0.25[-0.93, 0.41] | 15.34[-200.03, 230.71] |
| **IFGr_TPJl** | -0.13[-0.80, 0.51] | -0.04[-0.26, 0.06] | -6.70[-15.28, 1.90] | -0.20[-0.87, 0.46] | 23.48[-256.86, 303.83] |
| **IFGr_TPJr** | -0.25[-0.92, 0.41] | 0.02[-0.06, 0.20] | 5.00[-3.58, 13.87] | -0.20[-0.88, 0.45] | -13.36[-253.40, 226.66] |
| **TPJl_TPJl** | -0.01[-0.79, 0.74] | 0.00[-0.11, 0.15] | 2.49[-4.60, 9.74] | -0.00[-0.79, 0.77] | -28.28[-174.25, 117.70] |
| **TPJr_TPJl** | -0.06[-0.76, 0.65] | 0.00[-0.14, 0.19] | 1.16[-8.57, 10.40] | -0.05[-0.75, 0.67] | -15.31[-259.06, 228.44] |
| **TPJr_TPJr** | -0.09[-0.86, 0.67] | -0.00[-0.10, 0.11] | -0.19[-8.14, 7.69] | -0.09[-0.87, 0.66] | 0.17[-125.39, 125.73] |
| Treatment: Laughter Behavior during Manipulation phase  Outcome: Bonding | | | | | |
| ROI Pair | Direct Effect | Indirect Effect | Mediator Effect | Total Effect | Proportion Mediated |
| **IFGl_IFGl** | 1244.34[-4848.81, 7125.02] | -167.04[-1748.15, 693.22] | -2.76[-10.56, 5.36] | 966.73[-5246.28, 6781.28] | -17.28[-206.61, 172.05] |
| **IFGl_TPJl** | 1020.31[-5381.57, 7650.39] | -229.71[-1998.74, 892.59] | -3.30[-12.94, 6.88] | 678.11[-5530.66, 7190.38] | -33.88[-264.06, 196.31] |
| **IFGl_TPJr** | 520.74[-5725.31, 6800.22] | 62.68[-809.19, 1345.28] | 4.81[-6.32, 15.75] | 624.71[-5704.98, 7056.97] | 10.02[-163.99, 184.06] |
| **IFGr_IFGl** | 1186.17[-5299.90, 7427.94] | -160.49[-1786.65, 948.56] | -2.51[-12.09, 7.31] | 893.31[-5562.17, 7101.56] | -17.96[-238.00, 202.06] |
| **IFGr_IFGr** | -12.95[-6948.59, 7312.94] | 587.11[-578.23, 3013.52] | 5.13[-2.60, 12.91] | 792.72[-6144.73, 7918.68] | 74.06[-280.95, 429.08] |
| **IFGr_TPJl** | 2171.69[-4425.62, 8770.83] | -838.90[-3324.90, 471.60] | -8.33[-17.68, 0.47] | 1187.67[-5589.38, 7758.79] | -70.62[-494.50, 353.23] |
| **IFGr_TPJr** | 25.81[-6570.27, 6527.27] | 734.81[-975.22, 3286.76] | 4.50[-5.13, 13.92] | 926.87[-5670.16, 7410.37] | 79.28[-441.19, 599.75] |
| **TPJl_TPJl** | 6078.34[-2753.72, 15221.12] | -1.53[-1447.86, 1259.66] | -0.10[-8.47, 8.19] | 6055.31[-2871.28, 15300.97] | -0.02[-61.00, 60.95] |
| **TPJr_TPJl** | 4540.46[-2680.49, 12019.12] | -262.07[-2738.65, 1622.66] | -1.98[-12.07, 8.07] | 4187.34[-2791.35, 11506.88] | -6.25[-197.75, 185.22] |
| **TPJr_TPJr** | 4161.72[-2902.42, 11561.79] | -16.68[-1461.09, 1130.04] | -1.07[-9.46, 7.76] | 4082.75[-2999.90, 11533.73] | -0.40[-88.57, 87.76] |
| Treatment: Laughter Behavior during Free Interaction phase  Outcome: Bonding | | | | | |
| ROI Pair | Direct Effect | Indirect Effect | Mediator Effect | Total Effect | Proportion Mediated |
| **IFGl_IFGl** | 7451.76[914.86, 14235.12] | -13.41[-1112.17, 854.14] | 0.72[-6.49, 7.98] | 7320.27[683.04, 14034.28] | -0.17[-21.32, 20.96] |
| **IFGl_TPJl** | 7124.67[505.19, 14011.61] | 85.61[-1177.10, 1650.08] | -1.36[-9.97, 7.81] | 7245.77[600.47, 14109.54] | 1.17[-31.26, 33.61] |
| **IFGl_TPJr** | 7743.57[974.60, 14465.58] | -209.61[-1957.52, 653.42] | 4.61[-5.58, 15.05] | 7343.14[551.13, 14062.37] | -2.85[-37.71, 32.00] |
| **IFGr_IFGl** | 7422.63[941.71, 13807.98] | -10.88[-1058.70, 746.69] | -1.35[-10.12, 7.14] | 7329.26[860.37, 13907.49] | -0.14[-18.57, 18.28] |
| **IFGr_IFGr** | 7066.08[-50.97, 13885.00] | 184.56[-1147.78, 1951.69] | 5.36[-1.54, 12.32] | 7395.50[285.67, 14144.75] | 2.50[-33.03, 38.02] |
| **IFGr_TPJl** | 7137.67[528.67, 13660.02] | 428.95[-595.05, 2639.36] | -6.28[-15.33, 2.56] | 7685.61[970.76, 14349.80] | 5.58[-25.80, 36.96] |
| **IFGr_TPJr** | 7800.96[885.09, 14600.35] | -81.03[-1623.53, 1045.25] | 5.32[-3.33, 13.80] | 7648.95[545.21, 14596.41] | -1.06[-30.28, 28.17] |
| **TPJl_TPJl** | 7013.75[-416.89, 14466.17] | 6.84[-1033.49, 1129.55] | 1.39[-5.63, 8.60] | 7055.38[-378.74, 14455.10] | 0.10[-31.35, 31.55] |
| **TPJr_TPJl** | 7138.71[515.14, 13836.61] | -2.25[-1043.86, 970.04] | 0.30[-8.91, 9.15] | 7146.10[364.82, 13908.75] | -0.02[-23.96, 23.91] |
| **TPJr_TPJr** | 6277.95[-1220.23, 13873.11] | -53.54[-1680.87, 1197.09] | 1.51[-6.37, 9.24] | 6065.26[-1478.05, 13534.37] | -0.88[-58.21, 56.43] |

Table 17. Posterior estimates from Bayesian mediation models for Synchrony during the manipulation phase for HbR data. Social outcome variables (Liking, Prosociality (Money Sharing, and Helping), and Bonding) by ROI Pair. Separate models were computed for each ROI pair (as the mediator) with either the Laughter Condition (Laughter experience vs. no Laughter experience) or the Laughter Behavior for both time points of the experiment (during the manipulation phase, and during the free interaction phase) as the predictor (treatment). Posterior means and 95% Equal-Tailed Intervals (ETIs) are reported.

| Treatment: Condition  Outcome: Liking | | | | | |
| --- | --- | --- | --- | --- | --- |
| ROI Pair | Direct Effect | Indirect Effect | Mediator Effect | Total Effect | Proportion Mediated |
| **IFGl_IFGl** | -0.15[-0.97, 0.67] | -0.05[-0.31, 0.12] | 8.07[0.11, 15.96] | -0.22[-1.10, 0.60] | 22.21[-236.71, 281.13] |
| **IFGl_TPJl** | -0.27[-1.09, 0.54] | -0.02[-0.24, 0.09] | 8.11[-3.08, 18.87] | -0.31[-1.14, 0.51] | 8.66[-154.87, 172.21] |
| **IFGl_TPJr** | -0.30[-1.15, 0.52] | 0.03[-0.12, 0.25] | -2.52[-11.72, 6.28] | -0.27[-1.09, 0.56] | -11.24[-240.15, 217.68] |
| **IFGr_IFGl** | -0.36[-1.22, 0.44] | 0.05[-0.08, 0.30] | 8.06[-1.62, 18.14] | -0.29[-1.14, 0.53] | -19.05[-294.10, 255.97] |
| **IFGr_IFGr** | -0.13[-1.08, 0.84] | 0.01[-0.20, 0.28] | 0.97[-7.95, 10.20] | -0.10[-1.04, 0.84] | -12.25[-283.18, 258.67] |
| **IFGr_TPJl** | -0.06[-0.93, 0.77] | 0.02[-0.10, 0.23] | 3.33[-6.85, 13.63] | -0.03[-0.89, 0.79] | -55.29[-260.54, 149.93] |
| **IFGr_TPJr** | -0.08[-0.91, 0.74] | -0.00[-0.13, 0.10] | -2.53[-13.31, 8.05] | -0.09[-0.92, 0.75] | 1.08[-126.20, 128.36] |
| **TPJl_TPJl** | -0.12[-1.15, 0.93] | 0.01[-0.17, 0.29] | 6.52[-5.34, 17.92] | -0.09[-1.11, 1.01] | -13.22[-227.96, 201.53] |
| **TPJr_TPJl** | 0.08[-0.83, 1.00] | 0.05[-0.12, 0.35] | -5.04[-16.37, 6.87] | 0.15[-0.75, 1.08] | 33.77[-298.07, 365.62] |
| **TPJr_TPJr** | 0.23[-0.64, 1.14] | -0.01[-0.26, 0.12] | 3.88[-4.35, 11.98] | 0.20[-0.70, 1.12] | -8.46[-192.03, 175.12] |
| Treatment: Laughter Behavior during Free Interaction phase  Outcome: Liking | | | | | |
| ROI Pair | Direct Effect | Indirect Effect | Mediator Effect | Total Effect | Proportion Mediated |
| **IFGl_IFGl** | 12535.06[4164.31, 21098.32] | 649.20[-1220.96, 3539.55] | 8.01[0.44, 15.55] | 13340.54[4772.94, 22093.25] | 4.87[-15.75, 25.48] |
| **IFGl_TPJl** | 12518.07[4624.14, 20588.12] | 148.84[-1113.82, 2219.66] | 7.21[-3.26, 17.41] | 12879.86[4726.59, 20890.44] | 1.15[-13.91, 16.23] |
| **IFGl_TPJr** | 12318.40[4285.51, 20046.82] | -1.21[-1083.64, 1034.53] | -0.33[-8.95, 8.35] | 12302.68[4263.02, 20120.46] | -0.00[-10.22, 10.19] |
| **IFGr_IFGl** | 12521.15[3965.29, 20689.40] | -113.68[-2325.24, 1677.21] | 9.39[-0.47, 18.96] | 12297.66[3515.32, 20625.28] | -0.92[-23.87, 22.03] |
| **IFGr_IFGr** | 11590.38[2909.65, 20583.37] | -181.22[-2704.06, 1854.05] | -1.44[-9.80, 7.38] | 11318.86[2519.52, 19998.72] | -1.60[-32.59, 29.39] |
| **IFGr_TPJl** | 12460.30[4115.08, 20751.37] | 16.78[-1255.29, 1665.24] | 3.93[-6.25, 13.81] | 12455.41[3808.99, 20927.31] | 0.13[-14.00, 14.27] |
| **IFGr_TPJr** | 13020.98[4863.23, 21393.20] | 15.30[-1028.28, 1376.19] | -1.90[-11.59, 7.53] | 13070.76[4853.36, 21405.57] | 0.11[-11.08, 11.31] |
| **TPJl_TPJl** | 16411.87[6608.54, 26461.21] | 34.91[-1547.30, 2123.47] | 3.53[-6.70, 13.93] | 16642.06[6882.41, 26579.48] | 0.20[-12.64, 13.06] |
| **TPJr_TPJl** | 13319.52[4199.86, 22735.91] | -525.48[-3360.37, 938.12] | -6.66[-16.92, 3.60] | 12603.44[3353.53, 22244.29] | -4.16[-31.53, 23.19] |
| **TPJr_TPJr** | 11571.65[2329.42, 20493.16] | -481.46[-3712.80, 1497.91] | 6.01[-1.89, 13.81] | 10907.11[1524.48, 19775.23] | -4.41[-48.85, 40.02] |
| Treatment: Condition  Outcome: Prosociality (Money Sharing) | | | | | |
| ROI Pair | Direct Effect | Indirect Effect | Mediator Effect | Total Effect | Proportion Mediated |
| **IFGl_IFGl** | -0.07[-0.24, 0.09] | -0.00[-0.04, 0.01] | 0.97[-0.65, 2.57] | -0.08[-0.25, 0.09] | 5.65[-131.34, 142.62] |
| **IFGl_TPJl** | -0.07[-0.24, 0.09] | 0.00[-0.02, 0.02] | 0.03[-2.19, 2.27] | -0.07[-0.24, 0.09] | -0.02[-92.43, 92.39] |
| **IFGl_TPJr** | -0.09[-0.25, 0.07] | 0.01[-0.02, 0.06] | -0.85[-2.68, 1.03] | -0.07[-0.23, 0.08] | -14.16[-262.64, 234.33] |
| **IFGr_IFGl** | -0.10[-0.27, 0.06] | 0.00[-0.02, 0.03] | 0.22[-1.84, 2.26] | -0.09[-0.26, 0.06] | -0.77[-83.64, 82.10] |
| **IFGr_IFGr** | -0.11[-0.29, 0.08] | 0.01[-0.02, 0.07] | 0.74[-1.06, 2.60] | -0.09[-0.27, 0.08] | -12.55[-282.14, 257.06] |
| **IFGr_TPJl** | -0.07[-0.25, 0.09] | -0.00[-0.04, 0.02] | -0.48[-2.76, 1.68] | -0.08[-0.26, 0.09] | 3.12[-129.56, 135.84] |
| **IFGr_TPJr** | -0.08[-0.26, 0.08] | -0.00[-0.04, 0.03] | -1.91[-4.00, 0.31] | -0.09[-0.26, 0.08] | 1.79[-124.68, 128.27] |
| **TPJl_TPJl** | -0.13[-0.34, 0.07] | -0.00[-0.02, 0.03] | 0.01[-2.23, 2.31] | -0.13[-0.34, 0.07] | 0.00[-57.75, 57.75] |
| **TPJr_TPJl** | -0.12[-0.31, 0.06] | 0.01[-0.02, 0.07] | -1.02[-3.43, 1.35] | -0.10[-0.29, 0.08] | -10.02[-183.52, 163.44] |
| **TPJr_TPJr** | -0.16[-0.36, 0.04] | 0.01[-0.05, 0.10] | -2.27[-4.09, -0.40] | -0.14[-0.35, 0.07] | -11.50[-248.40, 225.40] |
| Treatment: Laughter Behavior during Free Interaction phase  Outcome: Prosociality (Money Sharing) | | | | | |
| ROI Pair | Direct Effect | Indirect Effect | Mediator Effect | Total Effect | Proportion Mediated |
| **IFGl_IFGl** | 1115.12[-619.27, 2851.00] | 66.56[-181.34, 540.11] | 0.99[-0.59, 2.52] | 1207.12[-554.51, 2962.94] | 5.50[-72.95, 83.98] |
| **IFGl_TPJl** | 1153.25[-485.87, 2789.48] | 3.99[-175.28, 259.47] | 0.36[-1.62, 2.41] | 1173.41[-495.46, 2785.53] | 0.34[-43.18, 43.86] |
| **IFGl_TPJr** | 1171.24[-513.33, 2832.63] | 3.60[-169.14, 265.14] | -0.40[-2.14, 1.31] | 1182.64[-533.65, 2820.42] | 0.29[-47.35, 47.96] |
| **IFGr_IFGl** | 1249.04[-412.67, 2938.98] | -1.57[-243.82, 201.55] | 0.34[-1.65, 2.25] | 1238.68[-424.98, 2943.64] | -0.13[-44.53, 44.28] |
| **IFGr_IFGr** | 1110.63[-699.26, 2867.09] | 71.23[-297.57, 656.39] | 0.51[-1.24, 2.31] | 1223.12[-610.44, 2980.94] | 5.82[-102.82, 114.48] |
| **IFGr_TPJl** | 1350.28[-216.59, 3005.77] | -1.41[-281.64, 243.14] | -0.56[-2.61, 1.46] | 1368.01[-272.96, 3038.68] | -0.10[-46.71, 46.50] |
| **IFGr_TPJr** | 1382.44[-287.04, 3037.86] | 28.97[-274.57, 447.55] | -1.38[-3.45, 0.55] | 1449.61[-282.61, 3066.74] | 2.00[-58.11, 62.11] |
| **TPJl_TPJl** | 1151.23[-807.37, 3143.74] | -3.20[-392.46, 311.33] | -0.41[-2.57, 1.76] | 1138.52[-796.48, 3170.01] | -0.28[-80.87, 80.29] |
| **TPJr_TPJl** | 1250.61[-677.56, 3159.75] | -127.99[-737.37, 218.78] | -1.56[-3.64, 0.70] | 1077.82[-890.77, 3084.11] | -11.88[-194.88, 171.13] |
| **TPJr_TPJr** | 1267.41[-861.25, 3461.03] | 225.86[-420.68, 1181.56] | -2.16[-3.91, -0.35] | 1552.76[-727.29, 3877.89] | 14.55[-122.48, 151.58] |
| Treatment: Condition  Outcome: Prosociality (Helping) | | | | | |
| ROI Pair | Direct Effect | Indirect Effect | Mediator Effect | Total Effect | Proportion Mediated |
| **IFGl_IFGl** | -0.01[-0.15, 0.12] | 0.00[-0.02, 0.02] | -0.02[-1.33, 1.30] | -0.01[-0.15, 0.12] | -0.08[-132.12, 131.96] |
| **IFGl_TPJl** | -0.01[-0.14, 0.11] | 0.00[-0.01, 0.02] | -0.43[-2.23, 1.37] | -0.01[-0.14, 0.11] | -6.87[-151.43, 137.66] |
| **IFGl_TPJr** | -0.03[-0.15, 0.10] | 0.01[-0.01, 0.05] | -0.86[-2.36, 0.59] | -0.01[-0.14, 0.11] | -73.12[-455.52, 309.29] |
| **IFGr_IFGl** | -0.00[-0.14, 0.13] | -0.00[-0.03, 0.01] | -0.47[-2.16, 1.13] | -0.01[-0.15, 0.12] | 18.33[-162.50, 199.16] |
| **IFGr_IFGr** | -0.04[-0.19, 0.10] | 0.01[-0.01, 0.07] | 0.91[-0.60, 2.44] | -0.02[-0.17, 0.13] | -70.43[-481.43, 340.56] |
| **IFGr_TPJl** | 0.00[-0.12, 0.13] | -0.01[-0.06, 0.01] | -1.72[-3.44, 0.01] | -0.01[-0.15, 0.12] | 140.46[-383.38, 664.32] |
| **IFGr_TPJr** | -0.01[-0.14, 0.12] | -0.00[-0.05, 0.04] | -2.44[-4.09, -0.83] | -0.01[-0.15, 0.13] | 24.44[-329.10, 377.99] |
| **TPJl_TPJl** | 0.00[-0.15, 0.16] | 0.00[-0.02, 0.03] | 0.76[-1.08, 2.52] | 0.00[-0.15, 0.16] | 31.64[-151.33, 214.61] |
| **TPJr_TPJl** | -0.00[-0.15, 0.15] | 0.00[-0.01, 0.05] | -0.81[-2.79, 1.00] | 0.01[-0.14, 0.16] | 77.20[-256.49, 410.87] |
| **TPJr_TPJr** | -0.01[-0.17, 0.15] | 0.00[-0.02, 0.05] | -0.99[-2.41, 0.43] | -0.00[-0.16, 0.16] | -238.66[-516.05, 38.71] |
| Treatment: Laughter Behavior during Free Interaction phase  Outcome: Prosociality (Helping) | | | | | |
| ROI Pair | Direct Effect | Indirect Effect | Mediator Effect | Total Effect | Proportion Mediated |
| **IFGl_IFGl** | 757.44[-637.82, 2182.40] | -0.78[-238.53, 242.27] | -0.03[-1.37, 1.33] | 749.07[-649.32, 2229.48] | -0.10[-92.89, 92.68] |
| **IFGl_TPJl** | 779.72[-599.15, 2154.60] | -3.53[-218.27, 165.73] | -0.37[-2.12, 1.39] | 762.77[-612.52, 2158.79] | -0.46[-76.82, 75.90] |
| **IFGl_TPJr** | 799.25[-565.44, 2181.98] | 11.86[-170.73, 291.39] | -0.72[-2.25, 0.73] | 833.78[-574.83, 2207.80] | 1.41[-84.50, 87.34] |
| **IFGr_IFGl** | 959.93[-511.08, 2374.90] | 1.25[-173.59, 205.90] | -0.31[-2.02, 1.35] | 963.11[-500.60, 2397.48] | 0.13[-51.10, 51.36] |
| **IFGr_IFGr** | 757.82[-809.48, 2385.17] | 119.55[-176.30, 653.54] | 0.76[-0.72, 2.30] | 910.19[-666.92, 2526.14] | 13.13[-140.81, 167.09] |
| **IFGr_TPJl** | 1155.28[-218.87, 2655.79] | -14.98[-437.52, 350.01] | -1.72[-3.36, -0.12] | 1131.23[-286.31, 2642.98] | -1.32[-113.57, 110.93] |
| **IFGr_TPJr** | 1089.08[-250.45, 2353.66] | 76.25[-374.33, 613.68] | -2.38[-3.94, -0.71] | 1162.68[-236.80, 2533.17] | 6.55[-94.37, 107.50] |
| **TPJl_TPJl** | 1196.18[-544.11, 2853.61] | 7.35[-281.43, 404.83] | 0.63[-1.22, 2.53] | 1223.39[-507.90, 2921.99] | 0.59[-68.43, 69.65] |
| **TPJr_TPJl** | 1195.28[-410.39, 2882.13] | -85.01[-621.64, 184.02] | -1.13[-3.01, 0.71] | 1063.07[-545.02, 2774.57] | -8.00[-154.93, 138.93] |
| **TPJr_TPJr** | 1573.89[-340.37, 3335.73] | 64.73[-241.65, 640.33] | -0.85[-2.45, 0.67] | 1663.45[-218.68, 3507.90] | 3.89[-53.24, 61.02] |
| Treatment: Condition  Outcome: Bonding | | | | | |
| ROI Pair | Direct Effect | Indirect Effect | Mediator Effect | Total Effect | Proportion Mediated |
| **IFGl_IFGl** | -0.19[-0.84, 0.51] | -0.00[-0.09, 0.08] | 0.09[-6.01, 6.56] | -0.19[-0.85, 0.51] | 0.11[-112.53, 112.76] |
| **IFGl_TPJl** | -0.25[-0.91, 0.42] | -0.00[-0.12, 0.07] | 2.46[-6.10, 11.43] | -0.26[-0.92, 0.39] | 1.65[-115.54, 118.85] |
| **IFGl_TPJr** | -0.40[-1.04, 0.21] | 0.11[-0.02, 0.36] | -7.85[-15.41, -0.53] | -0.27[-0.91, 0.34] | -42.22[-564.50, 480.05] |
| **IFGr_IFGl** | -0.36[-1.01, 0.26] | 0.02[-0.05, 0.20] | 4.31[-3.53, 12.00] | -0.32[-0.97, 0.31] | -7.65[-168.50, 153.21] |
| **IFGr_IFGr** | -0.31[-1.09, 0.48] | 0.14[-0.02, 0.46] | 7.21[-0.13, 14.89] | -0.15[-0.93, 0.64] | -90.35[-731.73, 551.00] |
| **IFGr_TPJl** | -0.12[-0.82, 0.57] | -0.02[-0.21, 0.07] | -3.54[-12.45, 4.98] | -0.16[-0.85, 0.53] | 14.77[-174.81, 204.37] |
| **IFGr_TPJr** | -0.07[-0.71, 0.56] | -0.02[-0.26, 0.21] | -12.00[-20.01, -4.06] | -0.10[-0.78, 0.58] | 21.30[-347.16, 389.79] |
| **TPJl_TPJl** | -0.13[-0.96, 0.64] | 0.02[-0.19, 0.27] | 8.32[-0.26, 16.85] | -0.10[-0.94, 0.70] | -22.14[-302.05, 257.75] |
| **TPJr_TPJl** | -0.20[-0.97, 0.59] | 0.04[-0.10, 0.29] | -3.77[-13.30, 5.81] | -0.13[-0.92, 0.65] | -29.28[-318.52, 259.93] |
| **TPJr_TPJr** | -0.10[-0.88, 0.66] | 0.04[-0.15, 0.35] | -6.84[-13.71, 0.59] | -0.03[-0.85, 0.77] | -121.70[-452.25, 208.84] |
| Treatment: Laughter Behavior during Free Interaction phase  Outcome: Bonding | | | | | |
| ROI Pair | Direct Effect | Indirect Effect | Mediator Effect | Total Effect | Proportion Mediated |
| **IFGl_IFGl** | 8041.42[1216.10, 14903.05] | -2.58[-1177.59, 1078.51] | -0.07[-6.41, 6.44] | 8046.70[1047.11, 14801.00] | -0.02[-23.07, 23.00] |
| **IFGl_TPJl** | 7037.11[172.29, 13799.84] | 21.61[-647.59, 1094.87] | 1.83[-7.10, 10.73] | 7127.64[283.62, 13931.67] | 0.29[-21.44, 22.05] |
| **IFGl_TPJr** | 6990.94[247.17, 13488.52] | 184.12[-1165.50, 1973.26] | -5.84[-13.06, 1.22] | 7276.84[383.13, 13997.95] | 2.52[-32.86, 37.92] |
| **IFGr_IFGl** | 6788.75[-119.04, 13269.73] | -37.37[-1438.88, 1103.94] | 4.59[-3.42, 13.12] | 6704.97[-328.51, 13242.14] | -0.56[-37.86, 36.75] |
| **IFGr_IFGr** | 5021.10[-2847.09, 12971.34] | 945.19[-590.16, 4044.44] | 5.54[-2.05, 13.35] | 6125.80[-1978.45, 13999.37] | 15.42[-99.59, 130.44] |
| **IFGr_TPJl** | 6668.10[-456.40, 13739.57] | -6.62[-1251.20, 1019.58] | -3.18[-11.55, 5.49] | 6638.51[-563.24, 13753.55] | -0.10[-33.17, 32.97] |
| **IFGr_TPJr** | 5863.33[-694.22, 12348.93] | 412.24[-1804.32, 3092.53] | -12.17[-20.45, -4.24] | 6282.39[-606.34, 13223.82] | 6.55[-76.20, 89.32] |
| **TPJl_TPJl** | 6940.81[-1191.65, 15032.09] | 144.94[-1842.99, 2738.54] | 6.60[-2.53, 15.54] | 7237.39[-1066.34, 15704.25] | 2.00[-64.45, 68.45] |
| **TPJr_TPJl** | 9814.63[1720.58, 17792.49] | -313.92[-2599.68, 957.18] | -4.55[-13.71, 5.02] | 9315.81[1105.52, 17580.34] | -3.37[-42.22, 35.49] |
| **TPJr_TPJr** | 8517.28[87.01, 17324.93] | 523.72[-1276.06, 3722.28] | -5.89[-12.95, 1.49] | 9248.13[811.21, 18141.99] | 5.66[-36.82, 48.14] |

Table 18. Posterior estimates from Bayesian mediation models for Synchrony during the free interaction phase for HbR data. Social outcome variables (Liking, Prosociality (Money Sharing, and Helping), and Bonding) by ROI Pair. Separate models were computed for each ROI pair (as the mediator) with either the Laughter Condition (Laughter experience vs. no Laughter experience) or the Laughter Behavior during the free interaction phase as the predictor (treatment). Posterior means and 95% Equal-Tailed Intervals (ETIs) are reported.
